# Supplementary material for: Effects of Tai Chi on anxiety and theta oscillation power in college students during the COVID-19 pandemic: A randomized controlled trial
Source: PLoS One. 2024 Nov 1;19(11):e0312804. doi: 10.1371/journal.pone.0312804 (PMC11530040; doi:10.1371/journal.pone.0312804)
Supplement: S1 File — (PDF) [file pone.0312804.s001.pdf]

Thesis Proposal

Effects of Tai Chi on Cognitive Performance Mental Health and Physical Fitness of College Students with a Sedentary Lifestyle

MIN WANG

PhD student in Programs of Exercise and Sport Sciences

2021

LIST OF ABBREVIATIONS

PA	physical activity
COVID-19	novel coronavirus disease 2019
RCT	randomized controlled trials
CF	cognitive function
PF	physical fitness
TC	tai chi
EEG	electroencephalogram
PFC	prefrontal cortex
MC	the motor cortex
OC	occipital cortex
VBM	voxel-based morphometry
GMV	gray matter volume
MMSE	Mini-Mental State Examination
MDRS	Mattis Dementia Rating Scale
P300	event-related potential 300
SCWT	Stroop Color and Word Test
HRV	heart rate variability
rs-EEG	resting-state electroencephalogram
WHO	World Health Organization
fNIRS	near-infrared functional brain imaging
SB	sedentary behavior
VPA	vigorous physical activity
STAI	State-Trait Anxiety Inventory
DSI	Depression State Inventory
MoCA	Montreal Cognitive Assessment
TMT	Trail Making Test
LM	Logical Memory

MDRS	Matisse Dementia Scale
rs-fMRI	resting-state functional magnetic resonance imaging
DEXA	dual-energy X-ray absorption assay
CT	computed axial tomography
MRI	magnetic resonance imaging
BIA	bioelectrical impedance analysis
GAD	generalized anxiety disorder
PTSD	post-traumatic stress disorder
OCD	obsessive-compulsive disorder
IPAQ	International Physical Activity Questionnaire
PARQ	Physical Activity Readiness Questionnaire
CRF	case report form
LF	low frequency
HF	high frequency
BPM	beat per minute
SDNN	standard deviation of normal to normal R-R intervals
RMSSD	root mean square of successive differences of R-R interval

TABLE OF CONTENTS

Chapter I: Introduction

1.1 Background and rationale 1

1.2 Scope of the study 8

1.3 Research questions 9

1.4 Objective of this study 9

1.5 Study hypothesis 10

1.6 Significance of the study 10

1.7 Conceptual framework 10

Chapter II: Literature Review

2.1 Definition of relevant concepts and operational definitions 12

2.2 Real Dilemma: Nowadays college students face major problems 17

2.3 The definition, types and mechanism of tai chi 24

2.4 The effectiveness and benefits of Tai Chi on health 29

2.5 Summary 35

Chapter III: Research Methodology

3.1 Study design overview 36

3.2 Recruitment and sample 38

3.3 Experiment content and test sequence 41

3.4 Experimental apparatus, procedure and measure assessment 43

3.5 Tai chi intervention and control group treatment 53

3.6 Statistical analysis 55

TABLE OF CONTENTS

Reference 57

Appendix A International Physical Activity Questionnaire (IPA-Q) 82

(Chinese Version) 84

Appendix B Physical Activity Readiness Questionnaire (PAR-Q) 85

(Chinese Version) 86

Appendix C Depression Status Inventory 87

(Chinese Version) 89

Appendix D State-Trait Anxiety Inventory for Adults 91

(Chinese Version) 92

Appendix E Flow chart of the major stages of an EEG study 95

Appendix F Autonomic heart rate variability (HRV) data acquisition equipment

9 6

Appendix G Physical Fitness data acquisition equipment 100

Chapter I

Introduction

1.1 Background and rationale

National development depends on talents (Shen, 2021), and college students are a country's precious talents. As the old Chinese saying goes, "the strong young people make the nation strong". Next, with the advancement of technology, economic and medical standards, the quality of life and consumption-ability of the Chinese people have a large improvement. However, it also brings convenience and diversification of nutritional intake, fast-paced life, high learning pressure, so that the number of sedentary people is increasing year by year (Pan & Bi, 2009), and patients are getting younger (Lu, 2017). Due to the risk of adolescents increasing (Li, 2019), medical resources will be exhausted, and public health and national development will also be affected (Koteles & Simor, 2014).

Studies have confirmed that insufficient physical activity has caused many chronic diseases to develop at a younger age (Liu, 2020), together with mental health problems, they have become two major global public health problems (Guthold et al., 2018; Kessler et al., 2007). According to statistics, the detection rate of insufficiency of physical activity among 11-17 years old in school worldwide has reached 81%. The problem of insufficient physical activity of Chinese adolescents is even more serious, and the detection rate is as high as 84.3% (Guthold et al., 2020). During the period from 2000 to 2014, Data of the Fourth National Physical Fitness Monitoring in China showed that "the overall level of the detection rate of insufficient physical activity (PA) among adults aged 20-59 in China is about 78%" (Tian et al., 2016).

In addition, the novel coronavirus disease 2019 (COVID-19) epidemic has led to increased community environmental risks, further reducing the willingness of young people to go out and exercise (Xiang, 2020; Shen, 2021). Physiologically, sedentary or lying downtime increased due to lifestyle behavior changes (Palermi, 2020), or reduced willingness to exercise lead to changes in weight gain, body composition,

cardiopulmonary fitness (Korczak, 2017), and physiological symptoms, such as muscle, bones, sensory nerves, and brain cognitive nerves, etc. (Alexandr, 2016; Sawka, 2011). Psychologically, a lack of interpersonal and social communication can easily lead to anxiety, depression, emotional instability, and increase psychological pressure.. In the long term, it will further lead to adaptive disorders and may induce cardiovascular diseases (Baranov, 2006).

Studies have also shown that physical exercise improves cognitive performance. For example, compared with Diponegoro University medical students who did not do any physical exercise (n=15), the 6-week High-intensity interval training intervention group (n=14) had significantly lower reaction time scores (p=0.007) (Saphira, 2021); delaying cognitive decline, for example, in a review of randomized controlled trials (RCT), compared with 1,508 elderly control groups with impaired cognitive function (CF), 1,780 patients with impaired CF under PA intervention among elderly participants, 37.0% of the intervention group showed a statistically significant beneficial effect of PA on CF, and 40.0% of the control group showed a significant reduction in CF (Sontakova, 2021);

Improving mental health, for example, at the peak of the COVID-19 outbreak, a longitudinal survey found that PA can directly relieve general negative emotions in 66 Chinese college students, and the alleviated effect is greatest when the activity is about 2500 MET (Zhang et al., 2020);

Improving body composition, for example, in healthy, untrained college students (n=38), after 8 weeks of resistance (n=22) and aerobic (n=16) training, it was found that compared with aerobic training, the resistance training group increased fat significantly Percentage (2.0±2.0 %, p<0.001) (Morrow et al., 2021);

Improving physical fitness (PF), for example, 1414 (completion rate 94.3%) Tsinghua University students used the International Sports Activity Questionnaire and found that college students who were physically inactive had a 1.25 times higher risk of obesity than those who were physically active in 2012. At the same time, the failure probability of standing long jump and grip strength test was also very higher, increased by 1.39 times and 2.39 times respectively, and the score of physical fitness

test was also lower (Wang, 2019).

However, in fact, owing to the difference in the requirements of sport equipment, environment and methods, the convenience of sport participation (Idiegbeyanose, 2019); and the perceived difference in the intention of improving body appearance, the comfort of sports experience, and the cost and time factors (Lee, 2019), the willingness of college students to exercise keeps falling. For example, about one-third of student-athletes had a declining tendency to participate in sports after receiving university higher education (Hossen et al., 2020). In addition, PA decreases significantly during the transition from youth to early adulthood, and PA decreases the most during university education (Kwan et al., 2012). One-third of active senior middle school students became insufficiently active during the transition to college life (Bray & Born, 2004).

Therefore, it is necessary to make exercise decisions that take less time and cost and have significant effects, which can not only reduce the barriers to exercise participation, but also meet the goals of youth exercise needs, and help improve their current cognitive performance, fitness and mental health (Xiang, 2020; Palermi, 2020; Korczak, 2017; Alexandr, 2016; Sawka, 2011; Jimenez-Pavon, 2020).

Tai Chi (TC), as well known as Taiji or Tai Chi Quan/Chuan, has been variously described as a meditation or mind-body exercise or internal martial art originating in China. Since TC is a low-impact, low-cost mind-body exercise, it has become an inexpensive intervention and alternative medicine.

In the past decade, clinical researchers have investigated the multiple components of TC. Wayne et al. deconstructed the multiple components of TC as follows: (1) active relaxation; (2) intention, belief, and expectation; (3) natural, freer breathing; (4) structural integration, dynamic form, and function; awareness, (5) strengthening and flexibility; (6) mindfulness, and focused attention; (7) embodied spirituality, philosophy, and ritual; (8) social support, interaction, and community.

These components may work independently and synergistically to generate benefits for adolescent's cognitive performance, mental health, and PF (Wayne et al., 2013). For example, the mindfulness of TC, like meditation, helps to cognition; both

strengthening and flexibility help to fitness; social support, interaction and community are helpful for mental health.

Pan summarized extant studies that used brain imaging techniques and electroencephalogram (EEG) to examine the effects of TC on older adults. The findings suggested that the benefits of TC intervention on neural plasticity in the brains of healthy, ether delaying or reversing the rate of neurological degeneration (Pan et al., 2018). Xie used dynamic Bayesian inference and wavelet-based coherence analysis methods to calculate the functional connections and effective connections in the brain between the TC practitioners with experienced Chen-style and the control group who demographically matched naive TC practitioners. The results showed that TC training improved the connection of motor cortex (MC), prefrontal cortex (PFC) and occipital cortex (OC) in myogenic activity, endothelial cell and sympathetic nervous system metabolic activity; as well as enhanced brain function connections and relayed TC can improve cognition and resist memory decline potential (Xie et al., 2019).

Compared with the control group, the participating elderly TC group was matched in terms of gender, age, and PA levels. In terms of behavior, Liu found that the elderly TC group showed greater emotional stability, higher levels of meditation, and lower risk -- taking trends in sequential risk-taking using voxel-based morphometry (VBM). In addition, the founding showed that the gray matter volume (GMV) of the hippocampus and thalamus was larger in the TC exercise group. Notably, GMV in the thalamus was clearly correlated with both emotional stability and meditation level. For example, independent sample T-test showed that the TC group had a higher emotional rating for bad results than the control group, while the TC group had a higher emotional rating for good results than the TC group and a lower emotional rating for good results. In addition, the K value of the TC group was lower than that of the control group, indicating that the TC group had stronger emotional stability compared with the control group. In addition, GMV of the left thalamus and left hippocampus in the TC group was larger than that in the control group (Liu et al., 2019).

Cui and colleagues used the Voxel-based morphometry toolbox and CONN toolbox, compared to the control group, found that significant FC increases in the left superior parietal lobule in the TC group after the 8-week practice in college students. Moreover, pre - and post-exercise comparisons of GMV among the three groups showed that GMV was significantly increased in the left superior temporal gyrus, left precuneus, left middle occipital gyrus and right middle temporal gyrus in the TC group. Lastly, it is concluded that compared with general aerobic exercise, 8 weeks of TC exercise has a stronger impact on the brain plasticity of college students, which is reflected in the increase in the GMV of the left superior temporal gyrus, left middle occipital gyrus, and right middle temporal gyrus. and the functional connection between the left upper lobuleand the left middle frontal gyrus is enhanced (Cui et al., 2019).

The above results all show that TC was beneficial to everyone in terms of meditation, spontaneous cognition, memory retrieval, and attention to depression (Raichle, 2015; Ives-Deliper, 2011). At the same time, about 40% of adults in the US used alternative and complementary medicine (Harris et al., 2012), and about 25% of them participate in mind-body activities (Chacko et al., 2014). Mind-body activities are considered to be a therapeutic practice that "focuses on the interactions among the mind, brain, behavior, and body, with the intent of overall health" (Elkins et al., 2010). For example, five systematic reviews evidence that TC can improve cognitive performance.

A meta-analysis found that most supplement interventions, such as soy isoflavone supplements, Mediterranean diet, olive oil, nuts, did not have any cognitive outcomes for middle-aged and elderly people without mild cognitive impairment or dementia Significant effect, but TC exercise had a significant positive impact on global cognition (Lehert et al., 2015).

Zheng reviewed 9 studies, including 4 RCTs and 5 non-RCTs, and identified a total of 632 participants. Three studies used the Mattis Dementia Rating Scale (MDRS), Mini-Mental State Examination (MMSE), or event-related potential 300 (P300) to measure global cognitive function; Three studies used hands and feet

alternating movement time, response time or the MDRS attention score to measure attention; Three studies used Wechsler Adult Intelligence Scale, MDRS memory score, or Auditory Verbal Learning Test to measure learning and memory; One study used mental rotation and arm stability to measure emotion and perception; and Four studies used Stroop Test, Trail Making Test, and Clock Drawing Test to measure execution. TC has shown a positive impact on most results in various cognitive domains. Compared to common physical activities, TC also showed a potential protective effect on the cognitive ability of healthy adults (Zheng et al., 2015).

Miller and Taylor-Piliae reviewed studies about cognitive function and TC in community-dwelling elderly, and found that 10 of the 12 studies reported improvements in executive function, learning, language, and/or memory, as well as concluded that TC is a suitable exercise for elderly and seemed to provide positive cognitive benefits (Miller et al., 2014).

A meta-analysis found, compared with the control group, the TC group had an improved attention ($P<0.001$) and processing speed ($P<0.001$) (Kelly et al., 2014); even compared to the active control group, the TC group had a moderate effect size (Wayne et al., 2014). In addition, three recent reviews suggested that TC may have some beneficial effects on the mental health of patients with depression (Wang, 2014; Chi, 2010; Wang, 2010). Moreover, TC was beneficial to reducing the severity of depression, stress, anxiety and improving leg strength in centrally obesity population with depressive symptoms (Liu et al., 2015).

Studies have shown that TC helps to improve cognitive performance, promote physical and mental health, and improve PF (Irazusta et al., 2006; Burhanuddin et al., 2021). However, most current studies on TC focus on the elderly and chronic patients (Wei et al., 2020; Liu et al., 2020; Song et al., 2014).

Although TC is more traditional and not fashionable enough, and it may not appeal to adolescents (Wall, 2005), college students have TC course assessment requirements in most Chinese universities. Another very important reason, the intelligence of college students is at the highest level, but multiple cross-sectional research showed fluid abilities was showing a steady decline trend from age 20 to 80

(Salthouse, 2010), so it is also necessary to prevent cognitive aging in a safe and inexpensive way in college students.

The neural mechanism of TC intervention was analyzed (Pan et al., 2018; Xie et al., 2019; Cui et al., 2019; Yu et al., 2018), and studied their role in improving cognitive performance (Lu et al., 2016; Sungkarat et al., 2017) and PF function (Kong, 2019). On the other hand, studies on TC mostly focus on cross-sectional controlled studies, and lack of comparative studies on random control groups. In the current COVID-19 epidemic, only a little research is focusing solely on the impact of TC on mental health and PF (Mao et al., 2021; Solianik et al., 2021; Zulkifli et al., 2021).

However, Liang examined the acceptability and feasibility of remotely provided home-based exercise programs for the well-being and physical function of elderly self-isolated during the COVID-19 pandemic and found all intervention groups saw increased physical function at follow-up (Liang et al., 2020). Moreover, Xiang selected the 10 most common home-quarantine PA, including walking, housework, jumping, running, resistance training, stretching, yoga, bodybuilding, TC and ball sports (such as basketball, football, volleyball). It found, during COVID-19 outbreak of home isolation, TC was one of the most frequent types of PA for college students (Xiang et al., 2020).

In addition, as we all know, most of the tuition fees of college students are either supported by their parents or by working part-time. However, the COVID-19 pandemic has already become a worldwide disaster. On the one hand, the COVID-19 has become a worldwide crisis. This pandemic not only affected the world economy but also the family economy.

On the other hand, many students reported financial difficulties because of the epidemic. 34% of students reported that due to the current epidemic crisis, they had to be unemployed, reduced working hours, or did not pay for the work done. Similarly, 22% of students said that their parents were less able to support their studies financially. When asked how the current crisis affects their finances, 30% of the respondents said that their ability to pay tuition has become worse, and 8% of them said that their ability to pay tuition has become even lower (Montacute & Holt-White,

2020).

However, TC is now becoming an increasingly popular mind-body exercise, which encourages mindful attention to the body during exercise. It combines low-impact movement with diverse cognitive skills, including somatic awareness, mental focus, goal setting, task shifting, and visualization. It is considered a low-cost and safe complementary therapy that millions of people practice for various purposes. Therefore, TC has become an inexpensive intervention and alternative medicine for young adults in the COVID situation.There are some major styles of TC, including Chen style, Yang style, Wu / Martial style, Sun style, Wu style, and Simplified 24 form style. ALL former styles are effective in improving cognitive function and fitness (Zou et al., 2019). However, All of the above types style are more complex than 24 form style and 24 form style TC is the most popular in all ages of people in China; thus, the 24 form TC will be tried in the present work.

In addition, no previous research has investigated the link of combined effects of TC with cognitive performance, mental health, body composition, and PF in one and the same research. Therefore, this current research will be aimed at studying the effects after the single practice of TC exercise on participants' cognitive performance with a within-subject design, and the long-term effects of 12 weeks of TC exercise on participants' cognitive performance, physical and mental health with a two-arm, randomized, parallel controlled trial design. The results of this study might be helpful to further expand and deepen the research field and provide guidance for the government, educational institutions, instructors, and individual students with a sedentary lifestyle under the COVID-19 pandemic.

1.2 Scope of the study

The scope of this study includes the 1st and 2nd study.

The 1st study is to determine the effects after single practice of TC. As we all know that TC is a safe neurobiological complementary therapy, however, throughout the course of life, both function and organization of the human brain are plastic (Kurth et al., 2015), and some of the neurobiological effects appear to be partially

reversible (NIDA, 2021). Hence, it is necessary to determine the effects after the single practice of TC exercise with a within-subject design in participants already receiving TC exercise groups.

The 2nd study is to evaluate whether the long-term effects of 12 weeks of TC training can be used as exercise intervention to improve cognitive performance, mental health, and PF by comparing within two-arm, randomized, parallel controlled groups. In addition, a follow-up evaluation will be conducted in the fourth week.

1.3 Research questions

The 1st research question is whether single practice TC has a positive effect on cognitive performance and will be assessed using Stroop Color and Word Test (SCWT) and heart rate variability (HRV) tests?

The 2nd research question is that can 12 weeks of TC improve cognitive performance, mental health, and PF in sedentary college students?

1.4 Objective of this study

This study aims to evaluate the effectiveness of TC on cognitive performance, mental health and PF of sedentary college students.

The primary aims of this study are to evaluate the effect of a 12-week 24-Form TC training in college students with a sedentary lifestyle due to COVID-19 and to explore the underlying mechanism based on SCWT, HRV and resting-state electroencephalogram (rs-EEG).

The secondary aims of this study are as follows:

To investigate the effect of TC on specific domains of body composition and Physical Fitness in college students with a sedentary lifestyle due to COVID-19; And to explore the mechanism of action of TC on mental health, especially in depression and anxiety, in college students with a sedentary lifestyle due to COVID-19 based on HRV.

Meanwhile, the tertiary aims of this study are as follows: To investigate the effect of single practice TC on cognitive performance in college students with a sedentary

lifestyle based on SCWT and HRV test.

1.5 Study hypothesis

Hypothesis 1: It exists additive effect of TC, and single practice of TC intervention can affect the outcome parameters of the related physiological experiment, and deserve well of SCWT and HRV performance.

Hypothesis 2: TC can be used as an exercise intervention to improve cognitive performance, mental health, and PF in the 2nd study, and the 2nd study will be conducted by comparing the parameters related to the outcome before and after performing 12 weeks of TC between the two groups.

1.6 Significance of the study

The outcome of this study can be used as the primary evidence that TC exercise is a benefit to the cognitive function or improves the cognitive performance of sedentary college students.

This knowledge may provide an alternative treatment to the mental health problems in sedentary college students, which can improve their physical and mental health, regulate psychological stress; And provide an alternative treatment to PF in sedentary college students, which can improve physical composition and PF. Finally, this study will be beneficial for home exercise promotion and enrichment during the COVID-19 epidemic.

TC may also be beneficial to improve physiological performance after single practice of TC intervention. And this study would provide theoretical and clinical guidance for research related to parameters after TC intervention.

1.7 Conceptual framework

As mentioned above, sedentary college students under the COVID-19 environmental risk give rise to a series of problems. The purpose of this study is to show that TC can have a positive effect on these problems. Some questions are to be demonstrated in the present study as shown in figure 1.

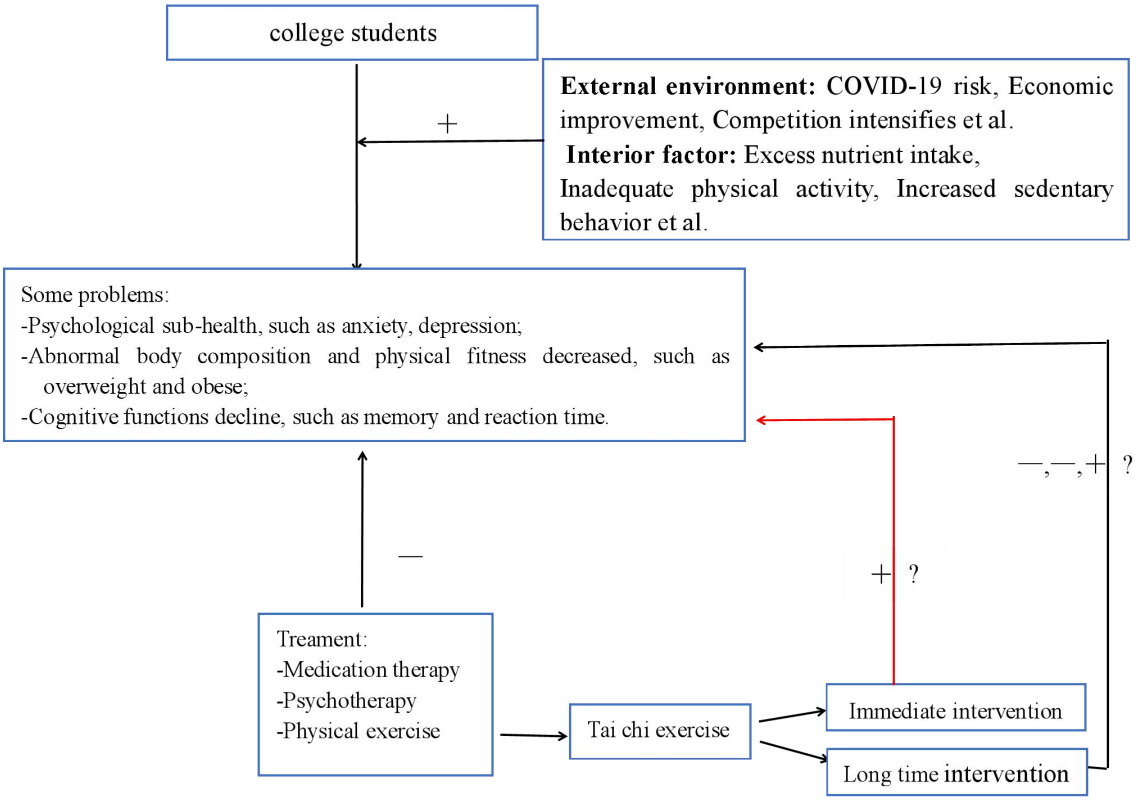

Figure 1 Conceptual framework

Chapter II

Literature Review

This chapter presents a literature review of the history and current development of research on TC and cognitive ability and psychological adaptation ability of (sedentary)college students, to provide a wider context of research. Due to the small sample of college students, it cannot comprehensively reflect the effects of TC. Therefore, the research object of this review is extended to all populations.

2.1 Definition of related concepts and operational definitions

2.1.1 Sedentary behavior

A sedentary lifestyle is a lifestyle that involves little or no PA. People with sedentary lifestyles often sit or lie down for most of the day and engage in activities such as playing video games, using mobile phones/computers, watching TV, socializing, or reading (Physical Activity Guidelines Advisory Committee, 2018; Owen, Neville, et al., 2020).

Sedentary behavior (SB) is different from lack of PA. SB is characterized by any conscious behavior with an intensity of $\leq$ 1.5 METs in a reclining or sitting position (Tremblay et al., 2017). Sitting time is a subcomponent of SB and a common measure of SB (Healy et al., 2011). Mclaughlin (Mclaughlin et al., 2020) found that the average daily median sitting time for all countries/regions is 4.7 (interquartile range: 3.5-5.1) hours, accounting for 47% of the global adult population. Sitting time is longer in high-income countries than in low-income countries (4.9 hours vs 2.7 hours).

SB is associated with a series of adverse health outcomes, including but not limited to: anxiety (Teychenne, 2015; WHO, 2010), depression (WHO, 2010; Daniel, 2010), obesity (CDCP, 2016), hypertension (WHO, 2010), cardiovascular disease (WHO, 2010; Patterson et al., 2018). In addition, these diseases will also bring some complications, such as cognitive decline or aging, abnormal body composition, and decline in PF.

2.1.2 Mental health, depression, and anxiety

"Mental health is a condition of happiness, in which individuals are aware of their abilities, can cope with normal life pressures, can work efficiently, and can contribute to the community" (WHO, 2018). WHO stresses that mental health is "more than the absence of mental disabilities or disorders". Mental health refers to emotional health, cognitive and behavioral. It depends on how people feel, think, and behave. People sometimes use "mental health" to express the absence of mental disorders. Mental health is often affected by many conditions, such as anxiety, depression and stress.

Anxiety is a feeling that is characterized by an unpleasant state of inner confusion, and generally accompanied by neurological behaviors such as physical complaining, pacing back and forth, and contemplation (Seligman et al., 2003). It includes subjectively unpleasant fear of anticipated events (Davidson, 2008). Anxiety is an emotion of worry and restlessness, usually, overreaction, and lack of attention to situations subjectively considered threats (Bouras & Holt, 2007). It is often accompanied by inability to breathe, muscle tension, tight abdomen, irritability, inattention, and fatigue (APA, 2013).

The State-Trait Anxiety Inventory (STAI) is extensively used state and trait anxiety scale (Spielberger et al., 1983), and used to distinguish it from depression syndrome, as well as diagnose anxiety in a clinical setting. It's also seen as a caregiver suffering indicator in research (Greene et al., 2017; Ugalde et al., 2014).

Depression is an emotion of dislike to activity and low mood, it could impact individual feelings, thoughts, motivation, behavior, and happiness (Zwart et al., 2019). The core characteristic of depression is said to be anhedonia, a loss of interest or pleasure in certain activities that usually bring pleasure to people (Gilbert & Paul, 2007). Depression has certain mood disorders symptoms, such as dysthy or major depression (Harper, 2014); this is a natural temporary response to events in life, for instance the lost loved one; this is also a symptom of physical illnesses, and also medical and some drugs side effects. It may be difficult to concentrate or think, and shows sadness, as well as a significant decrease or increase in sleep time and appetite.

People who have depression may feel hopeless, depressed, and even sometimes suicidal. It could be long-term or short-term.

Depression as a measure of mood disorder includes but is not limited to: Baker Depression Scale, Patient Health Questionnaire, and Depression State Inventory (DSI) with 9 depression scales (Cox et al., 2012). The above measures are all psychological tests which ask participants about their problems and are mainly used to evaluate the degree of depression.

2.1.3 Cognition and evaluation

Cognition is the level of knowledge that can be distinguished from the experience of emotion and intention (Encyclopedia, 2021). Cognition is that "the mental process or behavior of getting understanding and knowledge through senses, experience, and thoughts" (Oxford University Press and Dictionary, 2020). It includes but is not limited to processes and functions of intelligence, such as problem-solving and decision-making, judgment and evaluation, memory and working memory, knowledge formation, language production, attention, calculation, understanding, and reasoning. The process of cognitive activity usually applies existing knowledge and generates new knowledge, and absorbs new knowledge to make decisions.

Generally speaking, different cognitive functions have a role in these different cognitive processes, such as attention, perception, memory, reasoning. Every cognitive function converges and works together and generates new knowledge to interpret the world (David, 2021).

Cognitive function assessment uses a variety of methods, and different studies have different types of cognitive tests and fields. Cognitive functions include common cognitive performance, psychomotor performance, intellectual function, executive function, visual structure, spatial perception, speed, attention, orientation, memory, visual and language (Prickett et al., 2015).

The assessment of cognitive ability is mainly based on cognitive tests or questionnaires, such as the Mini Mental State Examination (MMSE), Trail Making Test (TMT), Montreal Cognitive Assessment (MoCA), Logical Memory (LM),

Matisse Dementia Scale (MDRS), NIH Toolbox, Flanker test, and SCWT.

With the development of anthropology, philosophy, psychology, and neurology, cognitive psychology began to find in-depth knowledge of the behaviors that deal with the influence of information and what mental processes related to different relationships have been acquired. Since the 1960s, people's interest in cognition and cognitive skills has grown exponentially (David, 2021). New techniques, including but are not limited to blood oxygen level-dependent functional magnetic resonance imaging (BOLD-fMRI), resting-state functional magnetic resonance imaging (rs-fMRI), EEG, have been commonly used in current research.

For example, event-related potential (ERP), magnetoencephalography, and EEG, which can detect the electrical or magnetic signals of the brain in response to stimulation, with high temporal resolution and low spatial resolution, used to determine the functional areas of the brain; usually, EEG is often used to record the electrophysiological brain activity index, which can generally reflect the electrophysiological activities of brain nerve cells in the cerebral cortex by recording the changes of the waves during brain activity (Zhang & Wang, 2008). Another example, hemodynamic-based functional magnetic resonance imaging (fMRI), near-infrared functional brain imaging (fNIRS), single positron emission tomography, and positron emission tomography can detect brain processing and have a good spatial resolution, but not as good as the time resolution of EEG. fNIRS technology and fMRI technology have a common neurophysiological basis, that is, neurovascular coupling mechanism. When the brain is stimulated by clues or performs tasks, the local blood flow and oxygen metabolism rate of the corresponding brain regions in the activated state will increase significantly, and oxyhemoglobin and deoxyhemoglobin will change accordingly. fMRI is used to measure cerebral blood flow changes caused by neuronal excitement through MRI, and predict the functional state of the cerebral cortex and the signal processing process of subcortical layers such as ventral striatum, globus pallidus, thalamus, and hypothalamus through oxygen. The cerebral blood level depends on the index. The fNIRS mainly relies on the attraction of near-infrared light and the absorption and scattering of materials by brain

tissue. According to Lambert-beer law of activating brain regions, hemorrhage oxygen and blood volume changes are calculated to reflect the eating behavior and the brain. Cortical hemodynamic changes are closely related, such as the prefrontal cortex, medial orbitofrontal cortex, temporal cortex, and inferior frontotemporal cortex (Gao et al., 2018).

2.1.4 Body composition and physical fitness

According to different body composition, the human body model can be divided into five classes: whole body, tissue system, cell, molecule and atom (Wang et al., 1992). The body's components can be water, protein, lipid, glycogen, and minerals in the molecular class, and the sum of the above substances is weight. Percent body fat is the percentage of body fat to total body weight. As for obesity, there is no universally accepted definition in the academic world, but most researchers currently consider the level of obesity as 25% body fat in men and 30% body fat in women (Okorodudu et al., 2010). In addition, these body models are also named by the number of components, such as multi-component models, 3-component models, and 2-component models (Fogelholm & Van Marken Lichtenbelt, 1997).

PF is a healthy and suitable state, which is different from body composition, more specifically, the ability to perform daily activities, working, and sports. PF is usually connected with proper nutrition (Tremblay, 2010), moderate-intensity physical exercise (Groot et al., 2011) and adequate rest (Malina, 2010). PF can be measured based on different tests and indicators such as reaction time, grip strength, sitting and extension, balance, push-ups/sit-ups, vertical jumps, vital capacity, blood pressure, and resting heart rate.

There are various technologies currently used to detect body composition, including dual-energy X-ray absorption assay (DEXA), whole-body K counting, magnetic resonance imaging (MRI), bioelectrical impedance analysis, computed axial tomography (CT), bioelectrical impedance analysis (BIA), isotope dilution, thermal imaging, plethysmography, densitometry, anthropometry, and ultrasound. BIA, using the bioelectrical impedance method analysis body composition, will be used in this

study. Although its effectiveness is also affected by gender, medical conditions, size, race, and age, however, BIA is portable, non-invasive, inexpensive and easy to use.

2. 2 Real dilemmas: nowadays college students face major problems

In March 2020, the World Health Organization (WHO) announced COVID-19 after the outbreak as a pandemic (WHO, 2020). to curb the spread of this infection, countries around the world to take various measures, including the National blockade, campus close, online lectures and postponement of elective programs. These have led to psychological effects on different groups of people, especially disadvantaged student groups (Kuy et al., 2020). To break the chain of social transmission, according to evidence collected from past influenza outbreaks, institutions have extended the suspension of classes even if various lockdown measures are canceled. The psychosocial effects of the pandemic and social isolation on children are numerous, including sleep problems, depression, and anxiety symptoms, etc. (Chawla et al., 2021). In addition, these lockdowns affect people's travel, entertainment, work, and education, as well as subsequent PA and SB.

Therefore, understanding the changes of SB and PA during the lockdown is significant for health outcomes, including some outcomes related to these behaviors (Stockwell et al., 2021).

2. 2. 1 Frequent psychological problems among college students

Mental health issues dealt a heavy blow to students. Psychological problems are very popular in college students (Blanco et al., 2008). It may be because going to college is a challenging time for many traditional and non-traditional undergraduates. Traditional college students start college after graduating from high school, are usually younger, rely on their parents for financial support, and do not work or part-time (Statistics, 2013). Therefore, in addition to the pressures associated with academic burdens, these college students also have to deal with more adult responsibilities without mastering adult skills and cognitive maturity. For example, many traditional college students may be facing potentially stressful experiences for

the first time, including work, romantic relationships, or long-term exposure to different cultures and beliefs (Arnett, 2000).

In addition, during the COVID-19 pandemic, the mental health of children and college students will undoubtedly undergo great changes. It is obvious from some current research data that the epidemic has affected the mental health of children and college students. In the face of a major epidemic, the emotional changes of students are ups and downs, which can be summarized into four types: anxiety, discrimination, hypochondria, and collapse (Liu, 2020). In addition, during the epidemic prevention and control measures required to reduce social events, young people perceived social support reduction, increased loneliness, anxiety, depression, and other mental illness increased risk (Lee et al., 2020; loaded et al., 2020). Children during home isolation are prone to anxiety, panic, and other bad emotions (Li et al., 2020). Many teachers have also noticed the changes in the mental state of children and college students in their teaching practice. For example, after the beginning of school in some areas, many teachers found that the attention of students has dropped significantly, and the concentration on learning has dropped. The reason is that their mind control ability has declined. This shows that during the epidemic, their self-control ability was affected by the surrounding environment and the epidemic, and has not been further strengthened (Li et al., 2020). Conversely, they were greatly affected. In addition, the impact of major epidemics on the mental health of children and college students is also reflected in other aspects. For example, there will be a certain deviation in the cognition of things and the cognition of society. Some children and college students affected by the epidemic often think that this major epidemic has had a great impact on society and life. This caused a certain degree of dissatisfaction or pessimism in their thoughts. Some children and college students even reject communication and are afraid of going out (Gao, 2020).

Most mental health disorders peak at the onset of youth. Kessler observed that by the age of 25, 75% of people with mental illness will have the first disease. Among traditional students, major disturbances associated with attending college may exacerbate and/or trigger the first episode of psychopathology that is currently present

for the first time in childhood. Similarly, non-traditional students may have to meet the requirements of their many roles (work and family), and their symptoms may worsen or relapse (Kessler et al., 2007).

Anxiety disorder is one of the most common psychological problems among college students, with about 11.9% of college students suffering from anxiety disorder (Blanco et al., 2008). Among anxiety disorders, the age of onset of social phobia is earlier, the average age of onset is between 7-14 years old, while the onset time of post-traumatic stress disorder (PTSD), generalized anxiety disorder (GAD), and panic disorder are slightly later (Kessler et al. 2005). Giaconia found that the maximum crest risk time of PTSD was from 16 to 17 years in adolescent community samples, and about one-third of the samples developed the disease before the age of 14 years (Giaconia et al., 1994). In a national mental health survey questionnaire, Vaingankar followed 6,616 respondents and found that the average age of onset of GAD and obsessive-compulsive disorder (OCD) was 19 and 20, respectively (Vaingankar et al., 2012).

Another common mental health problem among college students is depression. The prevalence of college students is 7% to 9% (Blanco et al., 2008; Eisenberg et al., 2013). Zisook et al found that more than half of all cases of depression are first detected in childhood, adolescence or young adulthood (Zisook et al., 2007). Similarly, other studies also showed an increasing risk of mood disorders from early adolescence, and it continued to increase linearly with age. In the National Comorbidity Survey replication study, Kessler et al reported one in five people with depression have their first episode before the age of 25 (Kessler et al., 2005).

In short, the onset of anxiety and depression is highly consistent with the age of college students. Therefore, the mental health problems of college students can not be ignored, especially in the current epidemic period.

2.2.2 Decline in physical fitness among college students

In recent decades, it has become a fact that the number of college students has been observed to decrease (ACHA-NCHA, 2006; Sacheck et al., 2010). Regular

physical exercise plays the important role of a healthy lifestyle, and it is closely related to reducing the risk of cancer (Coyle, 2009), obesity (Shaw et al., 2006), and heart disease (Powell, 1988), and it is associated with lower levels of mental health-related stress (Brown, 1991) and better cognitive function (Etnier et al., 1997). Recent research showed that almost half of U. S. college students did not have moderate or vigorous PA (Douglas et al., 1997). Compared with high school students, college students' PA has dropped dramatically (Bray et al., 2004). According to self-reported weight and height, approximately 35% of U. S. college students are obese or overweight(Lowry et al., 2008).

Barnett analyzed the physical condition of African American college students and found that the transition from household to college life may take place changes in diet and exercise behavior. It is reported that about 12% to 50% of college students will suffer from one or more mental health disorders (Barnett et al., 2019). Nelson found that vigorous physical activity (VPA) showed a downward trend from adolescence to adulthood, and there were social differences in VPA in universities with insufficient PA in the university environment (male: 74% to 52%; female: 68% to 44%). Among them, women, African Americans, Asians, and students with lower socioeconomic status are less likely to participate in VPA during college. Therefore, the senior high school VPA was adjusted. Among men, Asians and older students are unlikely to participate in VPA, and universities are an important environment for promoting VPA and addressing health disparities (Nelson, et al., 2007).

Habitual high-intensity PA has been significantly reduced in the early and late stages (Halal et al., 2012), and the college period is considered to be in this early stage. Among college students from 23 high-income, middle-income, and low-income countries, the rate of physical inactivity was 41.4%, for example, it was 21.9% in Kyrgyzstan and 80.6% in Pakistan (Pengpid et al., 2015). Many U.S. college students fail to complete the recommendations for moderate or vigorous PA, or a combination of the two, and there was only 49.9% of U.S. college students who met the guidelines of ACHA (American College Health Association, 2017).

Bo believed that the factors affecting the physical health of college students

were not only due to the lack of public sports facilities, such as first-class stadiums and equipment, but also the universities and students' own factors were also important reasons for the PF decline of college students (BO, 2021). Mazurek studied the impact of lifestyle on the health of college students at a university in the United States. The results showed that the lifestyle of college students greatly affected their physical health (Mazurek et al., 2016). When Casado-Perez also studied the relationship between PF and the adaptability of college students by testing aerobic fitness, shoulder extensions, extended jumps and sprints, the results showed that the healthier the students' physical condition with the longer the exercise time (Casado-Perez et al., 2015). Barbara tested muscle strength, cardiopulmonary function and other physical function parameters of college students while studying the effects of their physical health on various physiological and pathological processes. The results showed that the health of college students was affected by pathological and physiological processes (Barbara et al., 2015).

With abundant social materials, people pay more and more attention to their physical health. However, so far, only about 10% of the Chinese population have good and advanced health concepts and literacy.

In China, the prevalence of students aged 9-22 with PA time <1 hour/day is high. The highest prevalence rate of PA time less than 1 hour/day was 82.5% of 21-year-old female students and 18-year-old male students (Wang et al., 2014). Physically inactive students are likely to be inactive in later life, which puts them at health risks. Therefore, college students should become the target group of physical exercise promotion, because this may prevent the current trend of rapid decline in PA after college students enter the adult stage (Keating et al., 2015; Wang, 2019).

Generally speaking, in recent years, domestic and foreign scholars have researched the development of college students' health from different perspectives, but they have not yet been able to fundamentally curb the decline in college students' health (Liu, 2020). The reason is that it has not comprehensively analyzed the influencing factors that affect the physical development of college students from multiple dimensions but only started from a certain aspect, which does not conform to

the overall development law. Based on these, this study will analyze and evaluate the PF and health status of college students through the analysis of PF test data and literature data. Through the intervention of TC, the relationship between physique and psychology is analyzed from the perspective of mental health, and reasonable suggestions are put forward based on the research content.

2. 2.3 Comorbidities, changes in cognition among college students

Although the intelligence of college students is at the highest level, multiple cross-sectional research showed fluid abilities were showing a steady decline trend from age 20 to 80 (Salthouse, 2010), see figure 2. So it is also necessary to prevent cognitive aging in a safe and inexpensive way for college students.

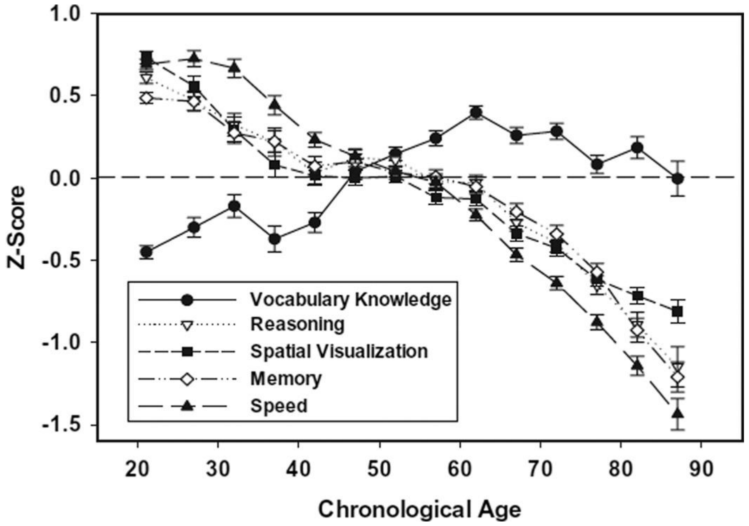

Figure 2 The changes of cognitive abilities with aging.

Note: The zero lines represent the average performance in study.

In addition, cognitive aging is one of the most common comorbidities of many chronic physical changes including PF, PA, sedentary lifestyle, and psychological problems. The impact of exercise, PA, and physical health on cognition is further based on well-established literature in the general population throughout the life cycle (ie, healthy children, young people, and the elderly), which proves that exercise, PA, and physical health beneficial effects on cognitive function and its underlying neural substrates (Voss et al., 2011). Therefore, if the mental health of college students will lead to cognitive bias, at the same time due to the decline of PF, abnormal body composition, may lead to obesity or overweight, and overweight will

further aggravate psychological distortion and cognitive decline.

It has also been reported that obese people may be mentally healthier (Crisp & McGuines, 1976), which is the so-called "happy fat" hypothesis (Han et al., 2009). However, most studies have shown that obesity may lead to poor mental health risks (Friedman and Brunel, 1995), and many studies have confirmed that obesity can lead to deterioration of quality of life, especially in the body And mental health (Atlantis et al., 2012), and some researchers claim that the impact of obesity on mental health is still unclear and needs to be further explored (Roberts et al., 2002). In addition, there are reports that compared with women, men are less likely to be negatively affected by mental health (Garner et al., 2012), and there is also evidence to support that the quality of life of obese men is higher than that of normal-weight men in adulthood (Costa-Font & Gil, 2006).

There is evidence that obesity is prospectively associated with low quality of life (Faith et al. 2011) but, at the same time, there are also studies that show a positive association between Body Mass Index(BMI) and psychological distress in women (Yu et al., 2011; Magallares & Pais-Ribeiro, 2013). In addition to physical consequences, obesity/overweight have connections with all kinds of mental consequences, such as low quality of life, body dissatisfaction, emotional problems, low self-esteem, eating disorders, depression, stress and so on.

In clinical samples of obese adolescents, compared with non-obese controls, the lifetime prevalence of anxiety is reported to be higher (Britz, 2000). In addition, one of the main findings in literature is that increased body composition is negatively correlated with cognitive ability under the frontal cortex. Ability, including executive function, complex attention, and psychomotor processing speed. This set of capabilities is related to the functions of the frontal cortex areas and the circulatory circuits that connect these areas to the subcortical structure and other cortical areas, such as the parietal lobe (Miller, 2007), and seem to preferentially affect obese individuals (Fergenbaum, 2009).

More specifically, the cognitive ability of obese people is significantly worse than people with normal weight. Cognitive deficits in obese children and

adolescents are mostly concentrated in tests of executive function (Lokken et al., 2009; Mond, 2007; Cesje'si, 2009; Verdejo-Garcia et al., 2007), and some studies also found defects in short-term memory (Li, 2008), as an aspect of executive function (Sunderland et al., 1986), overall function (Miller, 2006; Miller, 2009), and language ability (Azurmendi, 2005). Li found overweight children (BMI more than 95% of the recommended age and height) overall performed significantly worse in the finger span test that is a working memory and attention test (Li et al., 2008). However, there was no significant difference in general intelligence between obese individuals and normal controls (Lokken KL, 2010).

In addition, obesity also affects cognitive function in the following ways (Russell-Mayhew et al., 2012). For example, the results of the psychological performance task showed that the performance of obese individuals was significantly reduced (Etou, 1989). Several studies have provided evidence of an association between obesity and decreased visual structure (Boeka, 2008; Lokken, 2010).

Some studies provided evidence for the effects of obesity on visual and language memory (Gonzales, 2010; Lokken, 2010; Cournot, 2006; Gunstad, 2006), but the experimental results are inconsistent and contradictory, including obesity and inhibition (Fagundo, 2012; Ariza, 2012), Howieson and Loring's classification (Lezak, 2004).

Overall, more and more evidence shows that physical exercises such as aerobic and anaerobic exercises have been studied for cognitive improvements, such as TC and meditation (David, 2021). In some studies, attention span, language, and visual memory seem to increase temporarily. However, this effect is short-lived and will diminish over time and PA ceases.

2. 3 Definition, types, and mechanism of Tai Chi

2. 3.1 Definition of Tai Chi

In recent years, it has become more and more popular worldwide, not only for its martial arts but also for its soothing and beneficial aspects. TC is a relatively new martial art, which specifically originated in the 1820s, although it may have

existed for some time before then. This is the so-called soft fighting, which emphasizes the posture of relaxing the muscles and the use of the opponent's momentum, while the hardstyle emphasizes that the muscles are in a state of high alert to meet the needs of the opponent. In addition to the martial arts aspect, many concepts emphasize meditation calmness, and overall physical health. In fact, for many people living in the modern world, TC is not martial art, but a sport and respiratory system designed for therapy. Just as yoga in the West has broken away from its original intention, this particular discipline has also become completely different.

2.3.2 Types of Tai Chi

Tai Chi, as well known as Taiji or Tai Chi Quan/Chuan, is differently described as meditation originating from China or internal martial arts or physical and mental exercises. TC was developed by Chen Wangting in the late Ming Dynasty that is about 17th century AD (Gu & Shen, 2007). Chen Wangting fully integrates the essence of Chinese folk martial arts and military arts, meditation and breathing techniques, Taoist philosophy of yin and yang, and traditional Chinese medicine theory (Tang & Gu, 2012).

Today, TC is widely practiced in Eastern and Western countries for its health benefits. And there are many types of TC. Judging from the widely spread TC in front of the park, it can be divided into three types according to the size of the frame. Besides, there are 5 common types of TC, and each classic style has its form. Therefore, the actions and characteristics of different styles are different.

(1) Chen style TC. Mainly by Professor Chen Changxing from Chenjiagou, Henan province, of which Chen Fa is the most famous. It is characterized by strength and softness, speed and slowness. There are new boxes, old boxes, big boxes, and small boxes.

(2) Yang style TC. Yang Luchan studied boxing in Yang Changxing, then went to Beijing to teach boxing, and passed his son Yang Jianhou. Yang Jianhou passed on Yang Chengfu and succeeded Yang Chengfu for promotion. Its

characteristics are slow speed and stretch.

(3) Wu / Martial style TC. Wu Yugang first studied under Yang Luchan, and then from Chen Qingping in Zhaopu, Henan. His brother Wu Chengcheng received Wang Zongyue's "Book of Tai Chi" in Wuyang Yandian, Henan. Wu Yugang studied and improved deeply. It is characterized by flexible movements and flexible footwork.

(4) Sun-style TC. Sun Lutang improved Hao Weizhen's boxing skills. It is characterized by opening and closing drums, compact and compact, and flexible.

(5) Wu style TC. Wu Jianquan studied under his father Wu Quanyou (a disciple of Yang Luchan) and formed his system. It is characterized by softness, compactness, and moderate size.

After the People’s Republic of China was founded, to improve the health of the whole people, the Chinese government simplified these traditional forms of TC and developed a variety of competition forms on this basis to promote the promotion of five classical TC. In 1956, the State Sports General Administration published the book "Simplified TC" based on the classic Yang-style TC, which simplified the 81 -form to the currently most popular 24-form. Global style (Gu & Shen, 2007). In addition, TC can be practiced with partners in a more practical way, called "Push Hands" (two-person interactive practice). There are also variants of TC, which are practiced with weapons such as swords, knives, folding fans, wooden sticks, or spears.

In short, no matter how TC evolves, all styles and forms are directly or indirectly derived from the Chen style, their core principles and theories such as balance, breathing, coordination, relaxation, and concentration are similar.

2.3.3 The mechanism of tai chi

TC is becoming more and more popular body-mind exercise. It combines low-impact mobile movement with various cognitive skills related to attention deficit hyperactivity disorder (ADHD), including physical awareness, mental concentration, goal setting, task transfer, and visualization. It is considered a safe and low-cost complementary therapy that millions of people practice for various purposes.

(1) The structure of TC

Traditionally, the main components of TC are exercise, meditation and imagination, and deep breathing (Tang & Gu, 2012). This enables practitioners to obtain physical and psychological benefits from TC. TC movements consist of a series of slow, continuous, and graceful movements. The body is round and spiral called forms.

In the past ten years, clinical researchers have studied various components of TC. Wayne (Wayne et al., 2013) deconstructed multiple components of TC as follows table 1:

Table 1 Multiple components of Tai Chi

FEATURE	DESCRIPTION
Meditation	Develop awareness of the present moment during TC by paying attention to the position, movement and sensation of the body
Imagination	The image is used as a learning strategy (for example, one of the actions is called waving like a cloud)
Symmetrical structure	Exercise is biomechanical and requires minimal effort
Relaxation and flexibility	Circular and flowing movements provide dynamic stretching, helping to transform the body and mind into a deeper state of relaxation
Balance and strength	Putting self-weight on one foot at a time and bending slightly can increase the strength of the lower limbs and improve balance
Natural respiration	Rhythmic breathing exercises seem to improve breath exchange and promote calm
Community support	Positive interaction within the community creates a sense of support and belonging
Integration of spirit, mind, and body	TC creates a practical framework for a more comprehensive life

In clinical research, the type, form, training and practice methods, frequency, duration, and intensity of TC intervention may vary. Regardless of the specific style, form, or teaching and practice methods, TC itself is a complex intervention consisting of multiple parts, each of which has potential independent and collaborative therapeutic value (Wayne & Kaptchuk, 2008). Therefore, it is more appropriate to regard TC as a whole system study rather than a single component. Therefore, the intervention of TC in clinical research is usually provided as a full dinner for the participants. It usually consists of warm-up exercises, TC exercises, and cooling exercises for each training class.

(2)The potential central reaction of TC

As a typical body-mind exercise, TC not only needs to control physical movement, but also needs to cooperate with the state of physical and mental unity. It is recognized that aerobic exercise and meditation have positive effects on cognitive functions, including decision-making and memory skills (Deepeshwar, 2014; Moriarty, 2019).

Recent research has further confirmed the influence of TC exercises on the brain function and structure of practices (Silveira, 2019). For example, many MRI studies in elderly have shown that training of TC could cause significant changes in the brain volume (Adcock, 2019), brain white matter network (Yue, 2020), and spontaneous brain function activities (Zheng, 2015). These studies showed that TC exercises can affect both brain function and brain structure. These are the core mechanisms of TC non-specific and specific effects.

An fMRI study showed that significant differences existed in several brain regions related to motor control regions, somatic and sensory processing, and emotional process modulation in baseline brain activity (Zhu, 2016). At the same time, the use of fMRI research also found that the change in the functional connection between the cognitive control network and the resting state in both the medial prefrontal cortex and the anterior cingulate cortex may be the central mechanism of TC intervention in fibromyalgia (Jalilianhasanpour, 2019). Notably, fMRI is one of the most commonly used brain imaging techniques and has been widely used in the

study of the central mechanism of TC intervention (Kong, 2019; Jalilianhasanpour, 2019).

2.4 The effectiveness and benefits of Tai Chi on health

TC is a gentle and slow movement, combining cognitive tools and breathing techniques to relax, strengthen, and integrate the body and mind (Zou, 2018). It not only requires very little physical space, but also fits all ages people with different physical conditions (Kong, 2019). The benefits of TC are supported by many well-designed studies (Laird, 2018; Yeung, 2012; Yeung, 2017).

TC was first reported as an intervention in clinical research in 1958. This case series conducted in China investigated the beneficial effects of TC on tuberculosis (Wang, 1958). In 1988, the first TC RCT was conducted to study the role of TC combined with Qigong in cardiac rehabilitation (Sun et al., 1988). The intensity of TC is considered to be low to medium and can be easily adjusted to meet the requirements of different intensities (Gu & Shen, 2007). Therefore, this is a viable option for people who are sedentary or have limited exercise capacity (such as people with heart disease). In recent years, study on the safety mental and physical benefits of TC in healthy people and various patient groups has increased. By consulting literature, we can find that many countries in the world have conducted clinical studies of TC, covering various diseases/conditions, such as chronic obstructive pulmonary disease (COPD), coronary heart disease, Parkinson's disease, heart failure, osteoarthritis, stroke, depression, fibromyalgia, diabetes and hypertension (Huang et al. 2021; Yang et al., 2015).

2.4.1 The effects of tai chi on cognitive function

Research-based on brain science shows that in a new systematic review, 11 studies are included, of which 5 studies are RCTs and 5 studies are quasi-experimental designs, and all research designs are found to have medium to high-quality research designs. Pan and colleagues concluded that TC is beneficial to the elderly in terms of mind control, memory, health, cognition, and brain executive function. It is worth

noting that the results of brain neuroimaging studies prove the benefits of TC on the neuroplasticity of the brain of healthy elderly people. According to reports, these studies can delay or reverse the rate of neurodegeneration. Other neurological brain changes associated with TC exercises include increased cortical thickness associated with the intensity of TC exercises, better functional connections between brain regions, and increased spontaneous brain activity associated with TC-induced cognitive improvement (Pan et al., 2018).

Similarly, Xie pointed out that the metabolic output (3-6 METs) of TC is equivalent to brisk walking (medium aerobic intensity). The combination of this exercise component with meditation and mindful breathing can help prevent aging. The growth of cognitive ability declines. Xie and colleagues (2019) used brain function near-infrared spectroscopy (fNIRS) to find that long-term (>5 years) TC exercises enhanced the brain function of the occipital cortex, motor cortex, and prefrontal cortex, and improved sympathetic and parasympathetic nerves. Compared with matched controls during exercise and rest, the system regulates, improves the brain blood supply and enhances brain connectivity (Xie et al., 2019).

Liu and colleagues used magnetic resonance imaging (MRI) and behavioral measurements to prove that long-term practice of TC (>10 years of practice) showed neurology, that is, greater brain GMV, which matches the nerves compared with the control group, plasticity, and emotional stability and behavioral benefits, namely higher levels of meditation, higher emotional stability and less risk-taking propensity. The conclusion is that these results support the role of TC in reducing brain atrophy and promoting emotional stability in the elderly (Liu et al., 2019). Cui and colleagues found that compared with the matched control group, TC and aerobic exercise significantly increased regional GMV and functional connectivity, indicating that TC and aerobic exercise affect memory recovery, attention, and self-recovery (Cui et al., 2019).

In addition, several studies have shown that TC intervention may have positive effects, such as improving or maintaining cognitive function and reducing the risk of dementia in non-disabled elderly people (Lam, 2011; Wayne, 2014). A

systematic review comparing TC with conventional physical activities in maintaining the cognitive ability of healthy adults shows that TC is more effective than pure physical exercise in maintaining overall cognitive skills. Some emerging studies have demonstrated the potential effectiveness of TC in improving cognitive function in the early stages of dementia for a short term. Burgener conducted a 40-week intervention on dementia patients to evaluate the effectiveness of various interventions and finally concluded that an intervention combining TC, participation and cognitive therapy can effectively improve or maintain the cognitive function of dementia patients (Burger, 2008). Wayne conducted a systematic review to explore the effectiveness of TC on the cognitive abilities of adults with and without cognitive impairments, and concluded that TC can enhance the cognitive abilities of adults without obvious obstacles and the executive function of the elderly (Wayne, 2014). However, Lim also pointed out that Wayne’s experimental sample includes participants with early to late dementia, and this heterogeneous group may mask the influence of any subgroup of participants (Lim et al., 2019).

At present of EEG field, there are the following outcomes for different types of brain wave power on EEG during or after meditative exercise, for example, EEG recordings showed higher beta power was noted along with higher alpha power, which is a pattern noted during attentiveness (Liu, Y. et al., 2003; Field, T., 2011). Similar to other studies also observed increased power in theta and alpha power during meditation (Wayne, P. M. et al., 2014). Furthermore, TC as a meditative exercise, Li, X. found that the TC group had significantly higher alpha power in the post-test than in the pre-test, and control groups had no significant differences, and there was a significant difference in the alpha power between the two groups in the post-test, with those of the TC group being the highest (Li, X. et al., 2020), Similar to other studies, Nakatani, Y. also found the alpha power augmentation on EEG during and after TC exercise, which evoked a gradual cerebral blood flow increase in the prefrontal cortex and a gradual cerebral blood flow decrease in the parietal cortex, and that TC activated the prefrontal cortex (Nakatani, Y. et al., 2006).

2.4.2 The effects of tai chi on physical fitness

There are many clinical studies about health conditions and specific diseases, but their quality and methods are inconsistent, so it is difficult to summarize clear conclusions (Yang, 2015). According to reports, TC can be used to treat a variety of human diseases and is supported by many associations, including the Australian Diabetes Association and the National Parkinson's Foundation. But there is a lack of effective medical evidence, and research has been conducted in recent years to solve this problem (Lee, 2010; Baggoley, 2015). A systematic review in 2017 found that it reduces the risk of falls in the elderly (Lomas-Vega, 2017).

A comprehensive overview of the systematic evaluation of TC in 2011 recommends TC to the elderly because of its various physical and psychological benefits (Lee, 2010). A systematic review found that people with osteoarthritis, COPD, and heart failure can perform TC without aggravating pain and shortness of breath, and found that people with osteoarthritis, COPD, and heart failure can perform TC without aggravating pain and shortness of breath. The functional athletic ability of the person is favorable (Chen et al., 2015). The Australian Government’s Department of Health published the results of an alternative therapy review in 2015 to determine whether any alternative therapy is suitable for inclusion in health insurance; TC is one of the 17 therapies evaluated and concluded that it is associated with a limited number of people's effects. Compared with the limited number of results in the exercise, TC may have some beneficial effects on health (Baggoley, 2015). Specifically, TC requires mental concentration, concentration, mind circulation, internal and external triad (inner triad refers to the combination of mind, qi, and power, that is, the combination of mind and qi, and the combination of qi and strength.; Sanhe refers to hands and feet, elbows and knees, shoulders and hips). These subtle, complex, and unique exercises and requirements are integrated into the practice of TC, which is a good exercise for the brain (Zheng, 2009).

This combination of movement and static is beneficial to the regulation of cerebral cortex excitement and inhibition. It has a significant effect on neurasthenia, insomnia, and dizziness caused by excessive excitement of the cerebral cortex. If

persisted for a long time, it can gradually eliminate the pathological excitement of the cerebral cortex caused by the disease, to achieve the therapeutic effect.

TC emphasizes relaxation of the whole body. It requires not only body relaxation but also brain relaxation. Under the control of the brain, the nerves and muscles relax, which can reflexively relax the small arteries of the whole body (hypertension is mainly manifested as small arteries) and relieve the hardening of the small arterial walls. In this way, blood pressure drops and tends to be normal, which is more beneficial to hypertensive patients. After mental and physical labor, the whole body relaxes, so that excited nerves and tired muscles can recover faster. This is why practicing boxing can eliminate fatigue better than static. In addition, the movement of the limbs can not only exercise the elasticity of the muscles but also increase the speed of blood circulation, thereby preventing and treating cardiovascular and cerebrovascular diseases caused by obstruction of blood flow.

In the concept of the cerebral cortex, on the other hand, the nervous system keeps the whole body in a static state. Deep breathing promotes the relaxation and contraction of internal and external organs and muscles. The spiral wounds of the waist, spine, and limbs will accumulate (abdominal) air. As for the whole body, peripheral nerves can produce feelings of acid, numbness, swelling, and heat, commonly known as "qi liver". People with this sense of movement of qi and blood have ruddy skin and their body temperature can rise by about 1 degree (Zheng, 2009). Through the movement of qi, about 200 capillaries per square millimeter of muscle are opened for use (under normal circumstances, only about 5 have blood flow). And the capillaries open and close according to a certain cycle. Therefore, their throbbing seems to add millions of tiny "hearts" to the body. These small peripheral hearts develop in large quantities, reduce the burden on the heart, and are of great benefit to the prevention and treatment of heart disease.

TC exercises gradually deepen the breathing, so the diaphragm drops more. Stir the diaphragm up and down to strengthen the chest and abdomen movement, and play a "massage" effect on the viscera. This is an effect that drugs cannot achieve. In this way, the blood flow of the chest and abdomen organs is strong, and the absorption

function is strengthened. It has a good effect on internal organs diseases such as gastrointestinal indigestion, diabetes, and urinary incontinence. Deep breathing in TC makes the lungs expel a lot of turbid air, inhale more oxygen, improve the efficiency of lung ventilation, and enhance lung tissue elasticity. This can reduce the ossification rate of costal cartilage, enhance the mobility of the thorax, and have a certain effect on the prevention and treatment of lung diseases and emphysema.

2.4.3 The effects of tai chi on mental health

TC is widely regarded as a feasible activity to improve the functional ability and health of people with various health conditions such as the elderly, patients with chronic diseases, and children with intellectual disabilities (Zou et al., 2018). The characteristics of TC emphasize the slow and dynamic weight transfer (requires strong lower limbs), combined with physical awareness, breathing control, and mental concentration (Zou, 2017). Many studies have shown that TC exercises can effectively improve PF (Nguyen, 2012; Kong et al. 2019). In the past two decades, TC training which purely emphasizes health has become more popular in elderly centers, communities, clinics, and hospitals.

TC is a PA that also incorporates meditation, social, and cognitive elements. It is becoming more and more popular and aroused a lot of interest (Wayne, 2014). It involves the orchestration of learning actions, which requires episodic memory, visuospatial skills and rapid information processing. In addition, TC also speeds up heart rate and breathing, which helps to build a larger network of connections between neurons, thereby enhancing brain perfusion. Previous studies have also shown that TC is a safe and effective activity that can improve the physical balance and emotional health of non-disabled elderly people (Del-Pino-Casado, 2016; Wang, 2010).

TC has been proven to be beneficial both physically and psychologically (Wang, 2004). This suggests that TC can help reduce depression and improve the sustainability of positive effects of metabolic syndrome and depression, which has important implications for the prevention and management of cardiovascular disease. In addition, three recent reviews suggest that TC may have some positive effects on

the mental health of patients with depression (Wang, 2014; Chi, 2010; Wang, 2010). In addition, TC can reduce the severity of anxiety, stress, depression and leg strength in central obese patients with depressive symptoms (Liu et al. 2015).

2.5 Summary

Under the COVID-19 epidemic since 2019, young people, especially college students, have further reduced the willingness to go out and exercise (Xiang, 2020; Shen, 2021), in addition, changes in lifestyle and behavior, increased sedentary or lying-down time (Palermi, 2020) or decreased willingness to exercise can lead to changes in psychologically, physically and Comorbidity aspects.

TC as a good intervention, non-pharmacological intervention for maintaining and improving cognitive function, mental health, and PF, has been confirmed by various experiments. However, the comprehensive effect of TC intervention needs to be further proved in college students.

CHAPTER III

Research Methodology

3.1 Study design overview

The design of this study will be a RCT with two parallel groups with a 1:1 allocation ratio, allocation concealment and assessor blinding. The design follows the Consolidated Standards of Reporting Trials (CONSORT) and the Standard Protocol Items Recommendations for Interventional Trials (SPIRIT).

Eligible participants will be collected according to the requirements of the experimental design. According to the cluster sampling method, a two-arm, parallel controlled trial, randomized was conducted. The participants who will receive primary prevention education of cognition, mental health, and physical fitness will be divided into two groups: the 12-week 24 Form TC exercise intervention group (45 min per session, three sessions per week) or control group.

Cognitive performance, including executive function, memory, and processing speed, will be assessed by using SCWT at baseline prior intervention 2-3 days and within 2-3 days after the 12-week intervention. In addition, before and after the 12th TC training session, all participants in the TC exercise group will be only assessed cognitive performance by using SCWT and HRV test.

Furthermore, both the function and structure of brain regions related to cognitive performance will be measured by using rs-EEG, and autonomic nerves in the nervous system will be measured by using HRV, and fitness health factors (power of lower limbs, body mass index, body composition, cardiopulmonary function, muscle strength, balance, flexibility) will be measured at baseline prior intervention 2-3 day and within 2-3 days after the 12-week intervention. In addition, mental health will also be assessed by using Depression Status Inventory and State-Trait Anxiety Inventory Version at baseline prior intervention 2-3 days and within 2-3 days after the 12-week intervention.

All data collection of the group assignments will be blinded. The flow diagram

for this study is presented in figure 3.

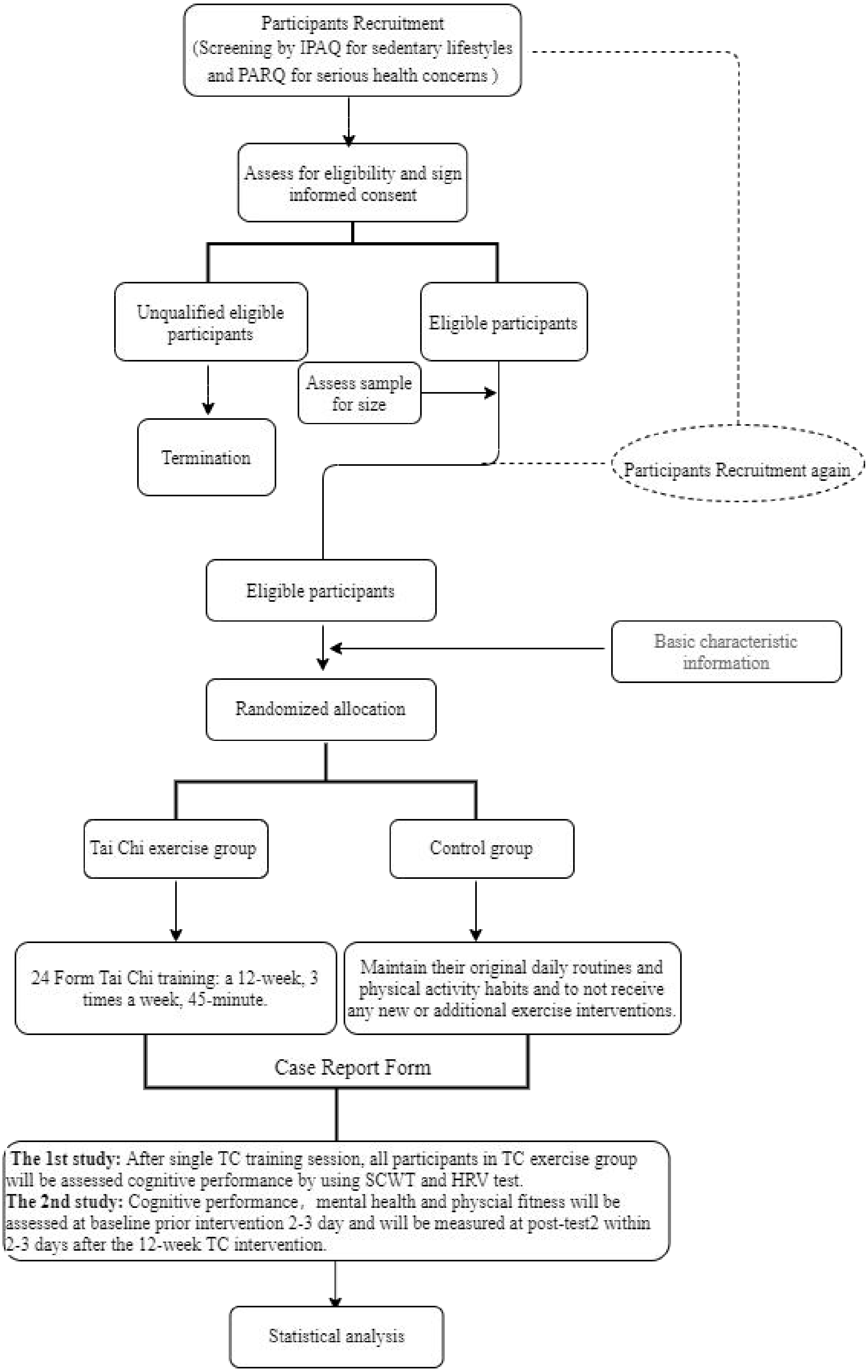

Figure 3 Flow diagram of the study design.

3.2 Recruitment and sample

3.2.1 Recruitment

Study recruitment will be conducted on the campus of Huzhou University. Preliminary recruitment information and requirements will be posted on both the job flyer and university social pages, and interested potential participants can contact the principal investigator by calling the phone number provided or via email in the job flyer. In the first week before the formal experiment, the principal investigator will arrange a meeting for about an hour was arranged in the Sports Anatomy and Physiology Laboratory at 12 o'clock every day to clarify and explain the specific experimental process, and select eligible volunteers, which include taking PARQ and IPAQ questionnaires, and Control the inclusion and exclusion criteria, select eligible subjects and sign the informed consent form.

In addition, this study will use Stratified Blocked Randomization. An independent statistician will first stratify eligible subjects according to "gender" and "BMI ranges that are underweight (under 18.5 kg/m2), normal weight (18.5 to 25kg/m2), overweight (25 to 30kg/m2), and obese (over 30kg/m2)", block followed by randomization to TC or control group, ensuring that each subject will be randomly assigned to either intervention or control and there is a balance between the number and the BMI value of subjects in both groups. Finally, eligible volunteers will be randomly assigned to the TC group and the control group by envelope sampling. Of course, during any of the above processes or times, the volunteer can ask questions about the research project, and the researcher will further explain and answer the questions until the volunteer understands. Volunteers can still withdraw from the study at any time without affecting their rights during volunteers participating in this study.

3.2.2 Sample size, screening, and allocation

(1) Sample size estimation

Depending on the type of data or study design, there are multiple methods that can be used to calculate the sample size. If suppose that the sample sizes of the

two groups in this design are equal, and compare the means of the two groups, use the following formula to calculate the sample size equation:

$$n = (Zcrit+Zpow)^2 *2*\sigma^2 / d^2 \quad \text{or}$$

$$n = (Zcrit+Zpow)^2 * (SD_1+SD_2)^2 / (M_1 — M_2)^2$$

Here, n represents the sample size of each group; assuming that the two groups of samples are the same, σ^2 represents the variance of either group; D represents the smallest detectable value between the two sample means. Zcrit and Zpow respectively represent the significance level and the standard normal deviation of 1-β power. The standard normal deviation is the realization of the standard normal random variable， For the two-sided test, the Zcrit value is 1.96 and the significance level is 5%. The Zpow is 0.84 at 80% power and 1.28 at 90% power (Wang & Ji, 2020).

To determine the sample size (Lu et al., 2016), data from our previous prospective study on college students show that TC training achieved significant improvement in cognitive performance (TC group: 0.392 ± 0.087; Inactive Control group: 0.475±0.097) after the seven-week intervention when compared with subjects in the control group (Converse, 2020). Our estimation of sample size was based on the independent Compare 2 Means analysis between the two groups. Calculations were performed with WinPepi software (Abramson, 2011), one of a set of computer programs for epidemiologists. An overall sample size of 40 participants (20 participants per group) achieves an 80% power at the 0.05 significance level. Taking into account the drop-out rate for each group, we expect that a total sample size is 50 participants (25 participants per group).

(2)The screening criteria

Inclusion criteria required that participants: 1) ages between 18 and 22 years, 2) could be independently ambulant, 3) were able to participate in a low-moderate intensity gentle movement group for 12 weeks, 4) attend classes on the Huzhou

University, 5) Normal vision or corrected vision, no color blindness, 6) being nonsmoker, not drinking alcohol, 7) not being physically active regularly (more than once per month), 8) not taking antioxidant supplementation, 9) no use of medication regularly, 10) absence of musculoskeletal pain or condition that limits exercise practice, and 11) the commitment of attending the TC sessions, and a self-reported sedentary lifestyle (i.e., not engaging in PA for the last 6 months).

Exclusion criteria included participants who: 1) participated in any type of professional sports, 2) reported any history of cardiopulmonary disease, 3) were able to participate in a low-moderate intensity gentle movement group for 12 weeks, 4) are pregnancies or suspect to be pregnant, 5) Take any prescription drugs (except oral contraceptives), take acute infections, illegal drugs, pregnancy, and vast physical exercise during the previous 2 weeks, 6) experienced in applying cognitive tasks or any limitations in the ability to complete cognitive testing procedures, 7) are with and without previous meditative movement and/or meditation practice, and 8) not allowed to consume any caffeinated or alcoholic products during the days of their study participation.

Potential participants will first complete a screening questionnaire, including International Physical Activity Questionnaire (IPAQ) and Physical Activity Readiness Questionnaire (PARQ), to determine their eligibility. IPAQ will be used to screen all participants for sedentary lifestyles (Tremblay et al., 2017), PARQ will be used to screen all participants for any serious health concerns that would preclude their involvement in PA (American College of Sports Medicine, 2018).

Eligible participants will receive information about this study and will have a discussion with researchers about the information provided. After signing the written informed consent, those interested in participating will arrange a baseline assessment of the results.

(3)Allocation

Participants will be first recruited from the Huzhou University campus by blinded research assistants. We expect the possible recruitment rate to be 60%–70%.

If the number of participants is not enough, we will recruit the remaining participants from the campus of Huzhou University by setting up recruitment stations, sending flyers, posting posters, and university social pages. Volunteers can consult their family or primary doctor before making a decision. Volunteers can also ask questions about the research project and the researcher will further explain and answer the question until volunteers understand it. If volunteers voluntarily participate in this study, volunteers can still withdraw from the study at any time without any impact on their rights.

In addition, this study involved multiple test items. The principal investigator will invite 3 assistants from the School of Physical Education of Huzhou University, who are teachers with more than 5 years of experience in exercise science experiments, to assist in the testing and evaluation of this study. To avoid bias and ensure effective data collection, Reliability will be assessed by calculating the intraclass correlation coefficient (ICC) with 95% confidence intervals. ICC can be used with quantitative data organized into groups, and it measures the consistency of values across cases. Comparing the results of the assistant, if the ICC value is greater than 0.9, it means that the reliability is good, and the results can be used.

Assistants will review the consent form with participants; upon signing, participants began data collection procedures. All data collection will occur in private offices on the Huzhou University campus. Participants will attend consent signing and data collection at individual times to prevent overlap and ensure participant privacy. Trained assistants will conduct data collection which will take place between March 2022 and July 2022.

3.3 Experiment content and test sequence

3.3.1 Experiment content

Participants will be asked to completely relax and sit comfortably in two quiet and bright rooms with an ambient temperature set at 24°C, located in the Sports Anatomy and Physiology Laboratory and the Laboratory of Cognitive Neuroscience and Learning Sciences of Huzhou University. All tests will be performed in the Sports Anatomy and Physiology Laboratory, except for rs-EEG tests in the Cognitive

Neuroscience and Learning Sciences Laboratory.

The Experiment contents of this study will be expected to include four parts, as follows:

(1)Mental Health questionnaires. This study will include Depression Status Inventory (DSI; Zung 1965) and State-Trait Anxiety Inventory Version (STAI; Spielberger et al., 1983).

(2) Cognitive Performance measurement. This study will use Stroop color word test (Eprime 2.0 Professional, Psychology SoftwareTools, Inc., Pittsburgh, PA). SCWT is widely applied to measure cognitive function by researchers and clinicians (MacLeod, 1991, 2015; Swanson, 2005). Stroop designed a classic test experiment, in which using different colors of ink to print color names in 1935. For example, if you see the word "red" printed with blue ink and say "blue", this will cause cognition interference (MacLeod, 1991). The Stroop effect appears when the individual's response time is prolonged due to the targeted selection of attention or the individual cannot inhibit the brain's first response to autonomous reading (MacLeod, 2015). The theoretical explanation of the Stroop effect includes automatic and processing speed (MacLeod, 1991). The relative speed of processing shows that humans can read written words faster than recognize colors, and that speed conflicts can cause interference (MacLeod, 2015). Automaticity is the automatic process that takes place quickly, unconsciously and effortlessly (Sahinoglu & Dogan, 2016). Barnett believed that because the name of the color conflicts with the process of automatically reading words, more attention may be required when recognizing the color of a word (Barnett et al., 2020).

(3) Physical Fitness measurement. This study will include PF test and body scale (Huawei Smart Scale 3 Pro, Huawei, China). The indexes of PF are including: Height and Weight (Weight scale, Jiangsu Suhong Medical Equipment Co., Ltd., China), Cardiopulmonary function (JH-1662 Electronic Spirometer, Jiangsu Suhong Medical Equipment Co., Ltd., China), Muscle strength (JH-1881 Electronic Grip , Changzhou Jihao Electronics Co., Ltd., China), Flexibility (Mechanical flexionr, Jiangsu Suhong Medical Equipment Co., Ltd., China), Balance (One-leg standing test

with eyes closed), Power of lower limbs (Simple long jump mat,, Jiangsu Suhong Medical Equipment Co., Ltd., China). The indexes of body composition include: Body water, Protein level, Body fat, Muscle mass, Bone mineral content (Huawei Smart Scale 3 Pro, Huawei, China).

(4) Physiological data acquisition. This study will include HRV measurement (uBioMacpa v70, BioSense Creative, Korea) and rsEEG measurement (actiCHamp Plus, Brain Products GmbH, Gilching, Germany).

3.3.2 Test sequence

Following informed consent.

In the 1st study, all participants in TC exercise group will be assessed before and after a 45-minutes TC training session in 4th week to determine the effects of single TC training session. Both pre-test2 and post-test1 will be in the following order: 1)SCWT, and 2) HRV test.

In the 2nd study, all participants in both TC exercise group and control group will be assessed before and after 12-weeks to evaluate the long-term effects of TC training session. Both pre-test1 and post-test2 will be in the following order: 1)DSI and STAI, 2) SCWT, 3)HRV test, 4)PF test and Body Scale, and 5)rs-EEG.

3.4 Experimental apparatus, procedure, and measure assessment

3.4.1 Depression Status Inventory

The state of depression can be measured by Depression Status Inventory (DSI; Zung 1965), DSI consists of 20 items, and each item has four answers, in which it is assigned 1 to 4 points according to the time level. Total scores of all items range from 20 to 80, and lower scores represent a "lower state of depression" (Kim et al., 2004). Compared to the clinical judgment of a psychiatrist, the reliability of the DSI was 0.90, the specificity of the DSI was 0.80, and the sensitivity of the DSI was 0.90 (Alber et al., 1992).

DSI is a short-term evaluation scale and questionnaire for others. It is convenient to operate and easy to grasp. It can effectively reflect the symptoms of

depression and their severity and changes. It is especially suitable for general hospitals to find depression patients. If the subject has a low level of education or intelligence and cannot perform self-assessment, DIS can be used to evaluate the examiner. DIS has been widely used abroad. Mainland of China was translated into Chinese in 1985 and was first used to evaluate the efficacy of the antidepressant minaPrine in the treatment of depression and clinical research on depression.

Scoring method: Each item is scored on four levels of 1, 2, 3, and 4. There are a total of 20 items, which reflect the four groups of specific symptoms of depression: Psychic-emotional symptoms, including two items of depression and crying; Physical disorders: eight items including day-to-day differences in mood, sleep disorders, constipation, appetite loss, libido loss, fatigue, weight loss and tachycardia; Psychomotor disorder, including psychomotor retardation and agitation; Psychological disorders of depression, including confusion, hopelessness, irritability, indecision, self-devaluation, feeling of emptiness, suicidal thinking and dissatisfaction, a total of eight items. In these 20 items, 10 items are set in positive words, which are scored in reverse order, and the remaining 10 items are negative words that should be scored in the order of 1-4 above. The scores range from 25 to 100, normal scores range from 25 to 49, Mildly Depressed scores range from 50 to 59, Moderately Depressed scores range from 60 to 69, and Severely Depressed scores are 70 and above.

3.4.2 State-Trait Anxiety Inventory

This study will use State-Trait Anxiety Inventory Version (STAI; Spielberger et al., 1983) to analyze the anxiety characteristics of college students. The first edition (STAL-Form X) came out in 1970 and has conducted 2,000 studies involving medicine, education, psychology and other sciences. The author revised STAI-Form X in 1979 and the 1980 revision (STAI-Form Y) began to apply. The former describes unpleasant emotional experiences, such as fear, tension, nervousness, and anxiety, accompanied by hyperactivity of the autonomic nervous system, but this is usually short-lived. Trait anxiety is used to describe a relatively stable anxiety

tendency, and this tendency is a unique trait of each person. Spielberg compiled the STAI, which provides internal scientists, behaviorists, and clinicians with a tool to distinguish between transient emotional states of anxiety and personality trait anxiety tendencies for clinical practice services and different research purposes.

STAI consists of 40 descriptive questions in the guide and two subscales. Items 1-20 are the State Anxiety Inventory (STAI, Form YI, hereinafter referred to as S-AD. Half of the items describe negative emotions, and the others are positive emotions. Mainly used to evaluate immediate or present specific experiences, or feelings in situations such as tension, worry, fear, and neuroticism. It can be used to evaluate state anxiety under stress conditions. The 21-40 topic is the Trait Anxiety Inventory (STAI, Form Yl, T-AI), which is used to assess individual frequent emotional experiences. Among them, 11 items are items describing negative emotions and 9 items are positive emotion items. It can be widely used to assess the anxiety of internal medicine, surgery, psychosomatic diseases and mental patients; it can also be used for screening. Anxiety issues of college students, military personnel, and other professional groups; and evaluation of the effects of psychotherapy and medication. The questionnaire can be used for individual or group testing, and the subjects generally only need a junior high school education level. Subjects can circle the answers based on self-assessment or self-report. There is no time limit for the entire questionnaire process. The scale includes item content explicitly assessing worry and bothersome thoughts, as well as physical problems such as restlessness, suggesting that it is a strong overall measure of GAD severity. Internal consistencies across the three study waves were 0.887, 0. 912, and 0. 917 (Newman et al., 2020).

The reliability of STAI tested among college students is 0.850. In addition, the construct validity of the STAI, the correlation between items (KMO) score is 0.824 (>0.30) and p=0.000. Where, the State showed KMO=0.818, p=0.000, and Cronbach alpha found 0.797; Trait showed KMO=0.783, p=0.000, and Cronbach alpha found 0.781 (Vitasari et al., 2011).

Generally speaking, the entire questionnaire can be completed in about 15 minutes. Each item of STAI is scored from grade 1 to grade 4. In S-AI, 1 means not at

all, 2 means some, 3 means moderate, and 4 means very obvious. In T-AI, 1 means almost none, 2 means some, 3 means often, 4 means almost always. The subjects choose the most suitable score based on their own experience. All positive emotion items are scored in reverse order. Calculate the cumulative scores of the S-AI and T-AI scales, with a minimum of 20 and a maximum of 80, reflecting the degree of state or trait anxiety (Hui, 2020).

3.4.3 Stroop color word test

The SCWT consisted of two sessions: word recognition (WR); color recognition (CR), and used software Eprime 2.0 Professional (Psychology SoftwareTools, Inc., Pittsburgh, PA), which allows online recording and voice-key triggering of the participants' verbal responses, for presenting the stimuli and collecting the data.

In order to further reduce the non-specific interfering factors of the Stroop task, such as practice times，stimulus encoding, contextual factors, color name facility, operational tasks, the amount of SOA, the subject’s age and mood, the proportion of words and non-words（Rezaei, M.，2019； Edwards, S. et al., 1996）, The stimuli were four-color word names presented in Chinese word as 红(RED), 黄(YELLOW), 蓝(BLUE) and 绿(GREEN). SCWT will produce two types of situations. In the case of the same word color, the meaning of the word matches the color ink. For example, the word BLUE is presented in blue ink; when the word color is inconsistent, the meaning of the word conflicts with the color of the ink. , Such as the word BLUE in red ink (Zhu, 2020). Participants will be asked to choose any a key (f, j, r, u) based on the word presented in four colors: red, yellow, blue, or green. The four colors used the left index finger, the right index finger, the left middle finger, and the right middle finger, respectively. For example, if the word color is yellow, whatever the word, the correct response is to press the "j" key with the right index finger. The stimulus consisted of randomly matched colors and words (120 incongruent trials, 40 congruent trials). All stimuli were presented for 500 ms followed by fixation point (+) for 250ms and a

stimulus remained displayed on the screen for a maximal duration of 1000 ms, see figure 4. The conflict effect of the two groups before and after inhibitory control training was evaluated through response time and error rate in both the congruent and incongruent stimulus (Xu et al., 2021). All subjects are required to practice until the correct rate is higher than 60% before the formal test can be conducted.

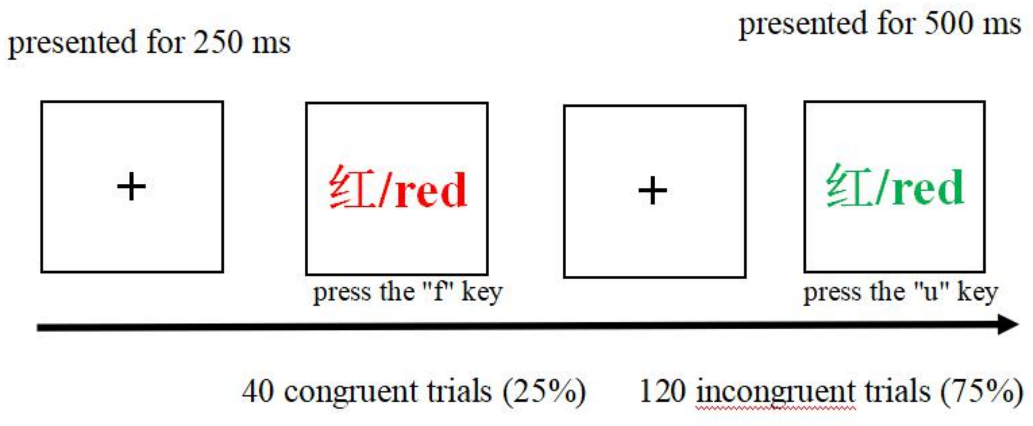

Figure 4 SCWT process

3.4.4 HRV measurement

In HRV measurement, uBioMacpa v70 (BioSense Creative, Korea) analyzes heart pulses by measuring pulse waves noninvasively on fingertip capillaries. It can check the vascular age and the degree of stress in the human body, and also can check whether the autonomic nervous system is abnormal. For example, uBioMacpa was used to measure autonomic stress levels.

The vascular health will be measured by deducing the vascular age from the age of the patient. Considering that there can be a difference in the measurement results, the heart rate variability of the autonomic nervous system will be measured 2.5 minutes when the subject is sitting down in quiet, see figure 5. The high-frequency values (HF), low-frequency values (LF), low-frequency ratio, mean heart rate per minute values (Mean BMP), standard deviation between pulses (SDNN), and the root mean square values of the standard deviation of pulses will be collected from HRV indicators. A pulse is a wave that produces when the blood which is pumped from the heart touches the blood vessel wall, which is repeated by the

heartbeat due to the changes of the blood flow. It is measured through the signal detected by applying light of a specific wavelength to the human body. The uBioMacpa stress measurement is based on the standards provided by the analysis of the average pulse variability signals from the North American Heart Rate Electrophysiology Society and the European Heart Association (Vanderlei et al., 2009).

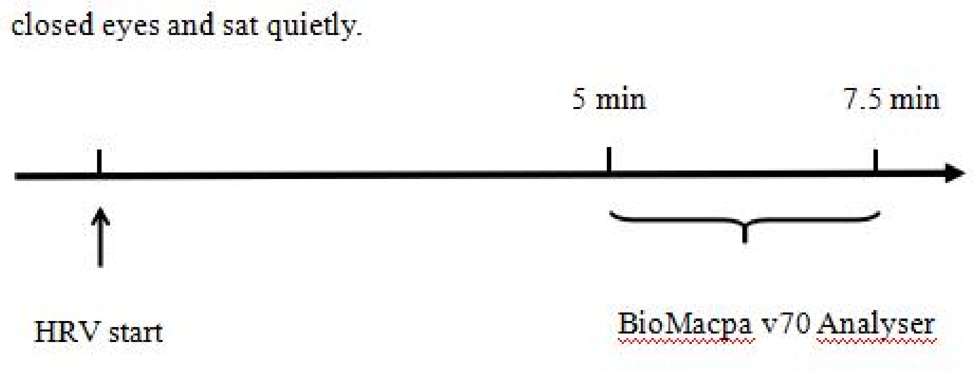

Figure 5 HRV measurement process

3.4.5 Physical Fitness test

The test indexes, procedures and sequence are as follows:

(1)Height and weight. Using Height and Weight scale (Weight scale, Jiangsu Suhong Medical Equipment Co., Ltd., China), it reflects the human development and nutritional status and the level of longitudinal growth of human bone marrow. During the Height test, the participant will stand on the floor of the height meter with bare feet and standing upright (the torso is straight, the upper limbs droop naturally, the heels are close together, the toes are separated by about 60°C, and the sacrum, heel, and both shoulder blades are in contact with the height meter column , The head is straight, the eyes are looking straight ahead, the upper edge of the tragus and the lowest point of the lower edge of the orbit are level. The record is in centimeters, with one decimal place. Use a height meter to test, the accuracy is 0.1cm. During the weight test, the participant naturally stood in the center of the scale and read the data after standing firm. Record the kilogram as a unit, and keep 1 decimal place. Using a weight scale test, the accuracy is 0.1kg. In addition, during the

test, the participants should reduce their clothing as much as possible; when getting on and off the scales, they should act lightly.

(2)Cardiopulmonary function. Using JH-1662 Electronic Spirometer, it reflects the volume and expansion capacity of human lungs. The accuracy of the JH-1662 Electronic Spirometer is 1ml, the accuracy of the flip spirometer is 20ml, and the accuracy of the barrel spirometer is 50ml. During the test, the participants inhale deeply until they can no longer inhale, and then point their mouths to the mouth of the spirometer and exhale deeply until they are exhausted. Test twice and take the maximum value. Records are in milliliters, rounded up. In addition, do not exhale too hard to prevent leakage; do not inhale twice; the mouth and mouth of the spirometer should be strictly disinfected.

(3)Muscle strength. JH-1881 Electronic Grip is used to test the grip strength, which reflects that the muscle strength of the human forearm and hand is a common functional indicator for evaluating the level of human growth and physical fitness. During the test, the participant turns the grip distance adjustment knob of the grip meter to adjust the grip distance to the appropriate grip distance, and then hold the grip meter firmly. Based on previous literature (Konharn et al., 2018), it is recommended that males should take a 1-minute standing rest posture when measuring grip strength, and young adult women should take a 1-minute sitting rest posture. Grip the handles with maximum force. Test twice and take the maximum value. The record is in kilograms, with one decimal place. In addition, it is forbidden to swing arms, squat, or touch the dynamometer to the body when exerting force: if the participant cannot distinguish a strong hand, test both hands twice.

(4)Flexibility. The mechanical flexionr is used for the sitting body flexion test, which reflects the flexibility of the human body. During the test, the participant sits on the mat, with legs straight, heels together, toes separated naturally, and the soles of the feet are pushed on the tester plate; then the palms are down, the arms are stretched flat, the upper body is bent forward, and the middle fingers of both hands are used. Push your fingertips to move forward until you can't push it. Test twice and take the maximum value. Records are in centimeters, with one decimal place. In

addition, before the test, the participant should do preparatory activities to prevent muscle strain. During the test, the knee joint must not be flexed, and there must be no sudden forward vibration. The positive and negative signs should be correctly filled in when recording.

(5)Balance. Using a one-leg standing test with eyes closed, it reflects the body's ability to balance. During the test, the participant stands naturally, and when he hears the "start" command, he lifts any foot while the tester starts the watch to time the test. When the tester’s supporting foot moves or lifts the foot, the tester stops the watch. Take the test twice to get good results. The record is in seconds, with two decimal places. When testing, protect the surrounding area of the tester. The reliability of the SLS test is reported to be 0.89 and 0.86 when eyes are open and closed, respectively (Birmingham, 2000), and the Dynamic One Leg Stance was a fairly valid and reliable balance test for from 19 to 61 years participants with vision loss, acquired and experimental (Blomqvist & Rehn,, 2007).

(6) Power of lower limbs. Use a simple long jump mat for standing long jump test, which reflects the strength of the lower limbs of the human body. During the test, the participant stands with his feet naturally separated behind the jumper, and both feet simultaneously take off forward at the same time. The vertical distance between the heel and the jumper will be used as the test score. Each person jumped three times. Take centimeters as the unit, keep one whole number after the decimal point, and take the best score among the three test scores as the final score. In addition, before the test, the participant should do the necessary warm-up and stretching activities, and wait in an orderly manner at the test point. In the test, the participant has a stepping action when taking off, stepping on the line, crossing the line, the take-off score is invalid.

3.4.6 Body composition

Body composition will be measured by body scale (Huawei Smart Scale 3 Pro, Huawei, China), and it has a professional eight-electrode and high and low frequency dual-frequency technology to comprehensively measure fluid data inside

and outside the cell. The principle is based on the HUAWEI TruFitTM human body composition model, which was developed by Huawei and the Hefei Institute of Material Science, Chinese Academy of Sciences. It uses bioelectrical impedance readings, large-scale data verification and artificial intelligence technology, and uses both low-frequency and high-frequency currents to calculate the results (Huawei, 2021). The accuracy of the results of this instrument is comparable to the professional gold standard and fat mass. The correlation between the skeletal muscles of the limbs and the DEXA professional human body analyzer can reach 0.94 (Laka, P., et al., 2021). The measurable range of weight is 2.5kg-150kg, which can provide accurate data of various body types from 6 to 80 years old. The indexes of body scale include: body water, body fat, protein level, muscle mass, bone mineral content (Huawei, 2021).

During the test, each subject will undergo a BIA performed using a Huawei body scale. All subjects will be measured in an assumed normally hydrated state and have not eaten or participated in physical activity a minimum of 90 mins prior to being measured. Huawei Smart Body Fat Scale 3 Pro adopts optical coating process, with ITO conductive coating and no explicit electrode. At the same time, Huawei Smart Body Fat Scale 3 Pro adopts an embedded handle design. Subjects only need to pull out the handle and touch the eight electrodes on the scale surface and handle respectively. With Huawei body composition detection algorithm, more accurate health data can be obtained. Before using it, you need to download the Huawei Sports Health App and pair it with a mobile phone running Android 6.0 or iOS 9.0 or above. Once paired, the subjects simply stepped barefoot on the scale, then pulled out the handle and clasped their hands for about 15 seconds to get a detailed report.

3.4.7 rs-EEG measurement

rs-EEG will be recorded by using the ActiCHamp amplifier with 64-channel active AgCl electrodes (actiCHamp Plus, Brain Products GmbH, Gilching, Germany), and it will be positioned on the scalp according to the modified 10–20 system.

Previous literature showed that Tai Chi can definitely increase alpha power

during or after TC exercise (Li, X. et al., 2020; Nakatani, Y. et al., 2006), and maybe along with higher beta power and theta power (Liu, Y. et al., 2003; Wayne, P. M. et al., 2014). In order to measure these more accurately, Electrode gel will be inserted into the midfrontal (F3 & F4), central (C3 & C4), anterior temporal (T3 & T4), and parietal (P3 & P4) sites and referenced to the vertex (Cz) during recording (Field, T. et al., 2010). The horizontal electroophthalmogram will record a bipolar configuration between electrodes located on the lateral sides of each eye canthus; and vertical eye movements will be monitored by a bipolar montage located between electrodes above and below the right eye. Recording will be performed using an on-line 50Hz notch filter. After recording, the data will be restored to a rate of 500Hz and the total mean will be re-referenced (Barnett, K. J., 2008). It will apply both off-line filters with a high cut-off frequency of 0.1Hz and a low cut-off frequency of 35Hz. EEG preprocessing will be performed using BrainVisionAnalyzer software (BrainVisionAnalyzer, Brain Products GmbH, Gilching, Germany).

The whole process lasts eight minutes, EEG will be selected and recorded for three minutes (Gasser et al., 1996), see figure 6. Power spectrum will be computed during the HRV test. Spectral power analysis will be performed at four-second intervals by first applying the Welch window to the data and executing FFT. If the signal from three or more sites for that particular period exceeds 100μV, the period will be rejected. The power spectrum will be averaged for each electrode and calculated for each band: alpha (8 -- 13Hz), beta (14.5 -- 30Hz), delta (0.5 -- 3.5Hz), and theta (4 -- 7.5Hz).

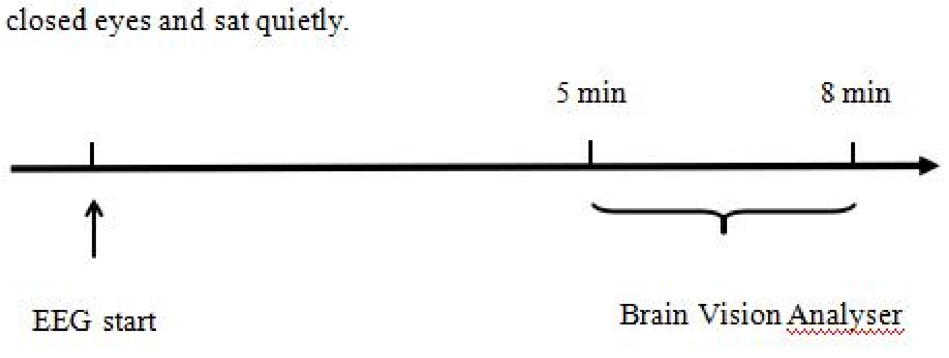

Figure 6 rs-EEG measurement process

3.5 Exercise intervention and control procedures

3.5.1 Exercise intervention

A review of previous meta-analysis shows that long-term exercise has a positive effect on cognitive function (Sibley & Etnier, 2003), evidence shows compared with 12 participants with general aerobic exercise, eight weeks of one-hour TC training, three times a week, had a stronger effect on brain plasticity (Cui et al., 2019). However, review literature shows that no effects on the MMSE were found following TC after 8 weeks, 24 weeks, or 24 months (Chang et al., 2014), and some findings suggest that TC had beneficial effects on cognitive function, but a longer duration was not associated with larger effects in older adults (Chen et al., 2021). Based on the primary results, looking at a previous RCT of aerobic exercise for cognition in (Lam, 2012; Hung et al., 2013; Xu, 2020), twelve weeks has also been suggested as a duration for interventions (Brown & Summerbell, 2009; Liu, 2018). Based on the above, the duration of this trial is determined to adopt a 12-week, 3 times a week, 45-minute RCT design.

TC exercise group received three weekly sessions of 24 Form TC group training for 12 weeks. Each training session consisted of 5 min of warm-up, 35 min of continuous sequential practice of learned forms, and 5 min of cool-down. Affected by epidemic control, two group training sessions will be held every week. The systematic approach will be taught by two instructors and can be viewed in Figure 7 (Zheng, et al., 2014), and another TC training session will be held in groups or completed by students themselves.

Figure 7. 24 Form Tai Chi

3.5.2 Exercise Intensity Monitoring

Tai Chi had a lower metabolic demand and it is considered aerobic exercise at a low intensity. Lan, C. et al. showed the heart rate during TC practice was 58% of the heart rate reserve (Lan, C. et al., 2008) or 58% of the heart rate range (Lan, C. et al., 2001). In another study, the heart rate was also 56% of the age-predicted heart rate maximum, and Gong et al. reported that the HR in participants aged 48 to 80 was 104

bpm during the practice of simplified TC (Gong et al., 1981). The average heart rate of the TC group will be controlled at about 100 bpm during the 12-week exercise intervention. A bracelet will monitor the average heart rate of the TC group.

TC session instructor will be a physical educator with a Master's degree or above in sport science, and will have rich experience in TC teaching. The whole program will have two teaching assistants, and the student-teacher ratio across the program administration will be 15 to 1.

3.5.3 Control group

The control group will be instructed to maintain their original daily routines and physical activity habits and to not receive any new or additional exercise interventions.

3.5.4 Safety evaluation

Any adverse events, including any functional injury caused by the intervention, such as lumbar muscle strain, knee pain, and knee or ankle sprain, will be recorded on the case report form (CRF) during the intervention. If there is any adverse event, the investigator will respond accordingly based on the on-site situation. At the same time, adverse events will be reported to the ethics committee immediately to determine whether participants need to withdraw from the trial.

3.6 Statistical analysis

The sample's demographic characteristics, descriptive data, age, education, race, and ethnicity will be described using the mean and standard deviation or frequencies and proportions as appropriate.

In the 1st study, based on the results of the pre-test2 and post-test1 in only TC exercise group, both Paired t-test and McNemar test will be used to explore differences in the outcomes that are response time and error ratio in SCWT, and LF, HF, LF/HF, Mean BPM, SDNN and RMSSD in HRV test.

In the 2nd study, there will also be three levels of outcomes in both TC exercise

group and control group. The primary outcomes are response time and error ratio in SCWT; LF, HF, LF/HF, Mean BPM, SDNN and RMSSD in HRV test; Average power in EEG. The secondary outcomes are body fat, BMI, body fat ratio in body composition; and vital capacity, balance, handgrip strength, vertical jump, and sit and reach flexibility in PF. And the tertiary outcomes are depression and anxiety on psychological scales. Two-way repeated measures ANOVA will be performed with 2 levels in the group factor (Group: TC, Control) and 2 levels in the time factor (Time: pre-test1, post-test2) to assess three levels of outcomes. All analyses will be conducted with IBM SPSS 26.0, with the statistical significance level set at p≤0. 05.

Reference

Abramson JH (2011) WINPEPI updated: computer programs for epidemiologists, and their teaching potential. Epidemiologic Perspectives & Innovations 2011, 8:1 accessories/scale-3-pro/Accessed 7 September 2021

Albert, M. S., Levkoff, S. E., et al.,. (1992). The delirium symptom interview: an interview for the detection of delirium symptoms in hospitalized patients. Topics in geriatrics, 5(1), 14-21.

Alexandr, F., & Vadim, E. (2016). Health behavioral factors in modern adolescents. Journal of Physical Education and Sport, 16(1), 109.

American College Health Association. (2006). American college health association national college health assessment (ACHA-NCHA) spring 2005 reference group data report (abridged). Journal of American College Health, 55(1), 5.

American College Health Association. American College Health Association-National College Health Assessment II: Reference Group Undergraduate Executive Summary Spring 2017. Hanover, MD: American College Health Association; 2017. 18.

American Psychiatric Association (2013). Diagnostic and Statistical Manual of Mental Disorders (Fifth ed.). Arlington, VA: American Psychiatric Publishing. p. 189. ISBN 978-0-89042-555-8.

Ariza, M., Garolera, M., Jurado, M. A., et al. (2012). Dopamine genes (DRD2/ANKK1-TaqA1 and DRD4-7R) and executive function: their interaction with obesity. PLoS One, 7(7), e41482.

Arnett JJ. Emerging adulthood. (2000). A theory of development from the late teens through the twenties. Am Psychol, 55(5): 469-80.

Atlantis, E., Goldney, R., Eckert, K., & Taylor, A. (2012). Trends in health-related quality of life and health service use associated with body mass index and comorbid major depression in South Australia, 1998–2008. Quality of Life Research, 21, 1695–1704.

Azurmendi, Aitziber, et al. (2005). Cognitive abilities, androgen levels, and body mass index in 5-year-old children. Hormones and Behavior, 48(2), 187-195.

Baggoley, C. (2015). Review of the Australian Government Rebate on Natural Therapies for Private Health Insurance PDF. Australian Government – Department of Health. Lay summary – Gavura, S. Australian review finds no benefit to 17 natural therapies. ScienceBased Medicine.

Barnett, K. J. (2008). The effects of a poor night sleep on mood, cognitive, autonomic and electrophysiological measures. Journal of Integrative Neuroscience, 7(03), 405-420.

Barnett, M., Sawyer, J., & Moore, J. (2020). An experimental investigation of the impact of rapport on Stroop test performance. Applied Neuropsychology: Adult, 1-5.

Barnett, T. M., McFarland, A., Miller, J. W., Lowe, V., & Hatcher, S. S. (2019). Physical and Mental Health Experiences among African American College Students. Social Work in Public Health, 34(2), 145-157.

Birmingham, T. B. (2000). Test–retest reliability of lower extremity functional instability measures. Clinical Journal of Sport Medicine, 10(4), 264-268.

Blanco, C., Okuda, M., Wright, C., et al. (2008). Mental health of college students and their non–college-attending peers: results from the national epidemiologic study on alcohol and related conditions. Archives of general psychiatry, 65(12), 1429-1437.

Blomqvist, S., & Rehn, B. (2007). Validity and reliability of the Dynamic One Leg Stance (DOLS) in people with vision loss. Advances in Physiotherapy, 9(3), 129-135.

Bo, P. (2021). Analysis and intervention on the influencing factors of college students' physical fitness. Revista Brasileira de Medicina do Esporte, 27, 11-13.

Boeka, A. G., & Lokken, K. L. (2008). Neuropsychological performance of a clinical sample of extremely obese individuals. Archives of Clinical Neuropsychology, 23(4), 467-474.

Bouras N, Holt G (2007). Psychiatric and Behavioral Disorders in Intellectual and Developmental Disabilities (2nd ed.). Cambridge University Press.ISBN 9781139461306.

Bray, S. R., & Born, H. A. (2004). Transition to university and vigorous physical

activity: Implications for health and psychological well-being. Journal of American College Health, 52(4), 181-188.

Britannica [online] 2021 Mar, [cited 2021 Mar 2].. Available from:

Brown, J. D. (1991). Staying fit and staying well: Physical fitness as a moderator of life stress. Journal of personality and Social Psychology, 60(4), 555.

Brown, Tamara, & Summerbell, Carolyn. (2009). Systematic review of school-based interventions that focus on changing dietary intake and physical activity levels to prevent childhood obesity: an update to the obesity guidance produced by the National Institute for Health and Clinical Excellence. Obesity Reviews, 10(1), 110-141.

Burgener, S. C., Yang, Y., Gilbert, R., & Marsh-Yant, S. (2008). The effects of a multimodal intervention on outcomes of persons with early-stage dementia. American Journal of Alzheimer's Disease & Other Dementias®, 23(4), 382-394.

C. Wang, R. Bannuru, J. Ramel, B. Kupelnick, T. Scott, and C. H. Schmid.(2010), “Tai Chi on psychological well-being: systematic review and meta-analysis,” BMC Complementary and Alternative Medicine, vol. 10, article 23.

C. Yue, L. (2020). Zou, J. Mei et al., “Tai Chi training evokes significant changes in brain white matter network in older women,” Healthcare, vol., 8, no. 1, p. 57.

Casado-Pérez, C., Hernández-Barrera, V., Jiménez-García, R., et al. (2015). Time trends in leisure time physical activity and physical fitness in the elderly: Five-year follow-up of the Spanish National Health Survey (2006–2011). Maturitas, 80(4), 391-398.

Chacko, S. A., Wee, C. C., Davis, R. B., & Yeh, G. a. Y. (2014). Use of Meditation and Breathing Exercises in Mind-Body Exercise in the U.S. The Journal of Alternative and Complementary Medicine, 20(5), A121–A121.

Chang, Y. K., Nien, Y. H., Chen, A. G., & Yan, J. (2014). Tai Ji Quan, the brain, and cognition in older adults. Journal of Sport and Health Science, 3(1), 36-42.

Chawla, N., Tom, A., Sen, M. S., & Sagar, R. (2021). Psychological Impact of COVID-19 on Children and Adolescents: A Systematic Review. Indian Journal of Psychological Medicine, 43(4), 294.

Chen, M. L., Wotiz, S. B., Banks, S. M., Connors, S. A., & Shi, Y. (2021). Dose-Response Association of Tai Chi and Cognition among Community-Dwelling Older Adults: A Systematic Review and Meta-Analysis. International journal of environmental research and public health, 18(6), 3179.

Chen, X., ling Ke, Z., Chen, Y., & Lin, X. (2021). The prevalence of sleep problems among children in mainland China: a meta-analysis and systemic-analysis. Sleep Medicine.

Chen, Y. W., Road, J. D., Camp, P., & Reid, W. D. (2015). The Pain Experience And Comorbities That Cause Pain In Pulmonary Rehabilitation Participants With Chronic Obstructive Pulmonary Disease (COPD). In D35. IT'S ALL TOO MUCH: COPD COMORBIDITIES (pp. A5727-A5727). American Thoracic Society.

Cognition. Lexico. Oxford University Press and Dictionary.com. Retrieved 6 May 2020.

Converse, A. K., Barrett, B. P., Chewning, B. A., & Wayne, P. M. (2020). Tai Chi training for attention deficit hyperactivity disorder: A feasibility trial in college students. Complementary Therapies in Medicine, 53, 102538.

Costa-Font, J., & Gil, J. (2006). Revisiting the ‘Fat and Jolly’ hypothesis: socio-environmental determinants of obesity and depression in Spain. Socio-Economic Review, 4, 513–542.

Cournot, M. C. M. J., Marquie, J. C., et al. (2006). Relation between body mass index and cognitive function in healthy middle-aged men and women. Neurology, 67(7), 1208-1214.

Cox WT, Abramson LY, Devine PG, Hollon SD (2012). "Stereotypes, Prejudice, and Depression: The Integrated Perspective". Perspectives on Psychological Science. 7 (5): 427–49.

Coyle, Y. M. (2009). Lifestyle, genes, and cancer. Cancer Epidemiology, 25-56.

Crisp, A. H., & McGuiness, B. (1976). Jolly fat: relation between obesity and psychoneurosis in general population. British Medicine Journal, 1, 7–9.

Cserjési, R., Molnár, D., et al. (2007). Is there any relationship between obesity and mental flexibility in children?. Appetite, 49(3), 675-678.

Cui, L., Yin, H., Lyu, S., Shen, Q., Wang, Y., Li, X., & Zhu, L. (2019). tai chi chuan vs General Aerobic exercise in Brain plasticity: A Multimodal MRi Study. Scientific reports, 9(1), 1-7.

Cui, L., Yin, H.,et al. (2019). Tai Chi Chuan vs general aerobic exercise in brain plasticity: a multimodal MRI study. Scientific reports, 9(1), 1-7.

Daniel M. Landers.(2010) "The Influence of Exercise on Mental Health". President's Council on Physical Fitness and Sports. Retrieved February 5,2010.

David Asensio. (2021), Available from: https://www.cognifit.com/cognition

Davison GC (2008). Abnormal Psychology. Toronto: Veronica Visentin. p. 154.ISBN 978-0-470-84072-6.

Del-Pino-Casado R, Obrero-Gaitan E, Lomas-Vega R.(2016). The effect of Tai Chi on reducing the risk of falling: a systematic review and meta-analysis. Am J Chin Med. 44(5): 895-906.

Douglas, K. A., Collins, J. L., et al. (1997). Results from the 1995 national college health risk behavior survey. Journal of American college health, 46(2), 55-67.

Edwards, S., Brice, C., Craig, C., & Penri-Jones, R. (1996). Effects of caffeine, practice, and mode of presentation on Stroop task performance. Pharmacology Biochemistry and Behavior, 54(2), 309-315.

Eisenberg D, Hunt J, Speer N. Mental health in American colleges and universities: Variation across student subgroups and across campuses. J Nerv Ment Dis. 2013;201(1): 60-7.

Elkins, G., Fisher, W., & Johnson, A. (2010). Mind–Body Therapies in Integrative Oncology. Current Treatment Options in Oncology, 11(3), 128–140.

Etnier, J. L., Salazar, W., Landers, D. M., Petruzzello, S. J., Han, M., & Nowell, P. (1997). The influence of physical fitness and exercise upon cognitive functioning: A meta-analysis. Journal of sport and Exercise Psychology, 19(3), 249-277.

Etou, H., Sakata, T., et al. (1989). Characteristics of psychomotor performance and time cognition in moderately obese patients. Physiology & behavior, 45(5), 985-988.

F. Wang, E. K. Lee, T. Wu et al..(2014), "The effects of tai chi on depression,

anxiety, and psychological well-being: a systematic review and meta-analysis," International Journal of Behavioral Medicine, 21(4), 605–617.

Fagundo AB, de la Torre R, Jimenez-Murcia S, Aguera Z, Granero R, Tarrega S, et al. (2012). Executive functions profile in extreme eating/weight conditions: from anorexia nervosa to obesity. PLoS ONE, 7 (8): e43382

Fergenbaum JH, Bruce S, Lou W, Hanley AJ, Greenwood C, Young TK. (2009) Obesity and lowered cognitive performance in a Canadian first nation population. Obesity 17,1967-1963.

Field, T. (2011). Tai Chi research review. Complementary Therapies in Clinical Practice, 17(3), 141-146.

Field, T., Diego, M., & Hernandez-Reif, M. (2010). Tai chi/yoga effects on anxiety, heartrate, EEG and math computations. Complementary therapies in clinical practice, 16(4), 235-238.

Fogelholm, M., & van Marken Lichtenbelt, W. (1997). Comparison of body composition methods: a literature analysis. European Journal of Clinical Nutrition, 51(8), 495-503.

Friedman, M., & Brownell, K. (1995). Psychological correlates of obesity: moving to the next research generation. Psychological Bulletin, 117, 3–20.

Gallagher R, Gill AM, Sysko HB. National survey of counseling directors, 2000. Alexandria: International Association of Counseling Centers, Inc; 2000.

Gao F, Jiao GF and Dong D (2018). "Brain reward dysfunction and exercise therapy: evidence from functional imaging." Chinese Journal of Sports Medicine 37(05): 432-439.

Gao Y. (2020). Effects of social support and psychological capital on mental health of adolescents and college students under severe epidemic conditions (Master's thesis, Southwest University).

Garner, R., Feeny, D., Thompson, A., Bernier, J., McFarland, B., Huguet, N., et al. (2012). Bodyweight, gender, and quality of life: a population-based longitudinal study. Quality of Life Research, 21, 813–825. doi: 10.1007/s11136-011-9989-1.

Gasser T, Molinari L, The analysis of the EEG, Statistical Methods in Medical

Research 5:67–99, 1996

Giaconia R et al.(1999). Ages of onset of psychiatric disorders in a community population of older adolescents. J Am Acad Child Adoles Psychiatry, 33(5): 706-17.

Gilbert, Paul (2007). Psychotherapy and counselling for depression(3rd ed.). Los Angeles: SAGE.ISBN 9781849203494.OCLC 436076587.

Gong, L. S., Qian, J. A., Zhang, J. S., Yang, Q., Jiang, J., Tao, Q., & Zhang, X. M. (1981). Changes in heart rate and electrocardiogram during taijiquan exercise: analysis by telemetry in 100 subjects. Chinese medical journal, 94(9), 589-592.

Gonzales MM, Tarumi T, Miles SC, Tanaka H, Shah F, Haley AP. (2010).Insulin sensitivity as a mediator of the relationship between BMI and working memory-related brain activation. Obesity, 18 (11): 2131-2137.

Greene, J., Cohen, D., Siskowski, C., & Toyinbo, P. (2017). The relationship between family caregiving and the mental health of emerging young adult caregivers. The Journal of Behavioral Health Services & Research, 44(4), 551-5663.

Groot GC, Fagerstrom L (2011). "Older adults' motivating factors and barriers to exercise to prevent falls". Scandinavian Journal of Occupational Therapy. 18 (2): 153–160.

Gu, L. & Shen, J. (2007). Chen style Tai Chi. Beijing: People’s Sports Publishing House of China.

Gunstad J, Paul RH, Cohen RA, Tate DF, Gordon E. (2006). Obesity is associated with memory deficits in young and middle-aged adults. Eat Weight Disord ,11 (1): e15-9.

Guthold R, Stevens G A, Riley L M, et al.(2018).Worldwide trends in insufficient physical activity from 2001 to 2016: a pooled analysis of 358 population-based surveys with 1.9 million participants[J]. Lancet Glob Health,6(10): e1077-e1086.

Guthold R, Stevens G A, Riley L M, et al.(2020).Global trends in insufficient physical activity among adolescents: a pooled analysis of 298 population-based surveys with 1.6 million participants[J]. Lancet Child Adolesc Health,4(1): 23-35.

Hallal PC, Andersen LB, Bull FC, et al. (2012). Global physical activity levels:

surveillance progress, pitfalls, and prospects. Lancet, 380:247–257.

Halper, B., Hofmann, M., Oesen, S., Franzke, B., Stuparits, P., Vidotto, C., ... & Wessner, B. (2015). Influence of age and physical fitness on miRna-21, TGF-ß and its receptors in leukocytes of healthy women. Exercise immunology review, 21(21):154-163.

Han, C., Jo, S. A., Seo, J. A., Kim, B. G., Kim, N. H., Jo, I., et al. (2009). Adiposity parameters and cognitive function in the elderly: application of "Jolly Fat" hypothesis to cognition. Archives of Gerontology and Geriatrics, 49, 133–138.

Harper, C. (2014). Diagnostic and Statistical Manual of Mental Disorders, Fifth Edition (DSM-5). American Psychiatric Association. 2013.

Harris, P. E., Cooper, K. L., Relton, C., & Thomas, K. J. (2012). Prevalence of complementary and alternative medicine (CAM) use by the general population: a systematic review and update. International Journal of Clinical Practice, 66(10), 924–939.

Healy GN, Clark BK, Winkler EA, Gardiner PA, Brown WJ, Matthews CE. (2011). Measurement of adults' sedentary time in population-based studies. Am J Prev Med, 41(2): 216-227.

Hossen, M. S., Karmakar, P., Das, A., Rahman, A., & Chakma, M. (2020). Association of Sport and Exercise with Health Condition and Academic Performance: A Cross Sectional Study Among University Students in Bangladesh. Spor Hekimliği Dergisi, 55(4), 290-299.

Huang, J., D. Wang and J. Wang. (2021). "Clinical Evidence of Tai Chi Exercise Prescriptions: A Systematic Review." Evid Based Complement Alternat Med 2021: 5558805.

Huawei (2021). HUAWEI smart scale 3 Pro.https://consumer.huawei.com/en/

Hui, J. (2020). Correlation between sports activities and anxiety states of college students. Revista Argentina de Clinica Psychologica, 29(2), 301.

Hung, H. C., Chang, Y. J., Lai, R. S., & Chen, T. C. C. (2013). Effects of Community-Based Tai Chi Exercise on Health-Related Physical Fitness and Cognitive Functions in the Elderly. Journal of Nursing & Healthcare Research, 9(4).

I. Chi, M. Jordan-Marsh, M. Guo, B. Xie, and Z. Bai.(2013), "Tai chi and reduction of depressive symptoms for older adults: a meta-analysis of randomized trials," Geriatrics and Gerontology International, vol. 13, no. 1, pp. 3–12.

Idiegbeyan-ose, J.; Opeke, R.; Aregbesola, A.; Owolabi, S.; Eyiolorunshe, T.A. (2019).Relationship between Motivation and Job Satisfaction of Staff in Private University Libraries, Nigeria. Acad. Strateg. Manag. J, 18, 1–13.

Ives-Deliperi, V. L., Solms, M., & Meintjes, E. M. (2011). The neural substrates of mindfulness: an fMRI investigation. Social Neuroscience, 6(3), 231-242.

Jang, E. J., Yun, S. Y., Choi, B. J., & Kim, Y. G. (2019). The Horticultural Activity of Touching Soil and the Autonomic Nervous Stress Response of Patients with Brain Disease. Horticultural Science and Technology Journal, 37(1), 151-158.

Jimenez-Pavon, D., Carbonell-Baeza, A., & Lavie, C. J. (2020). Physical exercise as therapy to fight against the mental and physical consequences of COVID-19 quarantine: Special focus in older people. Progress in cardiovascular diseases, 63(3), 386.

Keating, X. D., Guan, J., Pinero, J. C., & Bridges, D. M. (2005). A meta-analysis of college students' physical activity behaviors. Journal of American college health, 54(2), 116-126.

Kelly ME, Loughrey D, Lawlor BA, Robertson IH, Walsh C, Brennan S. (2014).The impact of exercise on the cognitive functioning of healthy older adults: a systematic review and meta-analysis. Ageing Res Rev,16:12-31.

Kessler, R. C., Amminger, G. P., Aguilar-Gaxiola, S., Alonso, J., Lee, S., & Ustun, T. B. (2007). Age of onset of mental disorders: a review of recent literature. Current opinion in psychiatry, 20(4), 359.

Kessler, R. C., Angermeyer, M., et al. (2007). Lifetime prevalence and age-of-onset distributions of mental disorders in the World Health Organization's World Mental Health Survey Initiative. World psychiatry, 6(3), 168.

Kessler, R. C., Berglund, P., et al. (2005). Lifetime prevalence and age-of-onset distributions of DSM-IV disorders in the National Comorbidity Survey Replication. Archives of general psychiatry, 62(6), 593-602.

Kim, K. B., Cohen, S. M., Oh, H. K., & Sok, S. R. (2004). The effects of meridian exercise on anxiety, depression, and self-esteem of female college students in Korea. Holistic Nursing Practice, 18(5), 230-234.

Kong J, Wilson G, Park J, et al.(2019). Treating depression with Tai Chi: state of the art and future perspectives[J]. Frontiers in psychiatry, 10: 237.

Kong, J., Wolcott, E., et al. (2019). Altered resting state functional connectivity of the cognitive control network in fibromyalgia and the modulation effect of mind-body intervention. Brain imaging and behavior, 13(2), 482-492.

Kong, Z., Sze, T. M., et al. (2019). Tai chi as an alternative exercise to improve physical fitness for children and adolescents with intellectual disability. International journal of environmental research and public health, 16(7), 1152.

Konharn, K., Chaichan, T., Leungbootnak, A., Karawa, J., & Udomtaku, K. (2018). Interval rest period and different-testing positions on hand-grip strength measurement among young adults. Journal of Medical Technology and Physical Therapy, 30(3), 267-276.

Korczak, D.J.; Madigan, S.; Colasanto, M. (2017).Children's Physical Activity and Depression: A Meta-Analysis.Pediatrics, 139.

Köteles, F., & Simor, P. (2014). Somatic symptoms and holistic thinking as major dimensions behind modern health worries. International Journal of Behavioral Medicine, 21(5), 869-876.

Kurth, F., Cherbuin, N., and Luders, E. (2015). Reduced age-related degeneration of the hippocampal subiculum in long-term meditators. Psychiatry Res. 232, 214–218.

Kuy, S., Tsai, R.,et al. (2020). Focusing on vulnerable populations during COVID-19. Academic Medicine.

Kwan, M. Y., Cairney, J., et al. (2012). Physical activity and other health-risk behaviors during the transition into early adulthood: A longitudinal cohort study. American Journal of Preventive Medicine, 42(1), 14–20.

Laird KT, Paholpak P, Roman M, Rahi B, Lavretsky H.(2018). Mind–body therapies for late-life mental and cognitive health. Curr Psychiatry Rep 20(1): 2.

Laka, P., Korzeb, Z., & Mazurczyk, W. (2021). Novel user authentication method

based on body composition analysis. Annals of Telecommunications, 76(3), 175-185.

Lam, L. C., Chau, R. C., Wong, B. M., et al. (2011). Interim follow-up of a randomized controlled trial comparing Chinese style mind body (Tai Chi) and stretching exercises on cognitive function in subjects at risk of progressive cognitive decline. Int. J. Geriatr. Psychiatry 26, 733–740.

Lam, L. C., Chau, R. C., Wong, B. M., Fung, A. W., Tam, C. W., Leung, G. T.,... & Chan, W. M. (2012). A 1-year randomized controlled trial comparing mind body exercise (Tai Chi) with stretching and toning exercise on cognitive function in older Chinese adults at risk of cognitive decline. Journal of the American Medical Directors Association, 13(6), 568-e15.

Lan, C., Chen, S. Y., & Lai, J. S. (2008). The exercise intensity of tai chi chuan. Tai Chi Chuan, 52, 12-19.

Lan, C., Chen, S. Y., Lai, J. S., & Wong, M. K. (2001). Heart rate responses and oxygen consumption during Tai Chi Chuan practice. The American journal of Chinese medicine, 29(03n04), 403-410.

Lee, C. M., Cadigan, J. M., & Rhew, I. C. (2020). Increases in loneliness among young adults during the COVID-19 pandemic and association with increases in mental health problems. Journal of Adolescent Health, 67(5), 714-717.

Lee, I. M., Shiroma, E. J., et al. (2019). Association of step volume and intensity with all-cause mortality in older women. JAMA internal medicine, 179(8), 1105-1112.

Lee, L. Y., Lee, D. T., & Woo, J. (2010). The psychosocial effect of Tai Chi on nursing home residents. Journal of Clinical Nursing, 19(7-8), 927-938.

Lehert, P., Villaseca, P., Hogervorst, E., Maki, P. M., & Henderson, V. W. (2015). Individually modifiable risk factors to ameliorate cognitive aging: a systematic review and meta-analysis. Climacteric, 18(5), 678-689.

Lezak, M. D., Howieson, D. B., Loring, D. W., & Fischer, J. S. (2004). Neuropsychological assessment. Oxford University Press, USA.

Li SW, Wang Y, Yang YY, Lei XM & Yang YF.(2020). Factors influencing anxiety disorder in children and adolescents isolated at home during COVID-19 epidemic. Chinese Journal of Child Health Care (04), 407-410.

Li Y, Dai Q, Jackson JC, Zhang J. (2008). Overweight is associated with decreased cognitive functioning among school-age children and adolescents. Obesity ,16: 1809–1815.

Li, X., Yin, H., Cui, L., et al. (2020). Tai Chi Improves Alpha Brain State: An EEG Power Spectra And Standardized Low-resolution Tomography Analysis. The Journal of the International Society of Chinese Health Practices, 1(1).

Li, Y. (2019). Research on the Relationship between Physical Fitness, Executive Function and Default Network of College Students. Master's Thesis, Yangzhou University, Yangzhou, China.

Liang, I. J., Perkin, O. J., McGuigan, P. M., Thompson, D., & Western, M. J. (2021). Feasibility and Acceptability of Home-Based Exercise Snacking and Tai Chi Snacking Delivered Remotely to Self-Isolating Older Adults During COVID-19. Journal of Aging and Physical Activity, 1(aop), 1-11.

Lim, K. H., A. Pysklywec, M. Plante and L. Demers. (2019). "The effectiveness of Tai Chi for short-term cognitive function improvement in the early stages of dementia in the elderly: a systematic literature review." Clin Interv Aging 14: 827-839

Liu Lu.(2020). Emotional Problems and Educational Counseling Strategies of Higher Vocational Students under the Background of Serious Epidemic Based on the Qualitative Study of 255 Psychological Help Cases by Nvivo. Journal of Guangzhou Radio and Television University, 20(4), 4048+108-109.

Liu Zhenlu. (2020). A study on the physical and health status of college students and its influencing factors (Master's thesis, Dalian University of Technology)

Liu Zuhong. (2020). Physical exercise influences college students' sleep quality, anxiety and depression research (a master's degree thesis, the Shanghai sports institute).

Liu, S., Li, L., Liu, Z., & Guo, X. (2019). Long-term Tai Chi experience promotes emotional stability and slows gray matter atrophy for elders. Frontiers in psychology, 10, 91.

Liu, X., L. Vitetta, K. Kostner, D. Crompton, G. Williams, W. J. Brown, A. Lopez, C. C. Xue, T. P. Oei, G. Byrne, J. H. Martin and H. Whiteford. (2015). "The

effects of tai chi in centrally obese adults with depression symptoms." Evid Based Complement Alternat Med 2015: 879712.

Liu, Y., Mimura, K., Wang, L., et al. (2003). Physiological benefits of 24-style Taijiquan exercise in middle-aged women. Journal of physiological anthropology and applied human science, 22(5), 219-225.

Liu, Z., Li, L., Liu, S., et al. (2020). Reduced feelings of regret and enhanced fronto-striatal connectivity in elders with long-term Tai Chi experience. Social cognitive and affective neuroscience, 15(8), 861-873.

Loades, M. E., Chatburn, E., Higson-Sweeney, N., Reynolds, S., Shafran, R., Brigden, A.,... & Crawley, E. (2020). Rapid systematic review: the impact of social isolation and loneliness on the mental health of children and adolescents in the context of COVID-19. Journal of the American Academy of Child & Adolescent Psychiatry, 59(11), 1218-1239.

Lokken, K. L., Boeka, A. G., Austin, H. M., Gunstad, J., & Harmon, C. M. (2009). Evidence of executive dysfunction in extremely obese adolescents: a pilot study. Surgery for Obesity and Related Diseases, 5(5), 547-552.

Lokken, K. L., Boeka, A. G., Yellumahanthi, K., Wesley, M., & Clements, R. H. (2010). Cognitive performance of morbidly obese patients seeking bariatric surgery. The American Surgeon, 76(1), 55-59.

Lomas-Vega, R., Obrero-Gaitan, E., Molina-Ortega, F. J., & Del-Pino-Casado, R. (2017). Tai Chi for risk of falls. A meta-analysis. Journal of the American Geriatrics Society, 65(9), 2037-2043.

Lowry, R., Galuska, D. A., Fulton, J. E., Wechsler, H., Kann, L., & Collins, J. L. (2000). Physical activity, food choice, and weight management goals and practices among US college students. American journal of preventive medicine, 18(1), 18-27.

Lu, X., Siu, K. C., Fu, S. N., Hui-Chan, C. W., & Tsang, W. W. (2016). Effects of Tai Chi training on postural control and cognitive performance while dual tasking–a randomized clinical trial. Journal of complementary and integrative medicine, 13(2), 181-187.

Lu, Y.-Z. The Current Physical and Mental Health of High School Students is

Facing Problems and Countermeasures. First Middle Sch. Mudanjiang City Heilongjiang Prov. 2017, 13, 2.

M. Adcock, M.(2019). Fankhauser, J. Post et al., “Effects of a in home multicomponent exercise training on physical functions, cognition, and brain volume of older adults: a randomized controlled trial,” Frontiers in Medicine, vol., 6, p. 321.

MacLeod, C. M. (1991). Half a century of research on the Stroop effect: An integrated review. Psychological Bulletin, 109(2), 163–203.

MacLeod, C. M. (2015). The stroop effect. In R. Luo (Ed.), Encyclopedia of Color Science and Technology (pp. 1–6). Springer Science and Business Media.

Magallares, A. and J. L. Pais-Ribeiro. (2013). "Mental Health and Obesity: A Meta-Analysis." Applied Research in Quality of Life 9(2): 295-308.

Malina R (2010). Physical activity and health of youth. Constanta: Ovidius University Annals, Series Physical Education and Sport/Science, Movement and Health.

Mao, J. J., Hung, K. W., et al.. (2021). Implementing virtual mind-body programming to support cancer patients during COVID-19. Journal of Clinical Oncology, 15(39): 1585-1585.

Mazurek Melnyk, B., Slevin, C., Militello, L., et al. (2016). Physical health, lifestyle beliefs and behaviors, and mental health of entering graduate health professional students: Evidence to support screening and early intervention. Journal of the American Association of Nurse Practitioners, 28(4), 204-211.

Mclaughlin, M., Atkin, A. J., Starr, L., Hall, A., Wolfenden, L., Sutherland, R., Wiggers, J., Ramirez, A., Hallal, P., Pratt, M., Lynch, B. M., Wijndaele, K., & Sedentary Behaviour Council Global Monitoring Initiative Working Group (2020). Worldwide surveillance of self-reported sitting time: a scoping review. The international journal of behavioral nutrition and physical activity, 17(1), 111.

methamphetamine/what-are-long-term-effects-methamphetamine-misuse on 2021, July 30

Miller BL (2007) The human frontal lobes: An introduction. In: The Human Frontal Lobes: Functions and Disorders, MillerBL, CummingsJL, Eds. Guilford Press,

New York, NY, pp 3-12.

Miller J, Kransler J, Liu Y, Schmalfuss I, et al. (2006). Neurocognitive findings in Prader-Willi syndrome and early-onset morbid obesity. J Pediatr, 149: 192–198.

Miller JL, Couch J, Schwenk K, et al. (2009). Early childhood obesity is associated with compromised cerebellar development. Dev Neuropsychol, 34: 272–283.

Miller SM, Taylor-Piliae RE.(2014).Effects of tai chi on cognitive function in community-dwelling older adults: a review. Geriatr Nurs, 35(1): 9-19.

Mond JM, Stich H, Hay PJ, Kraemer A, Baune BT.(2007).Associations between obesity and developmental functioning in pre-school children: a population-based study. Int J Obes, 31: 1068–1073.

Montacute, R., & Holt-White, E. (2020). Research Brief: May 2020: COVID-19 and Social Mobility Impact Brief# 2: University Access & Student Finance.

Morrow, K., Bowman, N., Cramer, M.,et al. (2021). Structured Resistance Training Improves Self-Esteem and Strength in Previously Untrained College Females. In International Journal of Exercise Science: Conference Proceedings ,9(9) : 27.

Nakatani, Y., Fumoto, M., Yu, X., Kikuchi, H., Nakasato, A., Seki, Y., ... & Arita, H. (2006). Effects of Tai Chi exercise on human EEG and regional cerebral blood flow (CBF): Contribution of brain serotonergic system. In Proceedings of Annual Meeting of the Physiological Society of Japan Proceedings of Annual Meeting of the Physiological Society of Japan (pp. 233-233). PHYSIOLOGICAL SOCIETY OF JAPAN.

Nelson, T. F., Gortmaker, S. L., Subramanian, S. V., & Wechsler, H. (2007). Vigorous physical activity among college students in the United States. Journal of physical activity & health, 4(4), 495-508.

Newman, M. G., Jacobson, N. C., Rackoff, G. N., Bell, M. J., & Taylor, C. B. (2020). A randomized controlled trial of a smartphone-based application for the treatment of anxiety. Psychotherapy Research, 1-12.

Nguyen, M.H.; Kruse.(2012), A. The effects of Tai Chi training on physical

fitness, perceived health, and blood pressure in elderly Vietnamese. Open Access J. Sports Med. 3, 7–16.

NIDA. 2021, April 13. What are the long-term effects of methamphetamine misuse?. Retrieved from https://www.drugabuse.gov/publications/research-reports/

Obesity and Overweight for Professionals: Causes. Centers for Disease Control and Prevention. Archived from the original on February 24, 2016. Retrieved January 19, 2010.

Okorodudu; et al. (2010). "Diagnostic performance of body mass index to identify obesity as defined by body adiposity: a systematic review and meta-analysis". International Journal of Obesity. 34 (5): 791–799.

Palermi, S.; Sacco, A.M.; Belviso, I.; et al. (2020).Guidelines for Physical Activity—A Cross-Sectional Study to Assess Their Application in the General Population. Have We Achieved Our Goal? Int. J. Environ. Res. Public Health, 17, 3980.

Pan, Y.-J.; Bi, J.-P. (2009). Research on the Application of Exercise Prescriptions for Civilization Diseases in my Country in Recent Years. Hubei Sports Sci. Technol, 28, 540.

Pan, Z.; X. Su; Q. Fang; et al.(2018). "The Effects of Tai Chi Intervention on Healthy Elderly by Means of Neuroimaging and EEG: A Systematic Review." Front Aging Neurosci 10: 110.

Patterson R, McNamara E, Tainio M, et al. (2018). "Sedentary behaviour and risk of all-cause, cardiovascular and cancer mortality, and incident type 2 diabetes: a systematic review and dose response meta-analysis". European Journal of Epidemiology. 33 (9): 811-829.

Pengpid S, Peltzer K, Kassean HK, et al.(2015).Physical inactivity and associated factors among university students in 23 low-, middle- and high-income countries. Int J Public Health, 60:539–549.

Physical inactivity a leading cause of disease and disability, warns WHO. (2010).World Health Organization. Retrieved January 23, 2010.

Powell, K. E. (1988). Habitual exercise and public health: An epidemiological

view. Exercise adherence: Its impact on public health, 15-39.

Pribis, P., Burtnack, C. A., McKenzie, S. O., & Thayer, J. (2010). Trends in body fat, body mass index and physical fitness among male and female college students.Nutrients, 2(10), 1075-1085.

Prickett, C., Brennan, L., & Stolwyk, R. (2015). Examining the relationship between obesity and cognitive function: a systematic literature review. Obesity research & clinical practice, 9(2), 93-113.

Q. Zhu, W. Cai, J. Zheng et al., (2016).Distinct resting-state brain activity in patients with functional constipation, Neuroscience Letters, vol., 632, pp. 141 –146.

R. Jalilianhasanpour, E. Beheshtian, G. Sherbaf, S. Sahraian, and H.I. Sair, (2019). Functional connectivity in neurodegenerative disorders: Alzheimer’s disease and frontotemporal dementia, Topics in Magnetic Resonance Imaging, 28(6): 317–324.

R. Silveira, R.(2019). C. R. Prado, C. Brietzke et al., “Prefrontal cortex asymmetry and psychological responses to exercise: a systematic review,” Physiology & Behavior, vol., 208, p. 112580.

Raichle, M. E. (2015). The brain's default mode network. Annual review of neuroscience, 38, 433-447.

Rezaei, M. (2019). Neuropsychological decomposing Stroop interference into different cognitive monitoring: An exploratory factor analysis. Basic and clinical neuroscience, 10(5), 475.

Riebe, D., Ehrman, J. K., Liguori, G., Magal, M., & American College of Sports Medicine (Eds.). (2018). ACSM's guidelines for exercise testing and prescription. Wolters Kluwer.

Roberts, R., Strawbridge, W., Deleger, S., & Kaplan, G. (2002). Are the fat more jolly? Annals Of Behavioral Medicine, 24, 169–80.

Russell-Mayhew, S., G. McVey, A. Bardick and A. Ireland (2012). "Mental health, wellness, and childhood overweight/obesity." J Obes 2012: 281801.

S. Deepeshwar, S. A. Vinchurkar, N. K.(2014). Visweswaraiah, and H. R. Nagendra, “Hemodynamic responses on prefrontal cortex related to meditation and

attentional task," Frontiers in Systems Neuroscience, vol., 8, p. 252.

Sacheck, J.M.; Kuder, J.F.; Economos, C.D. (2020). Physical fitness, adiposity, and metabolic risk factors in young college students. Med. Sci. Sports Exerc, 42, 1039-1044.

Sahinoglu, B., & Dogan, G. (2016). Event-related potentials and the Stroop effect. The Eurasian Journal of Medicine, 48(1), 53–57.

Salthouse T A. (2010).Selective review of cognitive aging. J Int Neuropsychol Soc. 16(5):754–760.

Saphira, C. D. R., Widodo, S., Wati, A. P., & Sumekar, T. A. (2021). The Effect of High Intensity Interval Training (HIIT) on Reaction Time Study Among Medical Student in Semarang. DIPONEGORO MEDICAL JOURNAL (JURNAL CATEGORY DIPONEGORO), 10(3).

Sawka, M.N.; Leon, L.R.; Montain, S.J.; Sonna, L.A. Integrated Physiological Mechanisms of Exercise Performance, Adaptation, and Maladaptation to Heat Stress. Compr. Physiol. 2011, 1, 1883–1928.

Seligman ME, Walker EF, Rosenhan DL. Abnormal psychology(4th ed.). New York: W.W. Norton & Company.

Shaw, K.; Gennat, H.; O'Rourke, P.; Del Mar, C. (2006). Exercise for overweight or obesity. Cochrane Database Syst. Rev., 4, CD003817.

Shen, C. C., Tseng, Y. H., Shen, M. C. S., & Lin, H. H. (2021). Effects of Sports Massage on the Physiological and Mental Health of College Students Participating in a 7-Week Intermittent Exercises Program. International Journal of Environmental Research and Public Health, 18(9), 5013.

Shen, C. C., Tseng, Y. H., Shen, M. C. S., & Lin, H. H. (2021). Effects of Sports Massage on the Physiological and Mental Health of College Students Participating in a 7-Week Intermittent Exercises Program. International Journal of Environmental Research and Public Health, 18(9), 5013.

Sibley, B. A., & Etnier, J. L. (2003). The relationship between physical activity and cognition in children: A meta-analysis. Pediatric Exercise Science, 15, 243-256.

Solianik, R., Mickevičienė, D., Žlibinaitė, L., & Čekanauskaitė, A. (2021). Tai

chi improves psychoemotional state, cognition, and motor learning in older adults during the COVID-19 pandemic. Experimental gerontology, 150, 111363.

Song, Q. H., Shen, G. Q., Xu, R. M., et al. (2014). Effect of Tai Chi exercise on the physical and mental health of the elder patients suffered from anxiety disorder. International journal of physiology, pathophysiology and pharmacology, 6(1), 55.

Sontáková, L., Bártová, A., Daďová, K., Holmerová, I., & Šteffl, M. (2021). Effects of physical exercise on cognitively impaired older adults: a systematic review. AUC KINANTHROPOLOGICA, 57(1), 51-78.

Spielberger, C. D. (1970). Manual for the State-trait Anxietry, Inventory. Consulting Psychologist.

Spielberger, C. D., Gorsuch, R. L., Lushene, R., Vagg, P. R., & Jacobs, G. A. (1983). Manual for the State-Trait Anxiety Inventory. Palo Alto, CA: Consulting Psychologists Press.

Statistics, N.C.f.E., Nontraditional Undergraduates, U.S.D.o.E. Institute of Education Sciences, Editor 2013.

Stockwell, S., Trott, M., Tully, M., et al. (2021). Changes in physical activity and sedentary behaviours from before to during the COVID-19 pandemic lockdown: a systematic review. BMJ Open Sport & Exercise Medicine, 7(1), e000960.

Stroop, J. R. (1935). Studies of interference in serial verbal reactions. Journal of Experimental Psychology, 18(6), 643–662.

Sultan, M. S., Khan, A. H., Hossain, S., et al. (2021). Mental health difficulties in students with suspected COVID-19 symptoms and students without suspected COVID-19 symptoms: A cross-sectional comparative study during the COVID-19 Pandemic. Children and Youth Services Review, 106137.

Sun, Y., Zhou, N., Wang, X., Yu, X., Yang, J., & Bai, X. (1988). Rehabilitation protocol on cardiovascular diseases and effectiveness evaluation in 20 cases. Chinese Journal of Rehabilitation Medicine, 8, 2-5.

Sunderland, A., Watts, K., Baddeley, A. D., & Harris, J. E. (1986). Subjective memory assessment and test performance in elderly adults. Journal of Gerontology, 41(3), 376-384.

Sungkarat S, Boripuntakul S, Chattipakorn N, et al. (2017). Effects of tai chi on cognition and fall risk in older adults with mild cognitive impairment: A randomized controlled trial. J Am Geriatr Soc 65: 721-727.

Swanson, J. (2005). The Delis-Kaplan executive function system: A review. Canadian Journal of School Psychology, 20(1-2), 117–128.

T. Moriarty, K. Bourbeau, B. Bellovary, et al.(2019). Zuhl,"Exercise intensity influences prefrontal cortex oxygenation during cognitive testing," Behavioral Sciences, vol., 9, no. 8,p. 83.

Tang, H., Gu, L. (2012). The history and development of tai chi. In Study on Tai Chi. Beijing: People's Sport Publishing House of China.

Teychenne M, Costigan SA, Parker K (2015). The association between sedentary behaviour and risk of anxiety: a systematic review. BMC Public Health. 15: 513.

Tian Y, Jiang C, Wang M, et al.BMI, leisure-time physical activity, and physical fitness in adults in China: results from a series of national surveys, 2000-14[J]. Lancet Diabetes Endocrinol, 2016,4(6): 487-497.

Timeline: WHO COVID-19 response. Retrieved from https://www.who.int/emergencies/diseases/novel-coronavirus-2019/interactive-timeline on 2021, July 30

Tremblay MS, Aubert S, Barnes JD, et al. (2017).Sedentary behavior research network (SBRN) – terminology consensus project process and outcome. Int J Behav Nutr Phys Act, 14(1): 75.

Tremblay MS, Colley RC, Saunders TJ, et al. (2010). Physiological and health implications of a sedentary lifestyle. Applied Physiology, Nutrition, and Metabolism. 35 (6): 725–40.

Ugalde, A., Krishnasamy, M., & Schofield, P. (2014). The relationship between self-efficacy and anxiety and general distress in caregivers of people with advanced cancer. Journal of Palliative Medicine, 17(8), 939-41.

US Department of Health and Human Services. (2018). Physical activity guidelines advisory committee scientific report. 2018. C9-C10.

Vaingankar, J. A., Rekhi, G., Subramaniam, M., et al. (2013). Age of onset of

life-time mental disorders and treatment contact. Social psychiatry and psychiatric epidemiology, 48(5), 835-843.

Verdejo-Garcia A, Perez-Exposito M, Schmidt-Rio-Valle J, et al. (2010). Selective alterations within executive functions in adolescents. Obesity (Silver Spring) , 18: 1572–1578.

Vitasari, P., Wahab, M. N. A., Herawan, T., Othman, A., & Sinnadurai, S. K. (2011). Re-test of State Trait Anxiety Inventory (STAI) among engineering students in Malaysia: reliability and validity tests. Procedia-Social and Behavioral Sciences, 15, 3843-3848.

Voss, M.W., Nagamatsu, L.S., Liu-Ambrose, T., & Kramer, A.F. (2011). Exercise, brain, and cognition across the life span. Journal of Applied Physiology, 111, 1505–1513.

Wall, R. B. (2005). Tai Chi and mindfulness-based stress reduction in a Boston Public Middle School. Journal of Pediatric Health Care, 19(4), 230–237.

Wang C, Bannuru R, Ramel J, Kupelnick B, Scott T, Schmid CH.(2010). Tai Chi on psychological well-being: systematic review and meta-analysis. BMC Complement Altern Med. 10:23.

Wang, C., Collet, J. P., & Lau, J. (2004). The effect of Tai Chi on health outcomes in patients with chronic conditions: a systematic review. Archives of internal medicine, 164(5), 493-501.

Wang, D. (1958). The medical application of tai chi in tuberculosis (TB). Chinese Journal of Antituberculosis, 5, 33-36.

Wang, J. (2019). The association between physical fitness and physical activity among Chinese college students. Journal of American College Health, 67(6), 602-609.

Wang, X., & Ji, X. (2020). Sample size estimation in clinical research: from randomized controlled trials to observational studies.Chest, 158(1), S12-S20.

Wang, Z. H., Dong, Y. H., Song, Y., Yang, Z. P., & Ma, J. (2017). Analysis on prevalence of physical activity time< 1 hour and related factors in students aged 9-22 years in China, 2014. Zhonghua liu bing bing xue za zhi= Zhonghua liuxingbingxue zazhi, 38(3), 341-345.

Wayne PM, Kaptchuk TJ.(2008). Challenges inherent to tai chi research: part II defining the intervention and optimal study design[J] J Altern Complement Med., 14: 191–197.

Wayne PM, Walsh JN, Taylor-Piliae RE, et al.(2014). Effect of tai chi on cognitive performance in older adults: systematic review and meta-analysis. J Am Geriatr Soc. 62(1): 25-39.

Wayne, P. M., Gow, B. J., Costa, M. D., Peng, C. K., Hausdorff, J. M., Lipsitz, L. A., ... & Manor, B. (2014). Do Complexity-based measures of sway inform long-and short-term effects of Tai Chi training on balance in healthy adults?. The Journal of Alternative and Complementary Medicine, 20(5), A25-A25.

Wayne, P. M., Manor, B., Novak, V., Costa, M. D., et al., (2013). A systems biology approach to studying tai chi, physiological complexity and healthy aging: Design and rationale of a pragmatic randomized controlled trial. Contemporary Clinical Trials, 34(1), 21-34.

Wei, L., Chai, Q., Chen, J., et al. (2020). The impact of Tai Chi on cognitive rehabilitation of elder adults with mild cognitive impairment: a systematic review and meta-analysis. Disability and Rehabilitation, 1-10.

Weller RE, Cook Iii EW, Avsar KB, Cox JE.(2008). Obese women show greater delay discounting than healthy-weight women. Appetite,51(3): 563-9.

Xiang, M. Q., Tan, X. M., Sun, J., et al.(2020). Relationship of physical activity with anxiety and depression symptoms in Chinese college students during the COVID-19 outbreak. Frontiers in psychology, 11, 2860.

Xiang, M.; Zhang, Z.; Kuwahara, K. Impact of COVID-19 Pandemic on Children and Adolescents' Lifestyle Behavior Larger than Expected. Public Health Emergency. Collect. 2020, 63, 531–532.

Xie, H., Zhang, M., Huo, C., Xu, G., Li, Z., & Fan, Y. (2019). Tai Chi Chuan exercise related change in brain function as assessed by functional near–infrared spectroscopy. Scientific reports, 9(1), 1-14.

Xu, P., Wu, D., Zhou, Y., Wu, J., & Xiao, W. (2021). An event-related potential (ERP) study of the transfer of response inhibition training to interference control.

Experimental Brain Research, 239(4), 1327-1335.

Xu, Z., Zhang, D., Lee, A. T., et al. (2020). A pilot feasibility randomly controlled trial on combining mind-body physical exercise, cognitive training, and nurse-led risk factor modification to reduce cognitive decline among older adults with mild cognitive impairment in primary care.PeerJ, 8, e9845.

Yang, G. Y., Wang, L. Q., Ren, J., et al. (2015). Evidence base of clinical studies on Tai Chi: a bibliometric analysis. Plos one, 10(3), e0120655.

Yang, G., Wang, L., Ren, J., et al., (2015). Evidence base of clinical studies on tai chi: A bibliometric analysis. PLoS One, 10(3), e0120655.

Yeung A, Lepoutre V, Wayne P, et al.(2012). Tai Chi treatment for depression in Chinese Americans: a pilot study. Am J Phys Med Rehabil 91(10): 863-70.

Yeung AS, Feng R, Kim DJH, et al.(2017). A pilot, randomized controlled study of Tai Chi with passive and active controls in the treatment of depressed Chinese Americans. J Clin Psychiatry 78(5): e522-e528.

Yu AP, Tam BT, Lai CW, et al. (2018). Revealing the neural mechanisms underlying the beneficial effects of tai chi: A neuroimaging perspective. Am J Chin Med 46: 231-259.

Yu, N., Chen, C., Liu, C., et al.(2011). Association of body mass index and depressive symptoms in a Chinese community population: results from the Health Promotion Knowledge, Attitudes, and Performance Survey in Taiwan. Medical Journal, 34, 620–627.

Yuesti, A., Ni, W. R., & Suryandari, N. N. A. (2020). Financial literacy in the COVID-19 pandemic: pressure conditions in Indonesia. Entrepreneurship and Sustainability Issues, 8(1), 884.

Z. Zheng, X. Zhu, S. et al., (2015).Combined cognitive-psychological-physical intervention induces reorganization of intrinsic functional brain architecture in older adults," Neural Plasticity, vol. 2015, Article ID, 713104, 11 pages.

ZHANG Hai-jun, WANG Hao-chuan. (2008).Study on Multi-lead EEG Signal Classification and Recognition [J]. Computer Engineering and Applications, (24): 228-230.

Zhang, D., Hu, M., & Ji, Q. (2020). Financial markets under the global pandemic of COVID-19. Finance Research Letters, 36, 101528.

Zhang, Y., Zhang, H., Ma, X., & Di, Q. (2020). Mental health problems during the COVID-19 pandemics and the mitigation effects of exercise: a longitudinal study of college students in China. International journal of environmental research and public health, 17(10), 3722.

Zheng Fuzhen, Yang Caihong. The birthplace of Chinese Taijiquan is Wenxian County, Henan Province. Henan: Elephant Press, 2009

Zheng G, Liu F, Li S, Huang M, Tao J, Chen L. Tai chi and the protection of cognitive ability: a systematic review of prospective studies in healthy adults. Am J Prev Med 2015;49(1): 89-97.

Zheng, G., Lan, X., Li, M., et al., (2014). The effectiveness of Tai Chi on the physical and psychological well-being of college students: a study protocol for a randomized controlled trial. Trials, 15(1), 1-9.

Zhu, M. (2020, November). The Effect of Language Experience on Stroop Effect by Chinese English Learners. In 2020 Conference on Education, Language and Inter-cultural Communication (ELIC 2020) (pp. 452-456). Atlantis Press.

Zisook S et al. Effect of age at onset on the course of major depressive disorder. Am J Psychiatry. 2007;164(10): 1539-46

Zou L, Yeung A, Li C, Wei GX, Chen KW, Kinser PA, et al.(2018). Effects of meditative movements on major depressive disorder: a systematic review and meta-analysis of randomized controlled trials. J Clin Med. 7(8): 195.

Zou, L., Loprinzi, P. D., Yu, J. J., Yang, L., Li, C., Yeung, A. S., ... & Xiao, T. (2019). Superior effects of modified chen-style tai chi versus 24-style tai chi on cognitive function, fitness, and balance performance in adults over 55. Brain sciences, 9(5), 102.

Zou, L., Sasaki, J. E., Wei, G. X., et al. (2018). Effects of mind–body exercises (Tai Chi/Yoga) on heart rate variability parameters and perceived stress: A systematic review with meta-analysis of randomized controlled trials. Journal of Clinical Medicine, 7(11), 404.

Zou, L.; Wang, H.; Yu, D.(2017). Effect of a long-term modified Tai Chi-based intervention in attenuating bone mineral density in postmenopausal women in southeast China: Study protocol for a randomized controlled trial. Clin. T rials Degener. Dis. 2, 46–52.

Zulkifli, N. A., Mohammad, S. N. S., Rashid, M. R. A., & Mohideen, F. B. S. (2021). Tai Chi to Preserve General Health of Elderly during the COVID-19 Pandemic. Ulum Islamiyyah, 57-76.

Zung, W. W. (1965). A self-rating depression scale. Archives of general psychiatry, 12(1), 63-70.

Zwart PL, Jeronimus BF, de Jonge P, et al. (2019). "Empirical evidence for definitions of episode, remission, recovery, relapse and recurrence in depression: a systematic review". Epidemiology and Psychiatric Sciences. 28 (5): 544–562.

Appendices

Appendix A

International Physical Activity Questionnaire (IPA-Q)

Participant ID: __________________ Gender: ☐ Male ☐ Female
Age: : __________________

We are interested in finding out about the kinds of physical activities that people do as part of their everyday lives. The questions will ask you about the time you spent being physically active in the last 7 days. Please answer each question even if you do not consider yourself to be an active person. Please think about the activities you do at work, as part of your house and yard work, to get from place to place, and in your spare time for recreation, exercise or sport.

Think about all the vigorous activities that you did in the last 7 days. Vigorous physical activities refer to activities that take hard physical effort and make you breathe much harder than normal. Think only about those physical activities that you did for at least 10 minutes at a time.

1. During the last 7 days, on how many days did you do vigorous physical activities like heavy lifting, digging, aerobics, or fast bicycling?

A. Yes,_____ days per week

B. No vigorous physical activities Skip to question 3

2. How much time did you usually spend doing vigorous physical activities on one of those days? _____ hours per day

Think about all the moderate activities that you did in the last 7 days. Moderate activities refer to activities that take moderate physical effort and make you breathe somewhat harder than normal. Think only about those physical activities that you did

for at least 10 minutes at a time.

3. During the last 7 days, on how many days did you do moderate physical activities like carrying light loads, bicycling at a regular pace, or doubles tennis? Do not include walking.

A. Yes,_____ days per week

B. No moderate physical activities Skip to question 5

4. How much time did you usually spend doing moderate physical activities on one of those days? _____ hours per day

Think about the time you spent walking in the last 7 days. This includes at work and at home, walking to travel from place to place, and any other walking that you might do solely for recreation, sport, exercise, or leisure.

5. During the last 7 days, on how many days did you walk for at least 10 minutes at a time?

A. Yes,_____ days per week

B. No walking Skip to question 7

6. How much time did you usually spend walking on one of those days?

_____ hours per day

The last question is about the time you spent sitting on weekdays during the last 7 days. Include time spent at work, at home, while doing course work and during leisure time. This may include time spent sitting at a desk, visiting friends, reading, or sitting or lying down to watch television.

7. During the last 7 days, how much time did you spend sitting on a week day? Yes,_______ minutes per day

This is the end of the questionnaire, thank you for participating.

Appendix A (Chinese Version)

国际体力活动问卷 (IPAQ)

编号：________________________　　　　性别：☐ 男　☐ 女

年龄：_________________岁

目的:了解人们在日常生活中所做的各种体育活动。这些问题会问你在过去 7 天里做了多少运动。请回答每一个问题，即使你不认为自己是一个积极的人。请想想你在工作中做的活动，作为你的房子和院子工作的一部分，从一个地方到另一个地方，以及在你的空闲时间娱乐，锻炼或运动。

1. 过去 7 天中你是否进行过剧烈的体力活动，如搬举重物、上楼梯、爬山、用锄头锄土、搬堆家具、快速骑自行车、剧烈体育锻炼（跑步、跳绳、健身房内健身操等）且持续时间超过 10 分钟？

是，天数为________　　　　　　否，跳到问题 3

2. 在这几天中，你每天进行这些剧烈体力活动的时间是多少？ ___分钟/天

3. 在过去 7 天中，你是否进行过中等强度体力活动，如搬举轻物、整理庭院/阳台、用手擦地、用手洗衣服、清洗门窗用大剪刀修剪花草、铺床、下楼梯、打羽毛球、乒乓球、一般速度骑自行车（当作交通工具）、做瑜伽、练太极拳等且每次活动持续时间超过 10 分钟？

是，天数为________　　　　　　否，跳到问题 5

4. 在这几天中，你每天进行这些中等强度体力活动的时间是多少？____分钟/天

5. 在过去的 7 天中你是否有每次至少 10 分钟的步行？包括您工作时、在家中的步行、交通行程的步行以及为了锻炼身体进行的步行。

是，天数为________　　　　　　否，跳到问题 7

6. 在这几天中，你每天步行的时间是多少？　________分钟/天

7. 在过去的 7 天中，你通常每天处于久坐的身体状态有多长时间？包括在家、工作场所及闲暇时间里，可能出现在拜访亲友、看书读报、坐着或躺卧看电视以及坐在电脑前（不包括睡眠时间）。　________分钟/天

Appendix B

Physical Activity Readiness Questionnaire（PAR-Q）

Participant ID: ____________________ Gender: ☐ Male ☐ Female
Age: : __________________

Purpose: This stage is mainly used for self-assessment.Identify the subjects' known ailments or symptoms of diseases that may be at high risk during tai chi exercise.

Topic	Please tick(√)	
1.Has your doctor ever told you that you have a heart attack or that you've had a stroke?	Yes	No
2. Have you ever experienced excruciating pain in your chest while resting or while exercising?	Yes	No
3. Have you ever lost your balance due to dizziness or fainting while exercising?	Yes	No
4. Have you had an asthma attack in the past 12 months that required immediate medical attention?	Yes	No
5. Have you ever had a diagnosed muscle, bone, joint or neurological problem that you were told might get worse as a result of participating in physical exercise?	Yes	No
6. Do you know that you have other problems that make you unfit for sports?	Yes	No
7. Have you practiced Tai Chi or participated in any meditation exercise in the past 3 months?	Yes	No

If you answered "yes" to any of the 7 questions, please seek guidance from your family doctor or appropriate allied health professional before engaging in physical activity. If you answered "no" to all of the seven questions, and you have no other health concerns, then you can do light moderate physical activity exercise.

I believe, to the best of my knowledge, that all the information I have provided in this tool is correct.

Appendix B (Chinese Version)

身体状况问卷(PARQ)

编号：________________________　　　　性别：☐ 男　☐ 女

年龄：__________________岁

目的:这个阶段主要用于自我评估。确认受试者的已知疾病或者疾病的症状,在太极运动的过程中,这些疾病可能具有很高的风险性。我相信,据我所知,我在这个工具中提供的所有信息都是正确的。

题目	打勾(√)	
1. 你的医生是否曾经告诉过你有心脏病或者你曾经是否遗受过中风?	是	否
2. 当你在休息的时候或者运动的过程中,你的胸部是否经历过难以忍受的疼痛?	是	否
3. 当你运动的过程中,你是否有过头晕或者昏厥导致你失去平衡?	是	否
4. 在过去的 12 个月中,你是否有哮喘发作需要立即的医疗治疗?	是	否
5. 你有没有被诊断过的肌肉、骨骼关节或神经类问题,你被告知可能是否会因为参加体育锻炼而变得更糟?	是	否
6. 你是否知道自己还有其他的问题，导致不适合参加运动?	是	否
7. 你有没有习练过太极，在过去 3 个月内是否参加过冥想类运动?	是	否

如果您对 7 个问题中的任何一个都回答“是”,请在进行体力活动之前向您的家庭医生或适当的专职保健专业人员寻求指导。

如果你对 7 个问题中的所有问题都回答“不”,并且你对健康没有其他的担心,那么你可以进行轻度中等强度的体力活动锻炼。

Appendix C

Depression Status Inventory

Participant ID: ____________________ Gender: ☐ Male ☐ Female
Age: : __________________

For each statement below, please circle the number in the column that best represents how you have been feeling or behaving in the last several days.

Topic	A little of the time	Some of the time	A Good part of the time	Most of the time
	Please tick(√)			
1. I feel down-hearted and blue.	1	2	3	4
2. Morning is when I feel the best.	4	3	2	1
3. I have crying spells or feel like it.	1	2	3	4
4. I have trouble sleeping at night	1	2	3	4
5. I eat as much as I used to.	4	3	2	1
6. I still enjoy sex.	4	3	2	1
7. I notice that I am losing weight.	1	2	3	4
8. I have trouble with constipation.	1	2	3	4
9. My heart beats faster than usual.	1	2	3	4
10. I get tired for no reason.	1	2	3	4
11. My mind is as clear as it used to be.	4	3	2	1
12. I find it easy to do the things I used to.	4	3	2	1
13. I am restless and can't keep still.	1	2	3	4

14. I feel hopeful about the future.	4	3	2	1
15. I am more irritable than usual.	1	2	3	4
16. I find it easy to make decisions.	4	3	2	1
17. I feel that I am useful and needed.	4	3	2	1
18. My life is pretty full.	4	3	2	1
19. I feel that others would be better off if I were dead.	1	2	3	4
20. I still enjoy the things I used to do.	4	3	2	1

Add up all of the numbers that were circled and consult the scale provided below:

The scores range from 25-100. ™

25-49 Normal Range ™

50-59 Mildly Depressed ™

60-69 Moderately Depressed ™

70 and above Severely Depressed

Appendix C (Chinese Version)

抑郁状态问卷(DSI)

编号：________________________　　性别：☐ 男　☐ 女

年龄：__________________岁

指导语：DSI 由 20 个陈述句和相应问题条目的组成，请被测试者根据最近一周的情况，对每一个条目均按 1、2、3、4 四级给予评分，请如实回答以下 20 项症状。

题目	偶尔或无	有时	经常	持续
	打勾(√)			
1. 你感到情绪沮丧，郁闷吗?	1	2	3	4
2. 你要哭或想哭吗?	4	3	2	1
3. 你感到早晨心情最好吗?	1	2	3	4
4. 你夜间睡眠不好吗?经常早醒吗？	1	2	3	4
5. 你吃饭象平时一样多吗?食欲如何?	4	3	2	1
6. 你感到体重减轻了吗?	4	3	2	1
7.你的性功能正常吗?乐意注意具有吸引力的异性，并好和他/她在一起、说话吗？	1	2	3	4
8. 你为便秘烦恼吗?	1	2	3	4
9. 你的心跳比平时快吗？	1	2	3	4
10. 你无故感到疲劳吗?	1	2	3	4
11. 你坐卧不安，难以保持平静吗?	4	3	2	1
12. 你做事情比平时慢吗？	4	3	2	1
13. 你的头脑象往常一样清楚吗?	1	2	3	4

14. 你感到生活很空虚吗?	4	3	2	1
15. 你对未来感到有希望吗?	1	2	3	4
16. 你觉得决定什么事很容易吗?	4	3	2	1
17. 你比平时更容易激怒吗?	4	3	2	1
18. 你仍旧喜爱自己平时喜爱的事情吗?	4	3	2	1
19. 你感到自己是有用的和不可缺少的人吗?	1	2	3	4
20. 你曾经想过自杀吗?	4	3	2	1

注:1972年Zung氏增编了与自评抑郁量表(Self-Rating Depression Scale，SDS)相应的检查者用本,改自评为他评,称为抑郁状态问卷(Depression Status Inventory，DSI)。评定时间跨度为最近一周。DSI由20个陈述句和相应问题条目的组成。每一条目相当于一个有关症状，按1-4级评分。

评分方法：每一个条目均按1、2、3、4四级评分。20个条目中有10项(第2、5、6、11、12、14、16、17、18和20)是用正性词陈述的，为反序计分，其余10项是用负性词陈述的，按上述1-4顺序评分。

条目反映抑郁状态四组特异性症状：

1. 精神性——情感症状，包含抑郁心境和哭泣两个条目；

2. 躯体性障碍：包含情绪的日间差异、睡眠障碍、食欲减退、性欲减退、体重减轻、便秘、心动过速、易疲劳共八个条目；

3. 精神运动性障碍，包含精神运动性迟滞和激越两个条目；

4. 抑郁的心理障碍，包含思维混乱、无望感、易激惹、犹豫不决、自我贬值、空虚感、反复思考自杀和不满足，共八个条目。

Appendix D

State-Trait Anxiety Inventory for Adults

STAI Form Y-1 and Form Y-2

Participant ID: ____________________ Gender: ☐ Male ☐ Female
Age: : __________________

STAI Form Y-1

DIRECTIONS: A number of statements which people have used to describe themselves are given below. Read each statement and then circle the appropriate number to the right of the statement to indicate how you feel right now, that is, at this moment. There are no right or wrong answers. Do not spend too much time on any one statement but give the answer which seems to describe your present feelings best.

Topic	Not at all	Somewhat	Moderately so	Very much so
	Please tick(√)			
1. I feel calm.	1	2	3	4
2. I feel secure.	4	3	2	1
3. I am tense.	1	2	3	4
4. I feel strained.	1	2	3	4
5. I feel at ease.	4	3	2	1
6. I feel upset.	1	2	3	4
7. I am presently worrying over possible misfortunes.	1	2	3	4
8. I feel satisfied.	4	3	2	1
9. I feel frightened.	1	2	3	4
10. I feel comfortable.	4	3	2	1

11. I feel self-confident.	4	3	2	1
12. I feel nervous.	1	2	3	4
13. I am jittery.	1	2	3	4
14. I feel indecisive.	1	2	3	4
15. I am relaxed.	4	3	2	1
16. I feel content.	4	3	2	1
17. I am worried.	1	2	3	4
18. I feel confused.	1	2	3	4
19. I feel steady.	4	3	2	1
20. I feel pleasant.	4	3	2	1

STAI Form Y-2

DIRECTIONS: A number of statements which people have used to describe themselves are given below. Read each statement and then circle the appropriate number to the right of the statement to indicate how you generally feel. There are no right or wrong answers. Do not spend too much time on any one statement but give the answer which seems to describe how you generally feel.

Topic	Not at all	Somewhat	Moderately so	Very much so
	Please tick(√)			
21. I feel pleasant.	1	2	3	4
22. I feel nervous and restless.	1	2	3	4
23. I feel satisfied with myself.	4	3	2	1
24. I wish I could be as happy as others	4	3	2	1

seem to be.				
25. I feel like a failure.	1	2	3	4
26. I feel rested.	4	3	2	1
27. I am “calm, cool, and collected”	4	3	2	1
28. I feel that difficulties are piling up so that I cannot overcome them.	1	2	3	4
29. I worry too much over something that really doesn't matter.	1	2	3	4
30. I am happy .	4	3	2	1
30. I have disturbing thoughts.	1	2	3	4
31. I lack self-confidence.	1	2	3	4
32. I feel secure.	4	3	2	1
33. I make decisions easily.	4	3	2	1
35. I feel inadequate.	1	2	3	4
36. I am content.	4	3	2	1
37. Some unimportant thought runs through my mind and bothers me.	1	2	3	4
38. I take disappointments so keenly that I can’t put them out of my mind.	1	2	3	4
39. I am a steady person.	4	3	2	1
40. I get in a state of tension or turmoil as I think over my recent concerns and interests.	1	2	3	4

Appendix D (Chinese Version)

状态一特质焦虑问卷

编号：________________________ 性别：☐ 男 ☐ 女

年龄：__________________岁

状态焦虑问卷

指导语：下面列出的是一些人们常常用来描述他们自己的陈述，请阅读每一个陈述，然后在右边适当的圈上打勾来表示你现在最恰当的感觉，也就是你此时此刻最恰当的感觉。没有对或错的回答，不要对任何一个陈述花太多的时间去考虑，但所给的回答应该是你现在最恰当的感觉。

题目	完全没有	有些	中等程度	非常明显
	打勾(√)			
1. 我感到心情平静	1	2	3	4
2. 我感到安全	4	3	2	1
3. 我是紧张的	1	2	3	4
4. 我感到紧张束缚	1	2	3	4
5. 我感到安逸	4	3	2	1
6. 我感到烦乱	1	2	3	4
7. 我现在正烦恼，感到这种烦恼超过了可能的不幸	1	2	3	4
8. 我感到满意	4	3	2	1
9. 我感到害怕	1	2	3	4

10. 我感到舒适	4	3	2	1
11. 我有自信心	4	3	2	1
12. 我觉得神经过敏	1	2	3	4
13. 我极度紧张不安	1	2	3	4
14. 我优柔寡断	1	2	3	4
15. 我是轻松的	4	3	2	1
16. 我感到心满意足	4	3	2	1
17. 我是烦恼的	1	2	3	4
18. 我感到慌乱	1	2	3	4
19. 我感觉镇定	4	3	2	1
20. 我感到愉快	4	3	2	1

特质焦虑问卷

指导语：下面列出的是人们常常用来描述他们自己的一些陈述，请阅读每一个陈述后，然后在右边恰当约圈内打勾，来表示你经常的感觉。没有对或错的回答。不要对任何一个陈述花太多的时间去考虑，但所给均回答应该是你平常所感觉到的。

题目	完全没有	有些	中等程度	非常明显
	打勾（√）			
21. 我感到愉快	1	2	3	4
22. 感到神经过敏和不安	1	2	3	4
23. 我感到自我满足	4	3	2	1
24. 我希望能象别人那样高兴	4	3	2	1

25.我感到我象衰竭一样	1	2	3	4
26.我感到很宁静	4	3	2	1
27.我是平静的、冷静的和泰然自若的	4	3	2	1
28.我感到困难一一堆集起来，因此无法克服	1	2	3	4
29.我过分优虑一些事，实际这些事无关紧要	1	2	3	4
30.我是高兴的	4	3	2	1
31.我的思想处于混乱状态	1	2	3	4
32.我缺乏自信心	1	2	3	4
33.我感到安全	4	3	2	1
34.我容易做出决断	4	3	2	1
35.我感到不合适	1	2	3	4
36.我是满足的	4	3	2	1
37.一些不重要的思想总缠绕着我，并打扰我	1	2	3	4
38.我产生的沮丧是如此强烈，以致我不能从思想中排除它们	1	2	3	4
39.我是一个镇定的人	4	3	2	1
40.当我考虑我目前的事情和利益时，我就陷人紧张状态	1	2	3	4

Appendix E

Flow chart of the major stages of an EEG study

The main process of EEG research includes in: 1) study design, 2) equipment, software and laboratory setting, 3) acquisition parameters, 4) data collection monitoring procedures, 5) quality control procedures prior to data sharing, 6) parameters and algorithms used in data pre-processing, 7) EEG feature extraction algorithms, 8) the choice of statistical frameworks, 9) data archiving processes, and 10) the method of knowledge translation, see figure.

Electrode position map of the International 10-20 system for EEG (electroencephalogram) recording

Electroencephalogram (EEG) data acquisition equipment

the ActiCHamp amplifier with 64-channel active AgCl electrodes (actiCHamp Plus, Brain Products GmbH, Gilching, Germany)

Appendix F

Autonomic heart rate variability (HRV) data acquisition equipment

uBioMacpa v70 (BioSense Creative, Korea)

Appendix G

Physical Fitness data acquisition equipment

Body composition. It will be measured by body scale (Huawei Smart Scale 3 Pro, Huawei, China), The indexes of body scale are including: Body water, Protein level, Body fat, Muscle mass, Bone mineral content.

Physical Fitness. According to the different items of fitness test, different apparatus will be used to measure. For example, Height and Weight (Weight scale, Jiangsu Suhong Medical Equipment Co., Ltd., China), Cardiopulmonary function (JH-1662 Electronic Spirometer, Jiangsu Suhong Medical Equipment Co., Ltd., China), Muscle strength (JH-1881 Electronic Grip , Changzhou Jihao Electronics Co., Ltd., China), Flexibility (JH-1441 Mechanical flexionr, Jiangsu Suhong Medical Equipment Co., Ltd., China), Power of lower limbs (Simple long jump mat,, Jiangsu Suhong Medical Equipment Co., Ltd., China). In adddition, Balance test will be used One-leg standing test with eyes closed, and use a Sports stopwatch (RS-8060 STOPWATCH, RESEE, Shenzhen Resee Technology Co., Ltd.,Guangdong, China) to count the time.

Body scale (Huawei Smart Scale 3 Pro, Huawei, China)

Height and Weight (Weight scale, Jiangsu Suhong Medical Equipment Co., Ltd., China)

Power of lower limbs (Simple long jump mat,, Jiangsu Suhong Medical Equipment Co., Ltd., China)

Muscle strength (JH-1881 Electronic Grip , Changzhou Jihao Electronics Co., Ltd., China)

Flexibility (JH-1441 Mechanical flexionr, Jiangsu Suhong Medical Equipment Co., Ltd., China)

Cardiopulmonary function (JH-1662 Electronic Spirometer, Jiangsu Suhong Medical Equipment Co., Ltd., China),

Sports stopwatch (RS-8060 STOPWATCH, RESEE, Shenzhen Resee Technology Co., Ltd.,Guangdong, China)
