# Supplementary material for: Effects of Tai Chi on anxiety and theta oscillation power in college students during the COVID-19 pandemic: A randomized controlled trial
Source: PLoS One. 2024 Nov 1;19(11):e0312804. doi: 10.1371/journal.pone.0312804 (PMC11530040; doi:10.1371/journal.pone.0312804)
Supplement: S2 File — (PDF) [file pone.0312804.s002.pdf]

Number	Group	Age(Year)	Gender	High(cm)	Weigh(kg)	SAI (points)-pre	SAI (points)-post	TAI (points)-pre	TAI (points)-post
1	TC group	20	F	163.9	60.05	40	38	36	36
3	TC group	20	F	159.5	50.8	20	20	22	22
9	TC group	21	M	174	58.7	29	24	26	26
11	TC group	20	F	153.8	55.2	41	34	43	36
13	TC group	20	M	167.3	65.35	47	48	58	54
15	TC group	20	F	167.6	52.95	39	30	38	36
17	TC group	20	F	162.3	71.2	34	37	34	39
19	TC group	20	F	165.3	58.05	40	43	41	45
25	TC group	20	F	173.8	59.55	23	26	26	30
29	TC group	20	F	152.7	63.3	38	32	45	34
31	TC group	20	F	158.4	66.2	50	20	46	21
35	TC group	20	F	155.4	54.65	29	30	31	33
37	TC group	20	F	155.6	57.7	39	40	42	45
39	TC group	20	F	158.6	62.95	52	50	52	57
41	TC group	20	M	176.4	95.6	37	29	45	46
43	TC group	20	M	181	68.45	46	43	47	46
47	TC group	20	M	162	48.2	49	48	51	46
49	TC group	21	M	169.2	62.95	21	31	23	27
51	TC group	20	M	167.6	56.6	28	23	30	28
55	TC group	20	F	161.7	58.65	31	23	37	21
56	TC group	20	F	168	55.45	47	42	50	45
58	TC group	20	F	173	54.05	27	20	32	23
2	Control group	22	F	152.5	50.95	41	39	45	44
6	Control group	20	F	163.1	53.95	42	50	42	50
8	Control group	20	F	159.4	63.45	43	57	46	53
10	Control group	21	M	175.7	59.1	29	25	32	27
12	Control group	21	F	153.2	50	45	50	44	50
14	Control group	21	F	161.8	50.45	26	24	22	24
18	Control group	20	F	156.4	46.5	46	54	48	54
20	Control group	21	F	159.8	53.65	41	33	44	34
26	Control group	21	F	158.9	53.95	36	69	32	57
28	Control group	21	M	172	64.05	51	57	47	52
30	Control group	20	F	167.9	54.2	50	53	50	50
32	Control group	21	F	163.7	53.65	35	47	40	47
34	Control group	21	F	159.6	55.75	27	26	36	28
36	Control group	21	F	154.1	44	50	50	50	52
38	Control group	20	F	164	48.15	24	20	24	23
40	Control group	21	M	181.6	66.7	53	55	53	57
46	Control group	21	F	166.8	52.55	39	50	44	47
48	Control group	21	F	146.9	46.3	21	31	22	30
50	Control group	20	F	169.9	77.5	30	29	30	37
52	Control group	21	F	155.9	83.95	38	34	41	35
57	Control group	21	F	161.7	53.35	50	53	44	50
59	Control group	20	F	158.5	39.85	35	42	35	40
60	Control group	20	F	167.8	59.75	33	35	37	42

STAI (points)-pre	STAI (points)-post	C3-θ(μ VHz)-pre	C3-θ(μ VHz)-post	C4-θ(μ VHz)-pre	C4-θ(μ VHz)-post	F3-θ(μ VHz)-pre	F3-θ(μ VHz)-post	F4-θ(μ VHz)-pre
76	74	0.002523	0.003915	0.004798	0.003833	0.001849	0.003815	0.002691
42	42	0.003474	0.004339	0.002945	0.002827	0.004263	0.004793	0.004716
55	50	0.005183	0.002621	0.005302	0.002492	0.005424	0.00382	0.004956
84	70	0.004771	0.00189	0.005283	0.003145	0.003716	0.002981	0.005761
105	102	0.003628	0.003223	0.006163	0.002402	0.005621	0.003007	0.006937
77	66	0.001744	0.002077	0.001752	0.003399	0.001619	0.001294	0.002551
68	76	0.002032	0.003624	0.001882	0.002268	0.001711	0.00572	0.001404
81	88	0.003566	0.005327	0.003954	0.003621	0.002855	0.00628	0.003778
49	56	0.011505	0.019529	0.016467	0.023673	0.013841	0.018369	0.010389
83	66	0.017612	0.006411	0.015553	0.007449	0.015618	0.005582	0.009589
96	41	0.006674	0.027745	0.011641	0.030979	0.008493	0.024282	0.009114
60	63	0.005739	0.006523	0.002734	0.008053	0.007167	0.005565	0.004911
81	85	0.002443	0.003451	0.002956	0.003596	0.001756	0.003767	0.002187
104	107	0.020413	0.049228	0.020941	0.049235	0.012175	0.039308	0.0118
82	75	0.001846	0.003706	0.001757	0.0026	0.001528	0.004481	0.001616
93	89	0.002896	0.001504	0.002668	0.00174	0.001957	0.001676	0.001341
100	94	0.004399	0.002409	0.002611	0.003357	0.004332	0.002247	0.001574
44	58	0.004697	0.002163	0.003262	0.002471	0.005286	0.003375	0.003818
58	51	0.001894	0.004169	0.002621	0.004256	0.001588	0.004432	0.002056
68	44	0.012043	0.02039	0.007685	0.017021	0.005767	0.017183	0.006068
97	87	0.004559	0.027833	0.004335	0.038194	0.004879	0.019071	0.004895
59	43	0.00411	0.006188	0.003675	0.005837	0.003345	0.005043	0.00274
86	83	0.003819	0.001335	0.006339	0.004544	0.004213	0.002755	0.006211
84	100	0.00403	0.004688	0.003716	0.00307	0.004018	0.004399	0.004189
89	110	0.02686	0.003961	0.027206	0.004237	0.036311	0.0074	0.034543
61	52	0.002232	0.002591	0.001915	0.002206	0.005157	0.002269	0.00176
89	100	0.00479	0.003027	0.004049	0.002852	0.00433	0.001697	0.003084
48	48	0.004735	0.00237	0.004728	0.004062	0.00501	0.003895	0.003332
94	108	0.00618	0.004511	0.004741	0.00535	0.005063	0.002976	0.003978
85	67	0.003499	0.004856	0.004103	0.005898	0.004739	0.002771	0.006269
68	126	0.005984	0.003522	0.00883	0.002615	0.004909	0.004887	0.005323
98	109	0.003565	0.00257	0.006293	0.001203	0.003738	0.001919	0.002937
100	103	0.005279	0.005254	0.004258	0.003479	0.004951	0.004334	0.003038
75	94	0.003737	0.004032	0.002762	0.003208	0.002351	0.002849	0.00166
63	54	0.003819	0.002561	0.003801	0.002414	0.004257	0.002622	0.004241
100	102	0.006207	0.002003	0.004734	0.003115	0.006076	0.002522	0.002695
48	43	0.005268	0.004271	0.00386	0.001574	0.007098	0.002414	0.005514
106	112	0.001217	0.001126	0.00157	0.001181	0.001345	0.001319	0.001532
83	97	0.012927	0.009773	0.011875	0.010267	0.009466	0.010866	0.010423
43	61	0.001909	0.004873	0.001691	0.002479	0.003773	0.00325	0.002656
60	66	0.003078	0.002783	0.00528	0.003685	0.001968	0.004161	0.003338
79	69	0.009441	0.005325	0.013398	0.00713	0.006073	0.006775	0.009969
94	103	0.005092	0.003549	0.006699	0.004469	0.005382	0.002583	0.004563
70	82	0.004024	0.004593	0.003477	0.004002	0.003594	0.003594	0.002065
70	77	0.010658	0.007204	0.00889	0.005285	0.009856	0.006705	0.008429

F4-θ(μ VHz)-post	P3-θ(μ VHz)-pre	P3-θ(μ VHz)-post	P4-θ(μ VHz)-pre	P4-θ(μ VHz)-post	T7-θ(μ VHz)-pre	T7-θ(μ VHz)-post	T8-θ(μ VHz)-pre
0.003286	0.003521	0.003626	0.005632	0.002226	0.002058	0.002427	0.002965
0.002458	0.002155	0.003331	0.002269	0.002011	0.002309	0.004011	0.000762
0.004026	0.006076	0.002501	0.005765	0.002449	0.003252	0.002315	0.003804
0.002562	0.00683	0.003928	0.006488	0.00319	0.001877	0.001523	0.004077
0.004342	0.00446	0.005538	0.006362	0.003108	0.002553	0.002421	0.005548
0.003239	0.001523	0.004326	0.001894	0.004753	0.00115	0.002234	0.001861
0.007647	0.002216	0.002882	0.003698	0.002707	0.00188	0.002113	0.001083
0.003619	0.003903	0.00863	0.004768	0.005158	0.001586	0.003126	0.002338
0.0221	0.013023	0.017599	0.016953	0.026226	0.009817	0.019505	0.012885
0.007531	0.022256	0.007034	0.018019	0.011265	0.014139	0.003051	0.008616
0.031494	0.005295	0.024509	0.010584	0.033981	0.003374	0.011883	0.007455
0.006316	0.006478	0.006986	0.004413	0.007861	0.003314	0.004228	0.001388
0.003805	0.003366	0.004096	0.004164	0.003392	0.002358	0.002455	0.00134
0.02845	0.027292	0.06535	0.023536	0.051292	0.012792	0.021413	0.008356
0.002461	0.001542	0.002038	0.001388	0.001334	0.001354	0.001698	0.000848
0.002429	0.004146	0.001942	0.004743	0.002392	0.001282	0.000554	0.001434
0.003474	0.00424	0.004073	0.002828	0.004804	0.002239	0.002415	0.001342
0.003119	0.004744	0.002464	0.002924	0.00296	0.005382	0.001742	0.003805
0.004927	0.002354	0.005481	0.002484	0.004901	0.001137	0.002766	0.001668
0.025813	0.014751	0.016345	0.018986	0.013399	0.008854	0.013272	0.004732
0.022887	0.005844	0.058316	0.00488	0.052453	0.004396	0.045363	0.003011
0.004074	0.006209	0.010182	0.006035	0.00898	0.00237	0.00464	0.003045
0.00308	0.004212	0.001439	0.005465	0.002053	0.001962	0.001111	0.003195
0.002694	0.003149	0.004325	0.00382	0.003268	0.001755	0.001679	0.002602
0.010063	0.016557	0.004644	0.020127	0.004566	0.01789	0.004853	0.0142
0.001909	0.00469	0.003729	0.004318	0.002886	0.002181	0.001611	0.001889
0.001924	0.00369	0.003178	0.003619	0.00322	0.003136	0.002297	0.001149
0.003539	0.003908	0.002183	0.003559	0.003624	0.001462	0.001635	0.00313
0.004189	0.004282	0.004986	0.00534	0.005121	0.003324	0.002886	0.003085
0.005681	0.002772	0.005163	0.004877	0.005964	0.002031	0.001885	0.00413
0.00208	0.007391	0.00362	0.014955	0.002497	0.005666	0.004255	0.00575
0.001152	0.00742	0.00442	0.006227	0.003072	0.00225	0.000884	0.003782
0.003544	0.00466	0.004586	0.003989	0.004917	0.003977	0.002661	0.001604
0.002381	0.003669	0.005494	0.002757	0.004449	0.002195	0.001985	0.003491
0.003527	0.003367	0.002854	0.005365	0.002249	0.000729	0.00233	0.002838
0.003702	0.007745	0.004405	0.006571	0.00521	0.003811	0.002541	0.001959
0.002075	0.005645	0.005951	0.004696	0.003387	0.004348	0.005094	0.002301
0.001319	0.001196	0.001153	0.0016	0.001347	0.001337	0.000649	0.001361
0.006493	0.019301	0.008352	0.014258	0.007415	0.014972	0.006327	0.009848
0.001917	0.00216	0.003209	0.002054	0.002425	0.001656	0.001192	0.003031
0.00326	0.00586	0.002861	0.005599	0.003731	0.001974	0.003545	0.003124
0.007874	0.020404	0.006493	0.021948	0.006321	0.007184	0.003089	0.014923
0.003503	0.004587	0.007995	0.006109	0.006727	0.010326	0.002053	0.007502
0.003347	0.002864	0.006197	0.00363	0.004234	0.002427	0.002321	0.002258
0.006057	0.012224	0.007694	0.013412	0.007068	0.005038	0.00306	0.003971

T8-θ(μ VHz)-post
0.00319
0.000905
0.001369
0.00114
0.002238
0.002465
0.002578
0.004361
0.016614
0.005188
0.012511
0.005178
0.001937
0.023765
0.001388
0.001327
0.001665
0.001675
0.003232
0.01138
0.051387
0.002912
0.002093
0.001235
0.004831
0.002041
0.001933
0.002035
0.003295
0.002351
0.001015
0.000489
0.003766
0.001285
0.001939
0.001814
0.001324
0.001471
0.004841
0.001916
0.002056
0.003505
0.002583
0.001525
0.00206
