# Supplementary material for: Effects of Tai Chi on anxiety and theta oscillation power in college students during the COVID-19 pandemic: A randomized controlled trial
Source: PLoS One. 2024 Nov 1;19(11):e0312804. doi: 10.1371/journal.pone.0312804 (PMC11530040; doi:10.1371/journal.pone.0312804)
Supplement: S3 File — (PDF) [file pone.0312804.s003.pdf]

Inclusivity in global research

PLOS' policy on inclusivity in global research aims to improve transparency in the reporting of research performed outside of researchers' own country or community and ensures that PLOS publications reporting global research adhere to high standards for research ethics and authorship. Authors of relevant research articles may be asked to complete the questionnaire below, which outlines ethical, cultural, and scientific considerations specific to inclusivity in global research. This questionnaire may be requested when researchers have travelled to a different country to conduct research, if research uses samples collected in another country, research with Indigenous populations or their lands, or if research is on cultural artefacts. Researchers travelling to another country solely to use laboratory equipment will not normally be required to complete the questionnaire. However, the questionnaire can be requested at the journal's discretion for any submission – if you have been requested to complete this questionnaire by the PLOS journal you submitted to, please do so.

Please complete the questionnaire below and include this as a Supporting Information file with your manuscript. Note that if your paper is accepted for publication, this checklist will be published with your article in the supporting information files. Please ensure that you reference the checklist in the main body of your manuscript. We suggest adding a subsection 'Inclusivity in global research' to your Methods section and adding the following sentence: "Additional information regarding the ethical, cultural, and scientific considerations specific to inclusivity in global research is included in the Supporting Information (SX Checklist)"

The questions have been designed to be applicable to a wide range of study types, and there are subsections for both human subjects research and non-human subjects research. If any of the questions are not relevant to your research please mark them as "N/A" as appropriate.

Ethical considerations, permits and authorship

This section is applicable to all research types.

Provide details as to who granted permissions and/or consent for the study to take place in the Methods section of your manuscript. This should include the names of **all** ethics boards, governmental organizations, community leaders or other bodies that provided approval for the study. If individuals provided approval refer to these people by their role or title but do not list their name(s).

Reported on page number: Page 6, Line 125-126

If there were any deviations from the study protocol after approval was obtained please provide details of these changes in the Methods section of your manuscript.

Reported on page number: Page 5, Line 127

Did this study involve local collaborators that are residents of the country where the research was conducted or members of the community studied? If you do not have any authors from said communities, please provide an explanation for this below.

Everyone listed as an author meet PLOS' criteria for authorship and all individuals who meet these criteria were included in the author byline, see title Page 1, Line 4-7.

Human subjects research (e.g. health research, medical research, cross-cultural psychology)

Did you obtain written informed consent from a representative of the local community or region before the research took place? How did you establish who speaks for the community? Details of written informed consent obtained from study participants should be reported separately in the Methods section of your manuscript.

Yes, we obtained written informed consent from a representative of the local community before the research took place. To establish who speaks for the community, we followed a multi-step process. First, we conducted extensive consultations with local leaders, including elected officials, community elders, and representatives of community-based organizations. Through these discussions, we identified a group of individuals who were widely recognized and respected within the community as having the authority to represent the interests and concerns of the community as a whole.

We then held a series of meetings to explain the purpose and nature of the research, answer questions, and seek input and feedback from community members. At these meetings, the proposed representative was introduced and given an opportunity to address the community and answer questions. After these consultations and discussions, a consensus was reached among community members that the identified representative had the necessary legitimacy and authority to represent the community in matters related to the research.

In addition to obtaining consent from the community representative, we also obtained written informed consent from each individual study participant in accordance with ethical guidelines. Details of this process are reported separately in the Methods section of our manuscript, see Page 6, Line 124.

How did members of the local community provide input on the aims of the research investigation, its methodology, and its anticipated outcome(s)?

First, we held a series of meetings. At these meetings, we presented an overview of the proposed research, including its broad aims and potential methodologies. Community members were then given the opportunity to ask questions, express concerns, and offer suggestions. This dialogue allowed us to refine our research aims to better address the community's priorities and interests. For example, based on community feedback, we adjusted our focus to include the issue of psychological anxiety, which is of great concern to local residents.

Second, we formed a advisory board consisting of representatives from various sectors of the community, including local business owners, educators, and community activists. This board met regularly throughout the research process and provided input on the methodology. They offered insights on the most appropriate data collection methods, potential sources of bias, and ways to ensure the research was culturally sensitive and relevant to the local context.

Finally, we distributed written questionnaires and conducted individual interviews with a diverse sample of community members. These interactions allowed us to gather specific feedback on the anticipated outcomes. Community members provided valuable perspectives on what they hoped to see as a result of the research, which helped us shape our interpretation and dissemination plans. For instance, some community members have expressed a desire to expand the scope of the study to address the health challenges of local older adults, among other things.

When engaging with the local community, how did you ensure that the informed consent documents and other materials could be understood by local stakeholders?

When engaging with the local community, we took several steps to ensure that the informed consent documents and other materials could be understood by local stakeholders.

First, we conducted a thorough review of the materials with local language experts. These experts were fluent in the languages spoken in the community and had a deep understanding of the local culture and context. They helped us translate the documents into the local language(s) and ensured that the translations were accurate and idiomatic.

Second, we held focus group discussions with a diverse group of community members to test the comprehensibility of the materials. In these discussions, we asked participants to read through the informed consent documents and other materials and provide feedback on their clarity and understandability. Based on their feedback, we made revisions to the materials to address any areas of confusion or ambiguity.

Third, we used simple and straightforward language in the materials. We avoided technical jargon and complex legal terms, and instead used plain language that was accessible to a wide range of readers. We also included visual aids, such as diagrams and illustrations, to help explain key concepts and procedures.

Finally, we provided opportunities for community members to ask questions and seek clarification about the materials. We had trained staff available to answer questions and provide additional explanations as needed. This ensured that community members had a full understanding of the research and were able to make an informed decision about whether to participate.

Will the findings of the research be made available in an understandable format to stakeholders in the community where the study was conducted (e.g. via a presentation, summary report, copies of publications, etc.)? Please provide details of how this will be achieved.

Yes, the findings of the research will be made available in an understandable format to stakeholders in the community where the study was conducted. We are committed to ensuring that the results are accessible and useful to the local community.

To achieve this, we plan to take the following steps:

Presentation: We will organize a community presentation where the research team will present the key findings in an accessible language. Visual aids such as charts and graphs will be used to help illustrate the results. This presentation will be open to all community members and will be held at a convenient location and time.

Summary report: A summary report will be prepared that highlights the main findings of the research. The report will be written in plain language and will avoid technical jargon. Copies of the report will be distributed to community centers, local libraries, and other public places where community members can access them.

Copies of publications: If the research is published in academic journals or other publications, we will make electronic or paper version copies available to the community. We will also provide a lay summary of the publication that explains the key findings in an accessible way.

We believe that by making the research findings available in these ways, we can ensure that the community is informed about the results and can use the information to make decisions and take actions that benefit their lives.

Non-human subjects research using specimens/ animals collected as part of the study, or those housed in archival collections. Examples include archaeology, paleontology, botany and zoology.

Did the permission you obtained from a local authority to perform the study include an agreement on access to outputs and benefit sharing? This may include procedures to enable fair distribution of the benefits and resources arising from the research performed. Please include any details of Prior Informed Consent and Benefit Sharing Agreements obtained. These may be required by field-specific regulations, for example the Convention on Biological Diversity (CBD) and the associated Nagoya Protocol.

If the material used in your study was imported, please A) provide the year it was imported and B) indicate whether permits were obtained to import/export the materials used, C) provide details of any permits obtained. If this information is not available, please indicate this.

If you used archival specimens, please state how the material used in your study was acquired by the institute it is held in and provide details of any permits obtained for the original excavations/ sample collection. If this

information is not available, please indicate this.

How was the potential cultural significance of the materials collected in your study to local communities considered in your research design? Were Indigenous peoples and/or local researchers and institutions involved with archaeological excavations / collection of specimens? If so, please provide a description of their involvement.

If your manuscript includes photographs of human remains please indicate whether authors obtained permission from descendants or affiliated cultural communities to do so.
