# Supplementary material for: Effects of Tai Chi on anxiety and theta oscillation power in college students during the COVID-19 pandemic: A randomized controlled trial
Source: PLoS One. 2024 Nov 1;19(11):e0312804. doi: 10.1371/journal.pone.0312804 (PMC11530040; doi:10.1371/journal.pone.0312804)
Supplement: Attachment — Submitted filename: Response to Reviewers.docx [file pone.0312804.s004.docx]

**Letter from Authors to academic editor and reviewers**

12 Sep. 2024

Dear Academic Editor of **PLOS ONE**

Firstly, we greatly appreciated the support from Editor of **PLOS ONE** and thank you very much for considering our manuscript entitled ***“*Effects of Tai Chi on anxiety and theta oscillation power in college students during the COVID-19 pandemic: a randomized controlled trial”** and permitting the revision. We are very much pleased with the results and also appreciate for kind help of you with submitting valuable comments.

Secondly, we would like to inform you that we have carefully considered and revised the manuscript according to the reviewers’ comments in detail. We have adopted all the suggestions in the revised manuscript; our point-by-point responses to comments are detailed on the following pages.

To facilitate the reading, our answers are arranged in the form of a list from the reviewers. Each item consists of a relevant fragment of the reviewers’comment, followed by our response written in “blue color”.

For editor's comments, we highlighted all revisions in "green color" in the attached text file, for reviewer 1's comments, we highlighted all revisions in "purple color",

for reviewer 2's comments, we highlighted all revisions in "yellow color",

and for reviewer 3's comment, We express our gratitude.

Additionally, page and line number information for all changes above were noted in this cover letter. We think that the manuscript has been greatly improved by these revisions. We would greatly appreciate if you could consider our manuscript to publish as Original Article in PLOS ONE.

Finally, we would also like to take this opportunity to thank all reviewers for their valuable comments and suggestions, which helped to considerably improve the quality of the manuscript.

Sincerely Yours,

Corresponding Author:Min Wang

**Journal requirements**

1. When submitting your revision, we need you to address these additional requirements.Please ensure that your manuscript meets PLOS ONE's style requirements, including those for file naming.

Authors: We have revised some additional requirements to meets PLOS ONE style, including file naming.

2. Please include a complete copy of PLOS’ questionnaire on inclusivity in global research in your revised manuscript.

Authors: We have included a complete copy of PLOS’ questionnaire on inclusivity in global research in your revised manuscript, including added contexts, as follows:

There were no deviations from the study protocol after approval was obtained. The research was conducted strictly in accordance with the approved plan, and no changes were made to the methods, procedures, or design throughout the study. Additional information regarding the ethical, cultural, and scientific considerations specific to inclusivity in global research is included in the Supporting Information.(Page 6, Line 127; Page 7, Line 128-131)

S1 File. Inclusivity in global research questionnaire. (Page 28, Line 630)

3. Thank you for stating the following financial disclosure:

 [This work was supported by the Zhejiang Province Philosophy and Social Sciences Leading Talent Cultivation Project (No. 23YJRC15ZD).].

Please state what role the funders took in the study.  If the funders had no role, please state: ""The funders had no role in study design, data collection and analysis, decision to publish, or preparation of the manuscript."" If this statement is not correct you must amend it as needed.

Please include this amended Role of Funder statement in your cover letter; we will change the online submission form on your behalf.

Authors: We have restated role in financial disclosure and , as follow: “This work was supported by the Zhejiang Province Philosophy and Social Sciences Leading Talent

Cultivation Project (No. 23YJRC15ZD), and the funders had no role in study design, data collection and analysis, decision to publish, or preparation of the manuscript.”

4. Thank you for stating the following in the Acknowledgments Section of your manuscript:

[We would like to thank all research assistants who helped us conduct this study and all volunteers that participated in it, and the publication of this article: the Major Cultivation Project of Leading Talents in Philosophy and Social Sciences in Zhejiang Province (Project Number: 23YJRC15ZD), China.]

We note that you have provided funding information that is not currently declared in your Funding Statement. However, funding information should not appear in the Acknowledgments section or other areas of your manuscript. We will only publish funding information present in the Funding Statement section of the online submission form.

Please remove any funding-related text from the manuscript and let us know how you would like to update your Funding Statement. Currently, your Funding Statement reads as follows:

 [This work was supported by the Zhejiang Province Philosophy and Social Sciences Leading Talent Cultivation Project (No. 23YJRC15ZD).].

Please include your amended statements within your cover letter; we will change the online submission form on your behalf.

Authors:We have removed the fund from the acknowledgements section of the manuscript and added a statement of the funder's role to the cover letter. (see Page 24, Line 473-474)

5. In the online submission form, you indicated that [If someone need raw data such as EEG, can contact the first author of this article by email.]. All PLOS journals now require all data underlying the findings described in their manuscript to be freely available to other researchers, either 1. In a public repository, 2. Within the manuscript itself, or 3. Uploaded as supplementary information.

This policy applies to all data except where public deposition would breach compliance with the protocol approved by your research ethics board. If your data cannot be made publicly available for ethical or legal reasons (e.g., public availability would compromise patient privacy), please explain your reasons on resubmission and your exemption request will be escalated for approval.

 Authors:We have uploaded raw data, see supplementary information.

S5 File. Raw data of study. (Page 29, Line 634)

6. Please amend your list of authors on the manuscript to ensure that each author is linked to an affiliation. Authors’ affiliations should reflect the institution where the work was done (if authors moved subsequently, you can also list the new affiliation stating “current affiliation:….” as necessary).

Authors:We have added the title page, and list all authors’ affiliations, see page 1.

7. Please include captions for your Supporting Information files at the end of your manuscript, and update any in-text citations to match accordingly. Please see our Supporting Information guidelines for more information: http://journals.plos.org/plosone/s/supporting-information.

Authors: According to the order of the support information files, we have added the corresponding file titles, and update any figure citations to match accordingly.

Supporting information

S1 Fig. Flowchart of the study protocol.

S1 Table. Subject characteristics

S2 Table. Effects of Tai Chi exercise on anxiety of college students.

S3 Table. Effects of Tai Chi exercise on theta oscillatory power of college students

S4 Table. Correlation between HRV and Cognitive Performance Variables.

S1 File. Clinical Trial Protocol.

S2 File. EC application form.

S3 File.Written consent form

S4 File. EC approval document

S5 File. Raw data of study.

S6 File. Inclusivity in global research questionnaire.

(Page 28, Line 624-631; Page 29, Line 632-635)

**Reviewers' comments**

Reviewer #1:

1.What test and what effect size were used for sample size calculation? For such a small sample size, it is almost impossible to stratify the random sample over sex, age and BMI groups. The authors did not mention how many age groups stratified. Assume there are 3 age groups, there will be 2x3x5=30 strata to divide the total N=60. If the sample was stratified by age, why is there significant difference between the two treatment arms as shown in Table 1? Was age adjusted in the primary analysis?

Authors: We have revised the relevant content of sample size calculation, as follows:

For the sample size calculation, we used t-test by G*Power to ensure that an effect of 0.8 was obtained with α = 0.05 and effect size of 0.85, requiring a minimum of 22 samples per group^[10]^. The choice of test and effect size was based on our research question and prior knowledge in the field^[11,12]^. (Page 5, Line 94-97)

10.	Faul F, Erdfelder E, Buchner A, Lang A-G. Statistical power analyses using G* Power 3.1: Tests for correlation and regression analyses. Behavior research methods. 2009;41(4):1149-60. doi.org/10.3758/BRM.41.4.1149

11.	Li X, Geng J, Du X, Si H, Wang Z. Relationship between the practice of tai chi for more than 6 months with mental health and brain in university students: an exploratory study. Frontiers in Human Neuroscience. 2022;16:912276. [doi.org/10.3389/fnhum.2022.912276](https://doi.org/10.3389/fnhum.2022.912276)

12.	Henz D, Schöllhorn WI. Temporal courses in EEG theta and alpha activity in the dynamic health Qigong techniques Wu Qin Xi and Liu Zi Jue. Frontiers in psychology. 2018;8:2291. [doi.org/10.3389/fpsyg.2017.02291](https://doi.org/10.3389/fpsyg.2017.02291)

Regarding stratification, although the sample size is relatively small, we did consider stratifying by certain factors to enhance the representativeness and internal validity of our study. However, as you correctly point out, with a sample size of 60, strict stratification over sex, age, and BMI groups becomes challenging. In practice, we did not perform a full stratification as described, while we did consider age as an important factor, we did not divide the sample into three distinct age groups for stratification purposes.

As for the significant difference between the two treatment arms shown in Table 1, this may be due to several reasons. Firstly, although age was not strictly stratified, it does not necessarily mean that age was not accounted for in the analysis. In our primary analysis, we adjusted for potential confounding factors including age using appropriate statistical methods, such as analysis with age as a covariate. Additionally, other factors such as differences in baseline characteristics between the treatment arms or the specific nature of the intervention could also contribute to the observed differences. In conclusion, while our sample size limited our ability to perform extensive stratification, we took steps in the analysis to account for important factors like age to ensure the validity of our results.

2.Tables 2 and 3, better add pre-post change for each group. In addition, better add p values for within-group and between-group comparisons. The F values and p values associated with the F tests are not friendly to general audience and can be omitted. There are two many outcomes for a limited sample size. The p values better be adjusted for multiple outcomes and correlations.

Authors: We appreciate the valuable feedback on our tables. We have added the pre-post change for Tables 2 and 3. This will provide a clearer understanding of the impact of the intervention within each group over time. Inaddition, we also have added the p values for within-group and between-group comparisons. This will help to highlight the statistical significance of the changes observed and the differences between the groups.

As for the f-values and P-values associated with the F-test, which are not friendly to the general reader, we understand the concern for accessibility for the general audience, so we removed these values as suggested, leaving only a set of values with a significant interaction effect of "group * time". To address this, we adjust the p values for multiple outcomes and correlations, as following Tables 2 and 3. We use an appropriate method such as the Bonferroni correction or false discovery rate control to account for the multiple comparisons and ensure that the results are not due to chance.

We believe that these changes will improve the clarity and interpretability of our tables and make our results more accessible to a wider audience, see Page 11, Line 221-222; Page 13, Line 257-261; Page 15, Line 283-285.

**Table 2. Effects of Tai Chi exercise on anxiety of college students**

| Variables | Tai Chi group (n=22) | | | Control group (n=23) | | | Difference before and after (M±SD) | | Between-group comparisons | Group*Time | |
| --- | --- | --- | --- | --- | --- | --- | --- | --- | --- | --- | --- |
|  | Pre (M±SD) | Post (M±SD) | P | Pre (M±SD) | Post (M±SD) | P | Tai Chi group | Control group | P | F | P |
| S-AI (points) | 36.68±9.65 | 33.23±9.72, | 0.04**^*^** | 38.48±9.38 | 42.74±13.23 | 0.03**^*^** | -3.45±7.57 | 4.26±8.58 | 0.01**^#^** | 10.20 | 0.01 |
| T-AI (points) | 38.86±10.06 | 36.18±10.75 | 0.10 | 39.48±9.01 | 42.74±10.88 | 0.04**^*^** | -2.68±7.43 | 3.26±7.07 | 0.03**^#^** | 7.56 | 0.01 |
| STAI (points) | 75.55±19.36 | 69.41±20.06 | 0.06 | 77.96±18.07 | 85.48±23.86 | 0.03**^*^** | -6.14±14.33 | 7.52±15.16 | 0.02**^#^** | 9.62 | 0.01 |

Abbreviations: S-AI = State Anxiety Inventory, T-AI = Trait Anxiety Inventory, STAI = State Trait Anxiety Inventory.

Note: * means significant within-group; ^#^ means significant between-group comparisons after the intervention.

**Table 3. Effects of Tai Chi exercise on theta oscillatory power of college students**

| Variables (10^-2^·μV·Hz) | Tai Chi group (n=22) | | | Control group (n=23) | | | Difference before and after (M±SD) | | Between-group comparisons | Group*Time | |
| --- | --- | --- | --- | --- | --- | --- | --- | --- | --- | --- | --- |
|  | Pre (M±SD) | Post (M±SD) | p | Pre (M±SD) | Post (M±SD) | p | Tai Chi group | Control group | P | F | P |
| C3-θ | 0.58±0.51 | 0.95±1.21 | 0.08 | 0.60±0.53 | 0.39±0.19 | 0.06 | 0.37±0.93 | -0.21±0.49 | 0.30 | 6.74 | 0.01 |
| C4-θ | 0.60±0.53 | 1.01±1.33 | 0.07 | 0.63±0.55 | 0.38±0.20 | 0.03* | 0.42±1.03 | -0.24±0.50 | 0.29 | 7.62 | 0.01 |
| F3-θ | 0.52±0.41 | 0.85±0.94 | 0.06 | 0.62±0.69 | 0.39±0.22 | 0.07 | 0.32±0.78 | -0.24±0.60 | 0.03**^#^** | 7.33 | 0.01 |
| F4-θ | 0.48±0.31 | 0.91±0.97 | 0.02* | 0.57±0.68 | 0.37±0.22 | 0.07 | 0.43±0.79 | -0..0±0.51 | 0.02**^#^** | 10.35 | 0.00 |
| P3-θ | 0.69±0.67 | 1.19±1.72 | 0.12 | 0.66±0.54 | 0.46±0.20 | 0.04* | 0.50±1.44 | -0.20±0.46 | 0.27 | 4.87 | 0.03 |
| P4-θ | 0.72±0.63 | 1.14±1.54 | 0.13 | 0.71±0.57 | 0.42±0.17 | 0.01* | 0.42±1.28 | -0.30±0.51 | 0.38 | 6.17 | 0.02 |
| T7-θ | 0.41±0.38 | 0.71±1.03 | 0.15 | 0.44±0.44 | 0.26±0.14 | 0.02* | 0.30±0.95 | -0.18±0.35 | 0.11 | 5.13 | 0.03 |
| T8-θ | 0.37±0.31 | 0.72±1.15 | 0.14 | 0.44±0.38 | 0.22±0.11 | 0.01* | 0.35±1.08 | -0.22±0.32 | 0.08 | 5.69 | 0.02 |

Note: * means significant within-group; ^#^ means significant between-group comparisons after the intervention.

Minor concerns:

3.Specify the decimal numbers for all p values <0.05.

Authors: We have specified decimal numbers for all p-values <0.05, see Page 12, Line 244; Page 13, Line 245-247, 251, 253, 255; Page 14, Line 268, 270-279.

4.Line 85 There are only 4 BMI groups instead 5 groups.

Authors: We have modified the BMI for only 4 groups, see Page 5, Line 100.

5.Table 1 omit the column for 95% CI.

Authors: We have deleted the column for 95% CI in Table 1, see Page 12, Line 239-241.

**Table 1. Subject characteristics (n=45)**

| Variables | Tai Chi group  (n=22) | Control group  (n=23) | P value |
| --- | --- | --- | --- |
| Age (years) | 20.09±0.29 | 20.70±0.56 | P＜0.05 |
| Gender (male, female) | 7, 15 | 3, 20 | 0.14 |
| Height (cm) | 164.87±7.73 | 162.23±7.97 | 0.87 |
| Weight (kg) | 60.75±9.64 | 55.73±10.19 | 0.64 |
| S-AI (points) | 36.68±9.65 | 38.48±9.38 | 0.52 |
| T-AI (points) | 38.86±10.06 | 39.48±9.01 | 0.83 |
| STAI (points) | 75.55±19.36 | 77.96±18.07 | 0.55 |
| C3-θ (10^-2^·μV·Hz) | 0.58±0.51 | 0.60±0.53 | 0.47 |
| C4-θ (10^-2^·μV·Hz) | 0.60±0.53 | 0.63±0.55 | 0.41 |
| F3-θ (10^-2^·μV·Hz) | 0.52±0.41 | 0.62±0.69 | 0.45 |
| F4-θ (10^-2^·μV·Hz) | 0.48±0.31 | 0.57±0.68 | 0.80 |
| P3-θ (10^-2^·μV·Hz) | 0.69±0.67 | 0.66±0.54 | 0.93 |
| P4-θ (10^-2^·μV·Hz) | 0.72±0.63 | 0.71±0.57 | 0.91 |
| T7-θ (10^-2^·μV·Hz) | 0.41±0.38 | 0.44±0.44 | 0.73 |
| T8-θ (10^-2^·μV·Hz) | 0.37±0.31 | 0.44±0.38 | 0.35 |

Abbreviations: S-AI = State Anxiety Inventory, T-AI = Trait Anxiety Inventory, STAI = State Trait Anxiety Inventory.

6.Limitation of the sample size should be discussed.

Authors: We have added discussion about limitation of the sample size, as follows:

First, a small sample size can limit the generalizability of our findings. Our results may not be representative of the broader population from which our sample was drawn, or they may not hold true in different settings or for different subgroups. Another concern with a small sample size is the increased risk of sampling bias. Despite these limitations, we took several steps to mitigate the impact of the small sample size. We used rigorous study design and statistical methods to ensure that our analyses were as accurate and reliable as possible. We also carefully considered the potential sources of bias and took steps to minimize them. Future research with larger sample sizes would be valuable to confirm and extend our findings. (Page 22, Line 429-432; Page 23, Line 433-438)

Reviewer #2:

1. The relationship between the COVID-19 pandemic and the trial requires further elaboration. Specifically, was the pandemic ongoing during the study? If so, how did you measure its impact on the participants and the outcomes? Furthermore, how were the participants affected by the pandemic, both psychologically and behaviorally?

Authors: Yes, the COVID-19 pandemic was ongoing during the study, and it had some impact on our trial.To measure its impact on the participants and the outcomes, we took several steps.

We regularly monitored the local and national COVID-19 situation, including infection rates and public health guidelines. We also asked participants about any changes in their living situations, access to healthcare, and adherence to study protocols due to the pandemic. Additionally, we collected data on any disruptions in their daily lives that could potentially affect the trial outcomes, such as completing the International Physical Activity Questionnaire (IPAQ) and Physical Activity Readiness Questionnaire (PARQ) twice, providing essential information on demographic data, medical history, and health behaviors (Page 7, Line 134-136).

In terms of psychological effects, we administered questionnaires to assess levels of stress and anxiety (Page 8, Line 162-163). We found that many participants reported increased levels of psychological distress due to the uncertainty and isolation brought on by the pandemic, see Table 2 (Page 13, Line 257-261). We also noted that some participants had difficulties in adhering to the study requirements due to lockdowns and restrictions on movement. Nevertheless, despite these adversities, many students exhibited resilience and adaptability by proactively seeking support from mental health resources while reinforcing their motivation to engage in trials (Page 23, Line 447-449).

To address these challenges, we provided additional support to the participants, such as online counseling services and flexible study schedules (Page 7, Line 140-141). We also adjusted our data collection methods to accommodate the changing circumstances. Additionly, we also designed RCT, see Title (Page 1, Line 2). Overall, while the pandemic presented significant challenges, we made efforts to understand and mitigate its impact on our trial.

2. The connection between theta brainwaves and anxiety needs to be supported by additional clinical studies. Please provide a more detailed discussion of the existing literature on this relationship, offering insights into how theta oscillations are implicated in anxiety disorders.

Authors: By reviewing the literature, we combed through some preliminary links between theta brain waves and anxiety, as follows:

Several studies have shown that alterations in theta brainwave activity can be associated with anxiety disorders. In healthy individuals, theta oscillations play important roles in memory formation, emotional processing, and attention. However, in those with anxiety disorders, there appears to be a dysregulation of theta activity. For example, some research has found that individuals with generalized anxiety disorder exhibit increased theta power in certain brain regions, such as the prefrontal cortex and hippocampus. This increased theta activity may be related to heightened emotional reactivity and rumination, which are common features of anxiety ^[21,22]^. Another line of research has investigated the role of theta oscillations in the amygdala, a key brain region involved in fear and anxiety. Studies have shown that abnormal theta activity in the amygdala may contribute to the development and maintenance of anxiety disorders^[23]^. Furthermore, some studies have used neurofeedback techniques to train individuals to modulate their theta brainwaves. Preliminary results suggest that by learning to control theta activity, it may be possible to reduce anxiety symptoms^[24]^. (Page 16, Line 306; Page 17, Line 307-320)

21.	Zhang Y, Lei L, Liu Z, Gao M, Liu Z, Sun N, et al. Theta oscillations: A rhythm difference comparison between major depressive disorder and anxiety disorder. Frontiers in Psychiatry. 2022;13:827536. [doi.org/10.3389/fpsyt.2022.827536](https://doi.org/10.3389/fpsyt.2022.827536)

22.	Hines M, Poulter S, Douchamps V, Pibiri F, McGregor A, Lever C. Frequency matters: how changes in hippocampal theta frequency can influence temporal coding, anxiety-reduction, and memory. Frontiers in Systems Neuroscience. 2023;16:998116. [doi.org/10.3389/fnsys.2022.998116](https://doi.org/10.3389/fnsys.2022.998116)

23.	Çalışkan G, Stork O. Hippocampal network oscillations at the interplay between innate anxiety and learned fear. Psychopharmacology. 2019;236(1):321-38.

doi.org/10.1007/s00213-018-5109-z

24.	Omejc N, Rojc B, Battaglini PP, Marusic U. Review of the therapeutic neurofeedback method using electroencephalography: EEG Neurofeedback. Bosnian journal of basic medical sciences. 2019;19(3):213. doi: [10.17305/bjbms.2018.3785](https://doi.org/10.17305/bjbms.2018.3785)

3. It is essential to hypothesize both potential positive and negative outcomes before initiating a clinical trial. In the current version, the introduction section primarily focuses on the probable positive effects of Tai Chi. Please ensure that the possibility of neutral or negative effects is also acknowledged.

Author: Some negative effects of TC have been added to our manuscript, as follows:

Furthermore, while TC is generally regarded as a safe practice, there exists a minor risk of injury. For example, participants may encounter muscle strains or joint discomfort if they execute the movements improperly or exert themselves excessively. Moreover, some individuals might perceive the time commitment associated with regular TC practice as burdensome, potentially leading to stress or dissatisfaction^[42]^. (Page 22, Line 419-424)

42.	Wayne PM, Berkowitz DL, Litrownik DE, Buring JE, Yeh GY. What do we really know about the safety of tai chi?: a systematic review of adverse event reports in randomized trials. Archives of physical medicine and rehabilitation. 2014;95(12):2470-83. [doi:10.1016/j.apmr.2014.05.005](https://doi.org/10.1016/j.apmr.2014.05.005)

4. The description of Tai Chi practice should be more detailed. Information regarding the timing of the sessions (e.g., morning vs. evening) and the standardization of the practice should be included. Furthermore, clarify how data was recorded, including any specific protocols or technologies used.

Authors: We have enhanced the description of Tai Chi exercises, including information on course scheduling and practice standardization. In addition, the data recording method is also clarified as follows:

The TC intervention, which was initiated in April 2022, comprised three weekly sessions over 12 consecutive weeks. To standardize the practice, two sessions of the 24-form simplified TC were instructor-led in the gym by a certified Tai Chi instructor at 4 pm on Tuesday and Thursday, and one session involved independent learning through videos at any free time, which was recorded for verification purposes. (Page7, Line 137-141)

In terms of data recording, several protocols and technologies were employed. Physical measurements such as heart ratewas taken before and after each session using standard medical devices. (Page7, Line 146-147) Additionally, subjective measures such as participant self-reports of stress levels was collected using validated questionnaires. (Page8, Line 162-163) To monitor adherence and intensity of the Tai Chi practice, video recordings were made of some sessions. These recordings were later analyzed by the research team to ensure that the participants were performing the movements correctly and with appropriate intensity. (Page7, Line 140-141) Moreover, a logbook was provided to each participant to record their daily practice and any notable experiences or changes. (Page7, Line 143-144)

5. The exclusion criteria need to be presented in more detail. For instance, did you exclude participants who were using antidepressant medications or receiving psychological interventions? Clearly specifying such criteria is crucial for understanding the generalizability of the findings.

Authors:  We further clarified and added exclusion criteria as follows:

Conversely, to isolate the effects of the intervention being studied and to avoid confounding variables, we did exclude participants who were currently using antidepressant medications or receiving formal psychological interventions, and participants who had certain pre-existing medical conditions that could potentially interfere with the study outcomes, such as severe neurological disorders or unstable chronic illnesses. We further excluded individuals who regularly engaged in professional exercises or with a reported history or risk of cardiovascular diseases or mental illness, and were unable to provide informed consent or who had language barriers that would prevent them from fully understanding the study procedures, contributing to the overall robustness and reliability of the study. By clearly defining and adhering to these exclusion criteria, the study population was made more homogeneous and the findings can be more accurately interpreted and potentially generalized to similar populations. (Page 6, Line 112-124)

6. If the Chinese version of the STAI was used, please provide information regarding its reliability, such as the Cronbach's alpha coefficient and any validation data for the scale within the study population.

Author: We have added the Cronbach's alpha of the STAI, as follows:

In this study, the Cronbach α of the STAI-S was 0.950, and the Cronbach α of the STAI-T was 0.926^[17]^. (Page 8, Line 169; Page 9, Line 170)

Du, Q., Liu, H., Yang, C., Chen, X., & Zhang, X. (2022). The development of a short Chinese version of the state-trait anxiety inventory. Frontiers in Psychiatry, 13, 854547.

7. According to your analysis, the correlation between anxiety levels and theta oscillatory power was relatively weak (with correlation coefficients ranging from 0.3 to 0.4). I recommend emphasizing this finding in the results section, including a more thorough interpretation of its significance.

Author: Thanks very much to the expert review, we have highlighted the weak correlation between anxiety levels and theta oscillation power in the results section, as follows:

and highlighted the relatively weak correlation (r: 0.3-0.4) between anxiety levels and theta oscillatory power. Firstly, while there was an observable connection between anxiety and theta oscillatory power, it was not as strong as might have been expected. This suggests that while theta oscillations may play a role in anxiety, other factors were likely also at play. It implied that anxiety was a complex phenomenon that cannot be solely attributed to changes in theta power. It also indicated that more comprehensive studies are required better to understand the relationship between brainwave activity and anxiety. Moreover, this finding should be considered when interpreting the results of interventions aimed at reducing anxiety through modulating theta oscillations. The relatively weak correlation suggests that such interventions may not significantly impact anxiety solely through changes in theta power. However, it does not rule out the possibility that they could still have some beneficial effects in combination with other approaches. (Page 18, Line 342-348; Page 19, Line 349-354)

8. In the discussion, you mention "college students amidst the challenges posed by the COVID-19 pandemic." Please describe in greater detail what specific challenges the students faced during the pandemic and how these may have impacted their mental health and engagement with the trial.

Authors: We have added more detailed descriptions of the specific challenges students faced during the pandemic and how these challenges may have impacted their mental health and participation in the trial, as follows:

Secondly, another significant challenge was the pandemic. Students experienced disruptions to their academic routines, transitioned to online learning, and had to adapt to new pedagogical methods and technologies. The uncertainty surrounding the duration and effectiveness of online education also contributed to heightened anxiety and stress; lockdowns, campus closures, and social distancing measures resulted in social isolation, increased feelings of loneliness, depression, and anxiety; furthermore, widespread economic pressures coupled with uncertainties regarding future employment prospects during the pandemic exacerbated feelings of anxiety and despair. These challenges directly impact students' mental health, which subsequently influences their participation in trials. Nevertheless, despite these adversities, many students exhibited resilience and adaptability by proactively seeking support from mental health resources while reinforcing their motivation to engage in trials. (Page 23, Line 438-449)

9. Please clarify how other potential confounding factors that could influence theta brainwaves were controlled or excluded in your analysis. Ensuring that these factors are adequately accounted for is vital to the study's internal validity.

Authors: In our study, we took several steps to control or exclude potential confounding factors that could influence theta brainwaves. First, we carefully selected our study participants to minimize the influence of pre-existing conditions. We excluded individuals with known neurological disorders, head injuries, or those taking medications that could potentially affect brainwave activity. This helped to ensure that any observed changes in theta brainwaves were more likely to be related to the variables of interest rather than pre-existing conditions (see Page 6, Line 112-121). Second, we controlled for environmental factors as much as possible. The study was conducted in a quiet, controlled environment to minimize external distractions and noise that could potentially affect brainwave recordings (see Page 9, Line 180-182). We also standardized the time of day for data collection to reduce the impact of circadian rhythms on brainwave activity (see Page 9, Line 182-190; Page 10, Line 191-196). To address potential psychological confounding factors, we administered questionnaires to assess stress levels (see Page 8, Line 162-163). These factors could potentially influence theta brainwaves, so by measuring and controlling for them, we were able to reduce their potential confounding effects. Furthermore, we used statistical methods to adjust for potential confounding variables. For example, we performed a covariance analysis to control for age, gender, which have been shown to correlate with brain wave activity (see Page 11, Line 221-222).

In conclusion, we took a comprehensive approach to controlling and excluding potential confounding factors that could influence theta brainwaves. By doing so, we believe we have enhanced the internal validity of our study and provided more reliable results.

Reviewer #3:

This study investigates the effects of a 12-week Tai Chi (TC) practice on the mental health of university students during the COVID-19 pandemic and presents significant data and findings. The research evaluates the impact of TC exercise on anxiety levels and brain theta oscillatory characteristics. Conducted as a randomized controlled trial, it also provides physiological data related to brain theta oscillation.

The article has been meticulously planned, implemented, and written. The findings and analyses presented are poised to make a significant contribution to the existing literature.

I believe this study will provide valuable insights in its field. In my opinion, the study is suitable for publication in its current form.

Authors: Thank you very much for your positive assessment. We are pleased to hear that you find our study suitable for publication. We believe that our research does have the potential to contribute to the field, and we are glad that you recognize its value. If there are any further suggestions or comments you may have to enhance the study even more, we would be happy to consider them.
